# Performance management of generalist care for hospitalised multimorbid patients—a scoping review for value-based care

**DOI:** 10.3389/frhs.2023.1147565

**Published:** 2024-02-26

**Authors:** Jia En Joy Khoo, Cher Wee Lim, Yi Feng Lai

**Affiliations:** ^1^ Ministry of Health (MOH) Office for Healthcare Transformation, Singapore, Singapore; ^2^ Department of Life Sciences, Faculty of Science, National University of Singapore, Singapore, Singapore; ^3^ Saw Swee Hock School of Public Health, National University of Singapore, Singapore, Singapore; ^4^ Department of Pharmacy, Faculty of Science, National University of Singapore, Singapore, Singapore; ^5^ School of Public Health, University of Illinois at Chicago, Chicago, IL, United States

**Keywords:** outcome measures, process measures, performance management, value-based care, generalist care, inpatient care, multimorbidity

## Abstract

**Objectives:**

Given the shift towards value-based healthcare and the increasing recognition of generalist care, enacting value-based healthcare for generalist care is critical. This work aims to shed light on how to conduct performance management of generalist care to facilitate value-based care, with a focus on medical care of hospitalised patients.

**Design and setting:**

A scoping review of published literature was conducted. 30 publications which were relevant to performance management of generalist medical inpatient care were included in the review.

**Outcome measures:**

The performance measures used across the studies were analysed and other qualitative findings were also obtained.

**Results:**

We report an overall lack of research on performance management methods for generalist inpatient care. Relevant performance measures found include both outcome and process of care measures and both clinical and reported measures, with clinical outcome measures the most frequently reported. Length of stay, readmission rates and mortality were the most frequently reported. The insights from the papers emphasise the relevance of process of care measures for performance management, the advantages and disadvantages of types of measures and provide suggestions relevant for performance management of generalist inpatient care.

**Conclusion:**

The findings of this scoping review outline a variety of performance measures valuable for generalist inpatient care including clinical outcome measures, reported outcome measures and process of care measures. The findings also suggest directions for implementation of such performance management, including emphasis on physician level performance management and the importance of documentation training. Further research for selecting and operationalising the measures for specific contexts and developing a comprehensive performance management system involving these measures will be important for achieving value-based healthcare for generalist inpatient care.

## 1 Introduction

Multimorbidity is on the rise globally, with ageing demographics as large driving factor particularly in high-income countries like Singapore (1). High rates of physician specialisation in hospitals have allowed for impressive capabilities in treating single diseases but will likely fall short in attending to the growing population of patients with multimorbidity (1). In such patients, fragmented care consisting of specialised treatment for each of the diseases in isolation will be inefficient and less effective (1, 2). Hence, a generalist skill set and holistic approach has been identified as crucial in the development of the future medical workforce to respond to the increasing incidence of multimorbidity (1, 2). At present, hospital care delivery is largely disease based rather than holistic (3). However, given the recognition of the importance of generalist care in responding to the rising multimorbidity, the proportion of generalist inpatient care practiced can be expected to increase.

The shift away from fee-for-service and towards value-based care is another trend in healthcare systems globally which aims to increase the quality of care while reducing costs (4). Value is often evaluated using the health outcomes of the patient against the cost (4). The performance of healthcare providers is based on the measured health outcomes, and renumeration is tagged to their performance (4). Value-based healthcare has resulted in changes to care delivery models towards a team-approach for improved care coordination and outcome measurement (4). Value-based care has begun to be widely implemented, through financing models such as pay-for-performance (P4P) schemes especially in primary care (5). In the United States, the shift towards value-based healthcare has seen the establishment of Patient Centred Medical Homes, Accountable Care Organisations and the Hospital Value-Based Purchasing Program (4).

In Singapore, the ageing population and increasing burden of multimorbidity has led to an increased implementation of integrated holistic generalist inpatient care. This is a shift from the initial single disease-based specialised approach which resulted from a time with younger population demographics and lower incidence of chronic diseases (3). One such implementation of generalist inpatient care is the Integrated General Hospital care model which was launched in 2018 (6). Following that, another acute hospital implemented a specialist-led General Medicine care model, reported in a 2021 publication (3, 7). Value-based care is also beginning to take root in the Singapore healthcare system. In 2019, the Value-Driven Care (VDC) program was introduced, which has led to an improvement in quality and reduction in costs [Unpublished source: MOH Clinical Quality, Performance and Value (CQPV) Brief on MOH Value Based Healthcare Efforts, 2017]. Under the program, standardised quality indicators for 17 high-impact surgical and medical conditions requiring inpatient care have been identified and the program aims to improve clinical outcomes while maintaining cost-effectiveness (8). The benchmarked data are used for regular engagements with the clinical workgroups and there is active sharing of best practices to increase value. Value-based payment models are also being implemented. (Unpublished source: MOH CQPV, 2017).

However, multimorbidity has presented itself as a challenge to the implementation of value-based healthcare. Models like P4P have been recognised to have a focus on the care of individual diseases and not multimorbidity (9). For example, the UK Quality and Outcomes Framework which is largest P4P program globally mainly operates on a single-disease basis (9). A similar characterisation can be made of Singapore's VDC program. Outcome measurement and evaluation, an integral part of value-based care implementation, is a challenge when it comes to multimorbidity (10–12). A lack of outcome measures tailored for such contexts has been cited (10–12). The inpatient setting is also an area where the implementation of value-based care has fallen behind. In a review of studies that evaluated P4P programs published in 2017, only 11 out of 69 studies identified took place in inpatient settings while 58 were in ambulatory settings (13). Another review published in 2016 identified 34 P4P programs in the inpatient sector across 24 OECD countries to review (14). A review published in 2019 assessing the impact of P4P on quality of care in an inpatient setting included 27 studies on 6 P4P programs, where the studies compared it to a basic payment scheme (15). In response to these challenges, there have been studies that aimed to shed light on outcome measurement for multimorbidity (10–12). Some studies, including review papers, have sought to review outcomes for multimorbid patients, but with a focus on the primary care space, or in general (10–12). We aim to contribute to this body of work by conducting a scoping review to describe how performance management can be done for implementing generalist-led value-based care for multimorbid patients, but with a focus on the inpatient setting.

This work will respond to the generalist inpatient gap in the current disease specific, ambulatory care focused implementation of value-based care globally. The insights will contribute to value-based care being implemented comprehensively, including in generalist care which is only going to increase in importance and urgency as multimorbidity rises. The scoping review will identify the level of existing research in this area and areas lacking evidence that will need to be addressed to enable the future implementation of generalist-led inpatient care under a value-based care framework. By surveying existing research on performance management in this field, we hope to provide insights to inform implementation of such a care model.

## 2 Methods

### 2.1 Study design

A scoping review was conducted and reporting was completed with the PRISMA extension for Scoping Reviews (PRISMA-ScR) as guidance (Supplementary Appendix 1). This review's protocol was not pre-registered or published.

### 2.2 Data sources and search

To identify the relevant literature, SCOPUS (2006 to 31 June 2021), MEDLINE, CINAHL and Web of Science (2006 to 29 June 2021) electronic databases were searched. The search strategies were drafted by the first author and adopted by the team by consensus. The full search strategies are reported in Supplementary Appendix 2. The key search terms were related to the concepts of generalist-led care, multimorbidity, value-based care and performance management.

The review included only published literature with publication dates from 2006 onwards, which was the year Porter and Teisberg published their book “Redefining Health Care”, which introduced the concept of value-based healthcare (16), that prompted the international shifts towards such a framework. There was no restriction based on the type of publication or study design. Conference abstracts were included. The papers included had a focus on evaluating generalist medical care in the inpatient setting. Such generalist care was defined to include inpatient care by internal medicine (IM) physicians, hospitalists, family medicine physicians or geriatricians, and either this or the setting of an internal medicine/general medicine ward or geriatric ward should be stated. The less common scenario of generalist care provided by specialist physicians could also be included. Sub-acute care was also considered generalist medical care. Involvement of generalist physicians that had some relation to surgical care, such as in perioperative care, was excluded. Papers were excluded if they did not fit the focus of the review, such as if the focus was not on medical generalist care, not clearly inpatient specific, lacked relevant performance management focus, focused on care by non-physician providers or focused on transitional care. Papers with a focus on specific patient groups, including patients with specific conditions or patients with dementia were excluded, as that lies beyond the scope of this review which aims to guide the performance management of inpatient generalist care for a general medical population. Papers were also to be excluded if the full text was not in English or could not be accessed, except for conference abstracts which could be included.

### 2.3 Selection of sources of evidence

Duplicates identified by reference manager software EndNote X9 were first removed. Following this, the titles and abstracts were screened by the first author to identify articles to proceed to full-text screening. These full texts were then reviewed for relevance and eligibility. All stages of screening were performed by the first author, in consultation with the last author.

### 2.4 Data charting

The charting form was developed by the first author and after limited initial charting and review by the last author, small refinements were made to the form. The data charting was done by the first author. The form was used to extract information about the study characteristics (e.g., purpose of study, country), the context (e.g., study population/setting), the evaluation measures used and other relevant findings. The evaluation measures used were organised based on process of care and outcome measures. The outcome measures were further categorised into clinical measures, reported measures, cost outcomes, other outcomes (e.g., resource utilisation). If certain measures [e.g., length of stay (LOS)] that are often considered clinical outcomes were specified to be a measure of for instance, resource utilisation, healthcare utilisation or productivity, this was followed in the charting. Other measures that were non-outcome indicators of ward/hospital performance or management were also recorded. Covariates that were adjusted for in analysis of the measures were also recorded when it was clearly listed. The process and outcome measures were tabulated based on frequency of occurrence among the studies reviewed. Cost measures were not a focus of the study and were in their own category although they are often considered resource utilisation.

### 2.5 Patient and public involvement statement

Patients and public were not involved in the research given its nature as a scoping review.

## 3 Results

### 3.1 Literature search/selection of sources of evidence

**Figure 1:**
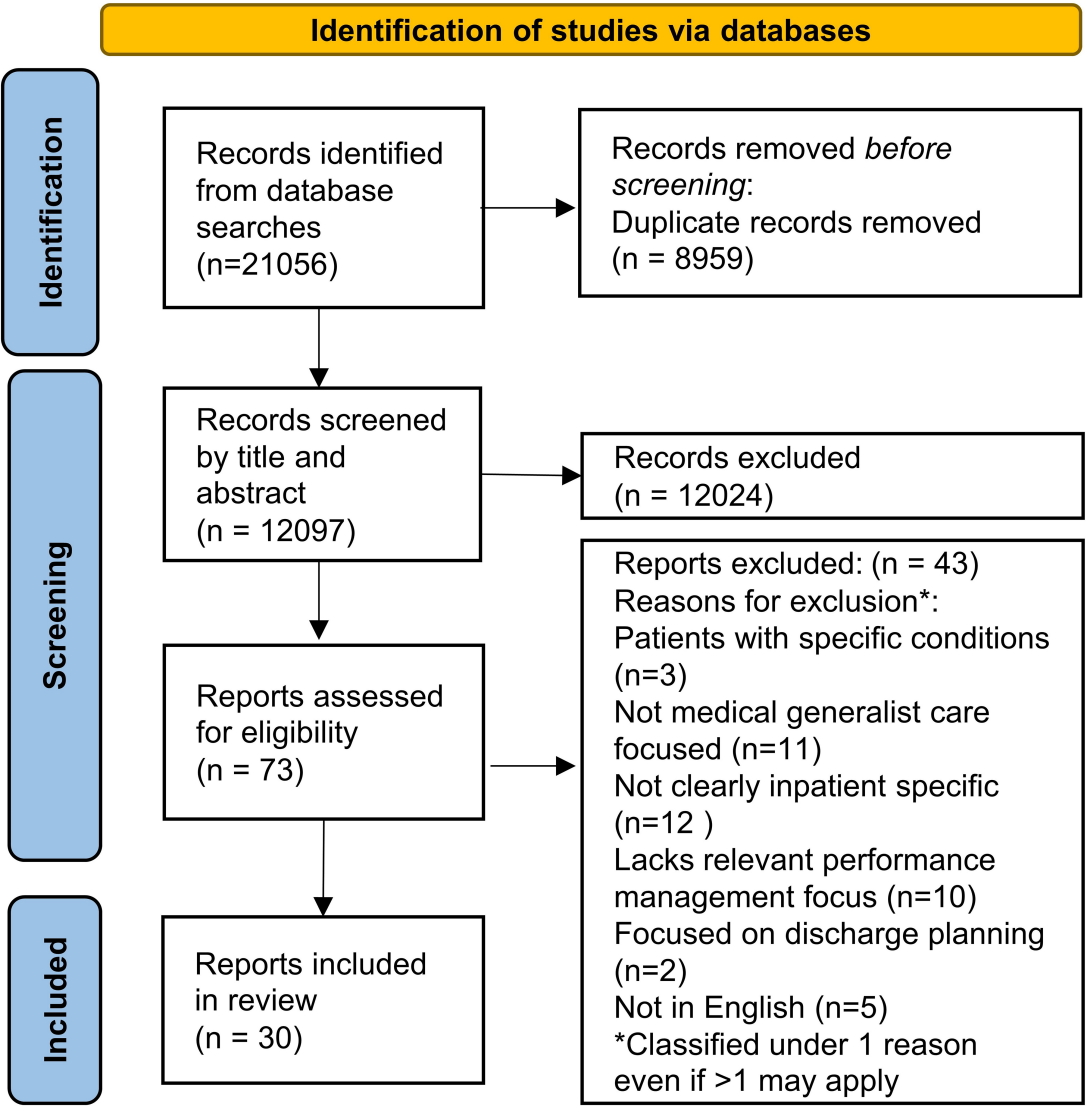
PRISMA flow diagram.

As seen in Figure 1, 21,056 records were obtained from the database searches. 8959 duplicates were identified by the EndNote software and removed with 12,097 records remaining. These were screened based on the titles and abstracts (where available). 12,024 were excluded and 73 were selected for full-text screening. Out of these, 43 were excluded because they had a focus on patients with specific conditions, did not clearly have an inpatient setting, was not in English, lacks relevant performance management focus (on specific methods of performance management or evaluation of generalist inpatient care), lacks relevant focus on medical generalist-led care or focus was on discharge planning. 30 unique publications remained for data charting.

### 3.2 Characteristics of sources of evidence

Table 1 lists the studies included in the review and their aims. Information on their settings/study populations is in Supplementary Appendix 3. A total of 30 unique publications were included.

**Table 1:** Characteristics of studies included.

Index	Title	Country	Purpose
1	Association between treatment by locum tenens internal medicine physicians and 30-day mortality among hospitalized medicare beneficiaries (17)	United States	Evaluate quality and costs of care among hospitalized Medicare beneficiaries treated by locum tenens versus non–locum tenens physicians.
2	Challenges and opportunities in pragmatic implementation of a holistic hospital care model in Singapore: A mixed-method case study (3)	Singapore	Quantitatively summarise the clinical outcomes of a specialist-led General Medicine model implemented at an acute hospital in Singapore and qualitatively describe the challenges and learnings.
3	Comparison of resident, advanced practice clinician, and hospitalist teams in an academic medical centre: association with clinical outcomes and resource utilization (18)	United States	Directly compare outcomes among resident, advanced practice clinician (APC), and solo hospitalist inpatient general medicine teams.
4	Development of resident-sensitive quality measures for inpatient general internal medicine (19)	United States	Develop Resident-Sensitive Quality Measures for a general internal medicine inpatient residency rotation using previously established consensus methods.
5	Investigating the effectiveness of care delivery at an acute geriatric community hospital for older adults in the Netherlands: a protocol for a prospective controlled observational study (20)	Netherlands	Compare care for older patients in the geriatrician-led acute geriatric community hospital versus in a hospital setting. This includes evaluating patient outcomes and cost effectiveness and identifying facilitators and barriers to the implementation.
6	A novel organizational model to face the challenge of multimorbid elderly patients in an internal medicine setting: a case study from Parma Hospital, Italy (21)	Italy	Present an organizational model at the Internal Medicine and Critical Subacute Care Unit of Parma University Hospital which is a 106-bed internal medicine area organized by intensity of care and dedicated to multimorbid elderly patients.
7	Selecting best-suited “patient-related outcomes” in older people admitted to an acute geriatric or emergency frailty unit and applying quality improvement research to improve patient care (22)	United Kingdom	A review to explore multimorbidity, frailty, the service model, and a range of “direct” and “indirect” patient-related outcomes of “at risk” older people admitted to an acute geriatric care unit. Data measurements and discussion of a quality improvement methodology to improve patient care based on patient-related outcome data.
8	Variation in physician spending and association with patient outcomes (23)	United States	Investigate variation in spending across physicians and the association with patient outcomes.
9	Census of Ligurian internal medicine wards of non-teaching hospitals (24)	Italy	Survey the activities of hospital internal medicine to: i) define and compare the activities of each ward; ii) assess the impact of these activities on business management; iii) define the role of departments of internal medicine in the hospital organization; iv) formalize the role of emergency medicine and internal medicine v) provide tools of clinical governance in the definition of standards. Data was collected about medical staffing, equipment, skills, competencies and productivity.
10	Pocket change: a simple educational intervention increases hospitalist documentation of comorbidities and improves hospital quality performance measures (25)	United States	Designed a pocket card reminder and educational intervention for hospitalists to improve documentation of 6 common comorbidities present on admission. It was hypothesized that having this intervention in addition to just review by documentation specialists would be superior in capturing comorbidities present on admission and improving expected length of stay (LOS) and expected mortality.
11	Using assessing care of vulnerable elders quality indicators to measure quality of hospital care for vulnerable elders (26)	United States	Assess quality of care for hospitalized vulnerable elders using measures based on Assessing Care of Vulnerable Elders (ACOVE) quality indicators.
12	Two European examples of acute geriatric units located outside of a general hospital for older adults with exacerbated chronic conditions (27)	Europe	This study aims to compare patients’ diagnoses, characteristics, and outcomes of 2 European sites of acute geriatric units in intermediate care outside a general hospital, where acute medical care and early rehabilitation are provided to selected older patients.
13	Short-term resource utilization and cost-effectiveness of comprehensive geriatric assessment in acute hospital care for severely frail elderly patients (28)	Sweden	Estimate the 3-month within-trial cost-effectiveness of comprehensive geriatric assessment (CGA) in acute medical care for frail elderly patients compared to usual care, by estimating health-related quality of life and costs from a societal perspective.
14	RECALMIN: The association between management of Spanish national health service internal medical units and health outcomes (29)	Spain	Investigate the association between management of Internal Medical Units (IMUs) with outcomes (mortality and length of stay).
15	Quality of care factors associated with unplanned readmissions of older medical patients: a case-control study (30)	Australia	Assess the type and prevalence of quality of care factors associated with potentially preventable readmissions at a tertiary hospital general medicine service. Identify patient characteristics associated with quality factors.
16	Quality of care delivered by general internists in US hospitals who graduated from foreign versus US medical schools: observational study (31)	United States	Determine if patient outcomes differ between general internists who graduated from a medical school in the United States and outside the United States.
17	Evaluating outcomes from an integrated health service for older patients (32)	Australia	Compare the demographics and outcomes of patients from the Older Person Evaluation Review and Assessment (OPERA) service to those from the General Medicine (GM) service to contribute to a baseline for future analysis and to quantify the benefits of a dedicated CGA model for the management of older patients with acute illness. The OPERA service ran parallel to the general medicine service, offering alternative treatment with more comprehensive care led by a geriatrician. It focused on integrating the multidisciplinary needs of older patients requiring acute hospitalisation and providing support and intervention after discharge.
18	Relationship between quality of care and functional decline in hospitalized vulnerable elders (33)	United States	To assess the relationship between process-of-care-based quality indicators for hospitalized elders and functional decline.
19	Patient experiences and predictors in an acute geriatric ward: A cross-sectional study (34)	Norway	To investigate the elderly's experiences with acute hospital treatment and care, and the influence of socio-demographic variables, length of stay, comorbidity and self-rated health on these.
20	Patient characteristics, resource use and outcomes associated with general internal medicine hospital care: the General Medicine Inpatient Initiative (GEMINI) retrospective cohort study (35)	Canada	Describe the demographic characteristics, medical conditions, health outcomes and resource use of patients admitted to general internal medicine at 7 hospital sites.
21	Implementation of a physician assistant/hospitalist service in an academic medical centre: impact on efficiency and patient outcomes (36)	United States	Evaluate and compare the quality and efficiency of patient care on a physician assistant/hospitalist service with traditional house staff services.
22	Impact of hospitalists on care outcomes in a large integrated health system in British Columbia (37)	Canada	Study care outcomes associated with a network of hospitalist services compared to “traditional” providers (community-based Family Physicians and Internal Medicine specialists) in a large integrated health care system.
23	Similar outcomes among general medicine patients discharged on weekends (38)	Canada	Determine whether post discharge outcomes differed for patients discharged on weekends where there are reduced staffing levels and services versus weekdays.
24	Family medicine patients have shorter length of stay when cared for on a family medicine inpatient service (39)	United States	Test the hypothesis that local family medicine patients admitted to a family medicine inpatient service have shorter length of stay than those admitted to general hospitalist services which also care for tertiary patients at an academic medical centre.
25	Does doctor–patient communication affect patient satisfaction with hospital care? Results of an analysis with a novel instrumental variable (40)	United States	Determine the relationship between physicians’ communication behaviours and patients’ overall satisfaction with hospital care using a novel instrumental variable to address possible confounding of this association by patient attributes.
26	Relationship between quality of care for hospitalized vulnerable elders and post-discharge mortality (41)	United States	Assess the relationship between quality of hospital care, as measured by ACOVE quality indicators, and post-discharge mortality for hospitalised elderly patients.
27	Improving patient care chronic complex: sub-acute care unit (42)	Spain	Description of the general characteristics of the sub-acute care of Vallparadís Healthcare and their assistance activity.
28	Analysis of casuistry and results of the implementation of a care program in ten sub-acute care units in Catalonia (43)	Spain	Appraise the improvement in care for people with complex chronic illnesses, through the implementation of sub-acute care units for intermediate care. There was comprehensive and integrated on-going care and resources were adapted to patient needs. The care process, outcomes and their adjustment to the standards established were analysed.
29	Patient outcomes in teaching versus nonteaching general internal medicine services: a systematic review and meta- analysis (44)	United States	Conduct a systematic review and meta-analysis to examine if outcomes differ for general internal medicine (GIM) patients admitted to teaching versus nonteaching services.
30	Effects of an acute care for elders unit on costs and 30-day readmissions (45)	Unites States	To examine variable direct costs from an interdisciplinary Acute Care for Elders (ACE) Unit compared with a multidisciplinary usual care (UC) unit which are both hospitalist medical units.

Only 2 publications were aiming to directly answer how to measure the quality of generalist care (19, 26). One was focused on elderly patients (26). Both presented a selected list of process measures to be used in evaluating quality of care (19, 26).

There was 1 other publication that also curated a list of process measures as quality of care factors but in order to study the association with unplanned readmissions (30). This was focused on elderly patients. There were 2 other publications that were also from the angle of how quality of care affects certain outcome measures (33, 41). The set of quality of care factors reported in the previous study (26) were used for these 2 studies which were similarly focused on elderly patients (33, 41).

A descriptive review aimed to directly explore the range of possible patient-related outcomes for “at risk” elderly patients in acute geriatric care units (22). The authors were interested in these outcomes in the context of quality improvement (22).

There were 21 publications that generally took the angle of how a specific implementation (or aspect such as physician spending or doctor-patient communication) of generalist care affects outcomes or quality of care (3, 17, 18, 20, 21, 23, 27–29, 31, 32, 34, 36–40, 42–45). Eleven were focused on elderly patients (17, 20, 21, 23, 27, 28, 31, 32, 34, 43, 45).

There were 2 publications that aimed to describe the activities and characteristics of a group of internal medicine wards across hospitals, and this involved some outcome measures (24, 35).

Lastly, there was a publication that focused on improving documentation accuracy to aid in accurate performance management (25).

### 3.3 Results of sources of evidence

The outcome and process of care evaluation measures used in each publication are reported in Supplementary Appendix 4. Twenty-four reported only outcome measures (3, 17, 18, 20, 21, 23–25, 27–29, 31, 32, 34–40, 42–45), 4 reported outcome and process measures (22, 30, 33, 41), while 2 reported only process measures (19, 26). Supplementary Appendix 5 reports the qualitative insights from each publication.

#### 3.3.1 Outcome measures

Table 2 shows a summary of the outcome measures used across the publications. The most common outcome measures used were the mortality and readmission clinical measures, which were used in 20 and 19 unique publications respectively. This is followed closely by length of stay measures, used in 18 unique publications. In comparison, the non-clinical, reported measures were used much less frequently. Patient reported measures were used in 9 of the publications. Patient satisfaction, quality of life and Activities of Daily Living (ADLs) which were mentioned the most frequently, were each only in 3 unique publications each (Table 2).

**Table 2:** Summary of outcome measures.

Category	Measure	Indexes	No. of unique publications
Clinical	Mortality	1, 2, 3, 5, 6, 7, 8, 12, 13, 14, 16, 17, 20, 21, 22, 23, 27, 28, 29, 30	20
Clinical	Readmission	1, 2, 3, 5, 6, 7, 8, 15, 16, 17, 20, 21, 22, 23, 24, 27, 28, 29, 30	19
Clinical/resource utilisation/ward productivity/efficiency	Length of stay	1, 2, 3, 5, 6, 9, 12, 13, 14, 17, 20, 21, 22, 24, 27, 28, 29, 30	18
Clinical/patient reported	Fall related	5, 7	2
Clinical	Other adverse effects	5, 7, 15	3
Clinical/healthcare utilisation	Discharge destination	5, 7, 12, 27, 30	5
Clinical/patient reported	Quality adjusted life years	13	1
Clinical	Time to death after discharge	26	1
Clinical	Need for a type of care package, increased dependency or mobility	7	1
Clinical	Days at residence post-discharge	7	1
Patient reported	Activities of daily living	5, 18, 28	3
Patient reported	Pain	5	1
Patient reported	Fatigue	5	1
Patient reported	Quality of life	5, 7, 13	3
Patient reported	Patient satisfaction	5, 22, 27	3
Patient reported	Patient experience	19, 25	2
Patient reported	Emotional distress/psychological burden	5, 7	2
Caregiver reported	Caregiver burden	7	1
Caregiver reported	Caregiver satisfaction	5	1
Staff reported	Staff experience	7, 22	2
Costs	Costs	1, 2, 3, 5, 8, 9, 13, 16, 20, 21, 29, 30	12
Resource/healthcare utilisation	Index admission resource utilisation (other than length of stay and transfer to higher acuity care)	3, 5, 20	3
Clinical/healthcare utilisation	Transfer to higher acuity care	5, 12, 20, 21	4
Healthcare utilisation	Emergency department visits	5	1
Resource/healthcare utilisation	Outpatient visits	5, 13	2
Resource utilisation	Inpatient care post-discharge	13	1
Resource/health utilisation/other	Home care	5, 13	2
Resource utilisation	Nursing care post-discharge	13	1
Resource/healthcare utilisation	Primary care	5, 7, 13	3
Healthcare utilisation	Allied healthcare post-discharge	5	1
Resource/healthcare utilisation	Institutionalisation	5, 13	2
Others	Complaints and litigation costs	7	1

Caregiver burden was suggested for evaluation by 1 publication while staff related evaluation measures were mentioned in 2, from measuring self-reported resident work hours, to staff surveys of the work experience. Cost measures were mentioned in 12 unique publications (Table 2).

A more detailed synthesised list of the non-process measures can be found in Supplementary Appendix 6, including the time periods and scales used. The outcome measures belong to various categories including clinical and patient reported measures, caregiver reported and staff or cost outcomes. The same measure may belong to more than one category. For example, length of stay may be considered clinical or a resource utilisation measure, depending on the paper. It should be noted that the measures ultimately used in the respective evaluations are processed and adjusted versions, a simple example being taking the average of the length of stay. The synthesis of outcome measures did not take this into account. The data charting included some simple processed versions like average length of stay but not further processing.

##### 3.3.1.1 Significance of outcome measures

Although patient outcome measures are key for performance management, it is important to note that they may not be representative of quality of care. Readmissions of elderly patients in particular was noted as not a good indicator of quality of care, due to multiple confounding factors. Scott et al. noted that readmissions despite quality care are common in frail elderly with complex needs and comorbidities, and hence are not a good indicator of quality of care although he found that readmissions of older medical patients were associated with more quality-of-care problems (30). This association also does not imply a causal relationship (30). Implementing penalties to lower their readmission rates may be counterproductive, leading to other adverse outcomes (30). Short-term hospital readmissions have been noted to be “strongly related to baseline conditions and clinical course and independent of LOS and organisation of care” (21). Arora et al. found that for hospitalised vulnerable elders, quality of care factors was associated with lower mortality one year post-discharge (41). Nutritional status assessment in particular had a strong association (41). Notably, a delayed time frame (>500 days) has been known to produce the most significant relationship between quality of care and mortality (41). However, the association found by Arora et al. does not imply a causal relationship and further research is needed to verify that (41). Yousefi et al. and McAlister et al. both emphasised that post-discharge death or readmission is not necessarily reflective of the quality of inpatient care as there are other influencing hospital or community factors as well as diagnosis (37, 38).

A study found that management practices such as a patient safety committee or multidisciplinary ward rounds was only loosely associated with lower LOS and mortality, and had no independent effect on health outcomes when introduced as risk factors in regression models (29). It was noted that management of the units interacts with structural, management and complexity variables of hospitals (29). The authors also highlighted that “defining the performance of a medical unit is a complicated task involving multiple disciplines and approaches”, and there are other outcome-related variables they did not include (29). The complexity of performance management at the level of medical units is evident.

Outcomes like functional decline were also not found to be significantly associated with quality of care for hospitalised vulnerable elderly, although factors involved in the etiology of functional decline are addressed by those quality of care factors (33). In fact, certain quality of care factors, like efforts to improve mobility, were associated with functional decline (33). Post-hospitalisation care may be more associated with functional decline (33). There were various limitations in this observational study and further research in particular randomised controlled trials (RCTs) are required to determine the effect of quality of care on functional decline (33).

However, there may be certain more specific outcome measures which could better reflect quality of care. Scott et al. also found that readmissions within a week post-discharge as opposed to more distant readmissions, are more likely indicative of sub-optimal peri-discharge care, rather than being due to the disease severity (30). This may appear to differ from Meschi et al.'s statement that short-term readmissions are independent of organisation of care (21), but it is likely that Meschi et al. was not referring to a period as short-term as one week post-discharge. Further research should be done to identify such specific measures.

Related to outcome measures not necessitating quality of care, improvement in certain measures used in administrative performance indexes, like LOS, “does not necessitate direct positive patient outcomes” reflected in other outcome measures, although there may be theoretical benefits like the psychosocial and clinical benefits of shorter length of stay (21). LOS for elderly patients were found to not be associated with readmission rates (21).

##### 3.3.1.2 Making appropriate adjustments

It is important for the appropriate adjustments to be made in using these measures for performance management. In many of the studies, a range of covariates were adjusted for, as reported in Supplementary Appendix 4. Covariates can have significant associations with the outcomes, such as the covariate of sex and the outcome of mortality in a study (41). Singh and Aithal emphasised that clinical outcomes must be interpreted with appropriate patient characteristics, and provided an extensive list ranging from demographic markers to reason for admission and nutritional status (22). In a study by Yousefi et al., associations between hospitalist care and outcome measures were no longer present consistently when patients with specific diagnoses were compared (37). McAlister et al. highlighted that they were unable to adjust for post-discharge follow up but suggested that this would be beneficial as it could be associated with improved outcomes (38). Disease-specific outcome adjustments or a larger sample size have also been suggested to obtain clearer evidence on the association of the management of medical units and the outcomes (29).

Patient-reported measures also have many variables that need to be adjusted for. For example, patient satisfaction with hospital care can be confounded by level of health and socioeconomic level (40). However, “patient satisfaction surveys that request ratings are inherently confounded by expectations”. It was suggested that “experience-like” questions, like “would you recommend this hospital to a friend or relative” might alleviate this issue (40). The study could not determine if physician characteristics like technical behaviour may affect patient ratings (40).

##### 3.3.1.3 Selecting outcome measures

Arora et al. stated that mortality may not be the most relevant patient outcome for older patients, noting that older patients often value quality of life over length, although mortality was used in that study (41). Hence, the outcome measures selected should be adapted based on the patient group or weighted accordingly to ensure they are appropriate.

It was noted that patient-related outcomes should have a broader assessment of the impacts, including direct clinical outcomes at the patient level and indirect clinical outcomes at staff, family and carers, and community and organisational levels (22). The “best-suited” measures should be selected (22). Clinical outcome data should be regularly used to benchmark acute geriatric services while standardised, validated tools should be used to measure the indirect clinical outcomes (22). Staff-related outcomes are relevant in evaluating clinical outcomes due to the associations between staffing and patient-related outcomes (22). Caregiver burden is also associated with adverse clinical outcomes (22). Quality of patient care has been defined in terms of effectiveness of the care, patient safety, and patient experience (22). Organisational-level outcomes should be included as patient-related outcomes impact the organisation's performance (22). At the patient level, Patient-Reported Experience Measures (PREMs) are increasingly used and could be helpful in improving quality of care (22). Patient-Reported Outcome Measures (PROMs) are common in research but not in clinical practice to improve quality of care, although it has been noted that routine use of PROMs “improves decision-making between doctors and patients and improves patient care” (22). PROMs are validated tools that define functional status or health-related QoL among others and are relevant in evaluating care models or providers (22).

Patient ratings of the physician's communication behaviours was found to be significantly correlated with patient satisfaction which is in turn associated with changes in patient behaviour and health outcomes (40). Patient satisfaction is recognised as an important measure and a goal in healthcare that resources have been channelled towards (40). Sanahuja et al. linked patient satisfaction to indicating the quality of care (42). Physician communication behaviours could possibly be a patient reported measure to monitor as well. It was noted that there is much room for improvement in this measure (40).

##### 3.3.1.4 Inaccuracies in outcome measures

Self-reported measures such as of the patient experience can be subject to inaccuracies, particularly when such reporting is done after discharge which increases the risk of recall bias (34). Other factors that can affect the accuracy could include the presence of comorbidity which was found to have had a statistically significant negative influence on the Picker Patient Experience Questionnaire-15 (PPE-15) measure (34).

It is also important to account for natural fluctuations when defining the outcome of interest. Arora et al. noted that if they were to define functional decline as a single new ADL deficit, it may be inaccurate as such changes may occur naturally (33). Hence, the measure “catastrophic functional decline” was used which involves more drastic changes in ADL that are less likely to be due to natural fluctuations (33).

##### 3.3.1.5 Sources of outcome measures

Data for some measures were less frequently reported due to not being captured in administrative reports (21). One study justified the lack of data on in-hospital adverse events and negative consequences of hospitalisation (decline in physical performance, cognitive impairment, delirium, malnutrition and polypharmacy) in this manner (21). A review paper stated that “most studies used administrative data, which precludes fully adjusting for severity of disease or functional status” (44). The accuracy of data from regional administrative databases was also noted to depend on the Hospital Discharge Record by physicians (21). In another study, it was stated that comparisons to medical record data helped validate the use of administrative records to estimate outcomes of health services (29). The Minimum Basic Data Set (MBDS) data is subject to auditing and is valid, and there were no significant discrepancies with the data provided by the Internal Medical Units (29). Such studies are reliable for public comparison of outcome data of hospitals (29).

#### 3.3.2 Process of care measures

For process measures, 5 out of the 6 papers that included process measures were focused on elderly patients. Notably, the quality indicators for assessing the quality of hospital care of vulnerable elders presented by Arora et al. in 2007 was used in 2009 and 2010 publications by the same author, also included in this review (26, 33, 41). In Table 3, the process outcomes are classified based on the dimensions of healthcare quality by the World Health Organisation (WHO) (46). Supplementary Appendix 4 includes the full list of process measures for GIM/general hospital care from each source. However, some apply to patients with specific conditions although classified under GIM (19). In Table 3, only process measures for general care and geriatric conditions were included but not other specific conditions (e.g., congestive heart failure). Apart from an absence of measures regarding equity, the number of unique publications that used processes of care relating to the various dimensions was similar, ranging from 4 to 5 papers. Some measures did not clearly fit into the WHO quality dimensions. Safety and accessibility had the greatest number of measures, 12 and 11 respectively. The others only had 3–4 measures each.

**Table 3:** Process measures.

Dimension of healthcare quality	Measure	Indexes
Accessibility	Access to social workers and intermediate care services for an AGU	7
Accessibility	All vulnerable elders should be screened for chronic pain during the initial evaluation period.	11, 18, 26
Accessibility	If a vulnerable elder is admitted to the hospital for any acute or chronic illness or any surgical procedure, then the evaluation should include, within 24 h, cognitive status.	11, 18, 26
Accessibility	If a vulnerable elder has dementia, then he or she should be screened for depression during the initial evaluation.	11, 18, 26
Accessibility	If a vulnerable elder is found to have problems with gait, strength, or endurance, then an exercise program should be offered	11, 18, 26
Accessibility	Inadequate patient/carer education about clinical management of disease	15
Accessibility	Failure to develop/activate advance care plan	15
Accessibility	Failure to develop/activate palliative care plan	15
Accessibility	Failure to refer to chronic disease management/outreach service where indicated	15
Accessibility	Failure to refer to rehabilitation program (excluding geriatric rehabilitation) where indicated	15
Accessibility	Failure to arrange required home assistance and community support	15
		5 unique publications
Effectiveness	Screening of delirium, appropriate treatment, and communication of the diagnosis to community physicians	7
Effectiveness	Appropriate dementia care plan and staff training	7
Effectiveness	If a hospitalized elder has a definite or suspected diagnosis of delirium, then an evaluation for potentially precipitating factors must be undertaken and identified causes treated.	11, 18, 26
Effectiveness	If a vulnerable elder is admitted to a hospital or is new to a physician practice, then assessment of functional status should be documented.	11, 18, 26
Effectiveness	If a vulnerable elder presents with a pressure ulcer, then the pressure ulcer should be assessed for location, depth and stage, size, and the presence of necrotic tissue.	11, 18, 26
Effectiveness	If a vulnerable elder presents with a full-thickness sacral or trochanteric pressure ulcer covered with necrotic debris or eschar, then debridement using sharp, mechanical, enzymatic, or autolytic procedures should be done within 3 days of diagnosis.	11, 18, 26
Effectiveness	If a vulnerable elder has a stage 2 or greater pressure ulcer, then a topical antiseptic should not be used on the wound.	11, 18, 26
Effectiveness	If a vulnerable elder presents with a clean full-thickness or partial-thickness pressure ulcer, then a moist wound-healing environment should be provided with topical dressings.	11, 18, 26
Effectiveness	Diagnostic error or failure to diagnose	15
Effectiveness	Failure to assess or manage active comorbid disease	15
Effectiveness	Suboptimal management of primary clinical problem during admission	15
Effectiveness	Inadequate assessment of needs and limitations	15
Effectiveness	Failure to organise appropriate medical follow up	15
		5 Unique Publications
Efficiency	Factors leading to a delayed transfer of care in the community	7
Efficiency	If a vulnerable elder is admitted to the hospital, then the discharge planning should begin within 48 h.	11, 18, 26
Efficiency	Failure to communicate discharge information to post-hospital care providers	15
		5 unique publications
Patient-centredness	Document goals of care discussions that result in code status changes	4
Patient-centredness	Safe discharge planning and follow-up before discharge from an AGU with adequate involvement of patient and carer	7
Patient-centredness	If a vulnerable elder with dementia is to be physically restrained in the hospital, then the target behavioural disturbance or safety issue justifying use of the restraints must be identified to the consenting person and documented in the chart.	11, 18, 26
Patient-centredness	If a vulnerable elder is physically restrained and the target behavioural disturbance requiring restraint is identified, then the healthcare team should include methods other than physical restraints in the care plan.	11, 18, 26
		5 unique publications
Safety	Complete admission medication reconciliation	4
Safety	Complete discharge medication reconciliation	4
Safety	Document reason for not ordering deep venous thrombosis prophylaxis on admission	4
Safety	Medications listed in the discharge instructions match the discharge medication reconciliation	4
Safety	Medications listed in the discharge instructions match the discharge summary	4
Safety	Medications listed in the discharge summary match the discharge medication reconciliation	4
Safety	Order deep venous thrombosis prophylaxis on admission	4
Safety	Order seizure precautions in patients with seizure history or condition placing them at risk of seizure	4
Safety	Order suicide precautions for patients with self-harm or intentional ingestion	4
Safety	If a vulnerable elder is at very high risk for venous thrombosis, then the patient should have venous thromboembolism prophylaxis.	11, 18, 26
Safety	If a vulnerable elder is hospitalized, then his or her nutritional status should be documented during the hospitalization by evaluation of oral intake or serum biochemical testing.	11, 18, 26
Safety	If a vulnerable elder is admitted to an intensive care unit or a medical or surgical unit of a hospital and cannot reposition himself or herself or has limited ability to do so, then risk assessment for pressure ulcers should be done on admission.	11, 18, 26
		4 unique publications
Others	Document code status on admission	4
Others	Document source of positive blood culture specimens	4
Others	Order code status on admission	4
Others	Update sign out document daily	4
Others	Strategies and plans for carer assessment and engagement	7

##### 3.3.2.1 Suggested quality indicator sets

Process measures should also be monitored as part of performance management for generalist care. Most pay-for-performance and public reporting programs use quality of care measures that focus on specific processes of care (33). A sub-set of Assessing Care for Vulnerable Elders (ACOVE) quality indicators and a set of resident-sensitive quality measures (RSQMs) were two key sets of indicators that were selected by researchers who aimed to evaluate the quality of generalist inpatient care for vulnerable elders in the former (26), and with a focus on resident performance in the latter (19). Both consist of process measures (19, 26), which has been defined as “evidence-based elements of patient care that do not directly assess the patient's clinical condition” (33). A list of process measures to assess was also curated to assess quality of care in a study that investigated the association between quality of care and unplanned readmissions (30).

The ACOVE quality indicators are meant to provide “an objective standard for the optimal quality of the care process” and caters specifically to vulnerable elders (33). They are a set of process quality indicators that evaluate care processes for general medical conditions and geriatric prevalent conditions (33). Three papers by Arora et al. used a set of 16 Quality Indicators (QIs) selected from the original list, and the selected QIs were in general hospital care and geriatric prevalent conditions (26, 33, 41). These were selected by a team of geriatricians and hospitalists based on the ease of operationalizing them into medical chart review and their applicability to the general medicine inpatient service (33). Arora et al. noted that it was important to include geriatric-prevalent condition specific quality measures for elderly inpatients, and not just general medical conditions (33). This is especially as geriatric-specific indicators tend to have a lower adherence level than those regarding general hospital care, especially higher order skills involving evaluation and treatment (26, 33). It was noted that adherence for screening for cognitive function was low (26). The studies used a composite quality score to assess adherence to the QIs, based on the proportion of QIs triggered that were met (41).

The resident-sensitive quality measures that are “meaningful in patient care and most likely attributable to resident care”, although performance may still be affected by team effects (19). The set of RSQMs for general internal medicine (GIM) care were developed through consensus methods (19). They covered specific clinical conditions as well as general care in the GIM ward (19). There were 89 measures in total, relating to documentation and orders, and including those specific to their primary diagnosis and those applicable to chronic comorbidities (19). However, it is possible that resident contribution is greater in and more valid to be assessed for chronic comorbidities (19).

##### 3.3.2.2 Advantages of process of care measures

Specific process of care measures do not require adjustment based on disease severity, unlike outcome measures (33). Focusing on discrete tasks rather than broader composite measures allows them to provide more specific feedback to guide provider behavioural changes and program or learner evaluation, valuable for organisational and individual efforts (19). It also allows flexibility in combining measures into various composite measures, such as composite measures for processes across conditions (discharge, medication ordering etc) or condition-specific composite measures (19). Composite quality measures have been suggested as important to cater to the multiple care processes for complex patients (33). Additionally, process of care measures are objective. Using RSQMs instead of raters to observe resident performance reduces rater bias (19). Process measure data like documentation and orders may also be more easily accessed, through the electronic health record (EHR) (19). Process measures may also be easier to operationalise (19). They may also be more attributable to individual physicians while outcome measures result from a system of multiple factors (19). While the issues of measurement and attribution are being tackled for outcome measures, it has been suggested that process measures can be used as a “starting point” (19).

##### 3.3.2.3 Disadvantages of process of care measures

The true level of care provided may not be accurately captured in written medical records and measures that rely on this documentation may underestimate the care provided (26). However, standards of documentation and process of care have been shown to be correlated, as proper documentation is crucial for communication within the care team (26). Hence, if the written records do not properly document the level of care, the processes of care executed may have been sub-optimal as well. Training and evaluation of documentation accuracy is therefore important for the process measures to be accurate and to aid communication in the team.

However, quality of care may also be overestimated from measures that rely on chart documentation of the screening process but do not assess accuracy or assess follow-ups to provide the care indicated from the results of the screening (33). Adherence to screening processes does not necessarily translate to diagnosis, treatment and documentation and staff should be trained in performing accurate assessments and follow-through (33). Screening process related hospital care indicators may also detect standard nursing or protocol-driven care, leading to an overestimation of the adherence levels (33). Hence, quality measurement based on processes of care should focus more on diagnosis and treatment (33).

The process measures may also lack association with certain patient outcomes but are a more direct measurement of quality of care. As earlier discussed regarding the significance of outcome measures, outcome measures are not necessarily reflective of quality of care, which was in turn directly assessed using process measures in studies by Arora et al. and Scott et al. (19, 26, 33, 41). Although some have suggested that performance on process measures could translate to better outcomes, this is not necessarily the case (19). The association is not always clear and the factors involved may be hard to measure (19). Given that the relation between quality of care and outcome measures are usually complicated by other factors, process measures are valuable as direct assessments of quality of care. It would be ideal if this does lead to improved patient outcomes, given the cost of measuring and improving quality of care (33).

In a study by Scott et al., the reviewers used the “eyeball” test to identify the quality of care factors, where only processes that were evident from hospital chart clinical documentation were included (30).

#### 3.3.3 Performance management

With the rise of pay-for-performance and public reporting programs, there is increasing focus on measuring and rewarding adherence to measures of quality of care. It is under this backdrop that developing valid measures of quality of care has drawn increasing research attention (33).

##### 3.3.3.1 Heterogeneity in IM wards

A challenge raised in conducting performance management was the heterogeneity of IM wards. In a study by Yousefi et al. the IM provider group consisted of physicians of multiple subspecialties, which was important to note if interpreting comparisons with more homogenous groups (37). Verma et al. also reported that the general medicine patient population has significant heterogeneity in their characteristics, conditions responsible for admission, resource use and outcomes (35). This makes averages and other such common methods of processing outcome measures less suitable. Further research into this complex patient population and the reasons for the variability are important to improve quality of care (35). He noted the significant potential in developing measures of quality of care for this patient population and in studying the variations in care delivered and accompanying outcomes and improving quality of care (35). Multicentre research supported by electronic data collection and linkage was suggested as future work (35).

##### 3.3.3.2 Comparison across hospitals of different complexity

A study found “homogeneous quality of care of [Internal Medicine Units] along different complexity of hospitals” which was suggested to be partially due to the long residence program to specialize in IM in Spain, and less dependence on complex technology compared to other specialties (29).

One study noted that university hospital internal medicine wards were mostly sub-specialised, with programmed admissions not from the emergency room. Hence, they were excluded as benchmarking would have been inconsistent (24).

##### 3.3.3.3 Physician-level performance management

Initiatives that target practice patterns at the level of individual physicians (e.g., physician level pay-for-performance, reporting resource use comparisons) on top of the more common hospital level programs like hospital value-based purchasing and penalties for 30-day readmissions will be important to increase the effectiveness of value-based healthcare measures (23). Hospital level performance management programs assume that they will be able to ultimately affect behaviour at the physician level (23). However, it will be valuable to also introduce initiatives like physician level pay-for-performance programs and reporting resource utilisation comparisons among physicians in the same hospital (23). Measuring and providing physician level feedback could help to more effectively increase the value of care (23). Physician level performance management is a valuable direction to explore especially as the large variation in physician level spending within hospitals was found to not impact 30-day mortality and readmissions (23). This suggests that higher-spending physicians could possibly reduce resource utilisation while maintaining patient outcomes (23). Meanwhile, spending across hospitals did not vary as much (23). Hence, it would be valuable to target physician level spending as part of performance management programs.

##### 3.3.3.4 Provider and patient segments

It was found that there was no significant difference in outcomes of General IM patients whether the care was provided by a teaching or non-teaching service (44). Hence having teaching units is not a cause of concern for performance management and financial penalties (44). There would also likely not be a need to distinguish them in managing performance.

Similarly, various clinical and cost outcomes were found to be similar between hospitalist, APC, and resident teams although there was some variation in consultant involvement and discharge time (18). This allowed them to conclude that the team structure does not have a significant impact on clinical outcomes (18). Another study also found outcomes and efficiency to be similar when comparing a physician assistant/hospitalist service with traditional house staff services (36). The study by Yousefi et al. found hospitalist care to be associated with different outcomes than IM providers, but these were no longer present consistently when patients with specific diagnoses were compared (37). Patients admitted by IM providers tend to have cardiac condition diagnoses and may have higher acuity levels, spending more time in specialised units (37). The change in association levels when analysing within diagnoses was also attributed partially to the relation between caseload and outcomes (37). Higher readmission risk only remained for pneumonia hospitalisations (37). Hence, there would likely not be a need to distinguish between these teams in setting benchmarks for performance management, but the necessary adjustments need to be made.

However, it was found that care by locum tenens internists resulted in slightly higher Medicare Part B charges, increased LOS and lower 30-day readmission rates while 30-day mortality rates remained the same (17). This might have bearing on the performance management of these physician groups.

Although post-discharge outcomes are similar between weekend and weekday discharges, weekend discharges had a shorter LOS and tended to be lower risk patients (38). The possible implications of this on performance management should be noted.

##### 3.3.3.5 Documentation training

There is value in educating hospitalists on the importance of comprehensive documentation of comorbidities (25). Such documentation is necessary to accurately capture the acuity level of the patients which affects physician level measures (e.g., mortality scores) that may be linked to reimbursement (25). With the transition of Diagnosis Related Groups (DRGs) to Medicare Severity-Diagnosis Related Groups (MS-DRGs), higher reimbursement is given based on documented complications or comorbidities (25). Hence, focusing on clinical documentation affects perceived quality of care and is important for maximising reimbursement at the hospital level (25). Constant education and re-evaluation of “high yield” comorbidities (those that contribute significantly to expected LOS and expected mortality) will be important (25). Sparks et al. presented a simple intervention to improve such documentation, that had advantages over clinical documentation improvement programs (25).

##### 3.3.3.6 Nursing related performance management

Nursing care should also be included in performance management to improve quality of care for geriatric patients, given the significance of nursing care for this inpatient group (26). Performance measurements for “proactive patients sharing” was also noted as important for physician-nurse communication in making care plans (3).

##### 3.3.3.7 Other insights

Clever et al. presented a method of constructing Instrumental Variables for investigating the association between physician characteristics and outcomes where ratings from individual patients could be confounded by patient-level factors (40). The method was to use the average ratings of the physician characteristic provided by other patients (40). The authors used it to investigate the association between physician communication behaviours and patient satisfaction (40). Arora et al. noted the difficulty of “assessing process-outcome causal relationships in observational study designs” and similarly considered an instrumental variable approach but “failed to identify a potential variable that would relate to whether patients would receive certain quality indicators” (33).

## 4 Discussion

### 4.1 Available evidence, evidence gaps and future directions

#### 4.1.1 Lack of available evidence

We were unable to find evidence with the most relevant angle of addressing how to conduct performance management of generalist inpatient care. There was also no evidence reporting on a specific implementation of performance management of generalist care, such as with reimbursement elements. There was limited evidence that took the directly relevant angle of addressing how to measure the quality of generalist inpatient care, or the patient-related outcomes to monitor. Most took the indirect approach of assessing a certain implementation of generalist care, from which the measures used for the evaluation can be obtained. However, these are less likely to form a comprehensive list of the measures to use in evaluating quality, as the measures selected would depend heavily on the aim and scope of the paper. A clear focus on value-based care was also largely missing. However, there was reference to performance management in some of the papers (Supplementary Appendix 5). This suggests that there is significant room to explore in more detail performance management methods for inpatient generalist care, including the measures used and operationalising them for performance management through linking them to reimbursement. Of the available evidence, many were concerning the elderly inpatient population, who are likely to have comorbidities, rather than directly focusing on patients with multimorbidity. The quality measures used in such studies likely apply to patients with multimorbidity, but those specific to geriatric needs would not.

#### 4.1.2 Common clinical outcome measures as the primary indicators

Outcome measures were most commonly used rather than process measures. The outcome measures were mostly common generic measures of clinical outcomes (e.g., LOS, readmission rate) which were used in majority of the papers (Table 2). There were also patient or carer reported outcomes. The specific permutations used varied, such as the duration post-discharge or the instrument. The frequency of usage of such non-clinical measures was low, especially at the level of specific measures. Caregiver burden and staff related measures also had disproportionately low frequencies. Hence, in evaluation of generalist care, there seems to be a lack of emphasis on patient reported and caregiver or staff related outcome measures which shows an inadequate response to the increasing recognition of their importance. The traditional focus on clinical factors still seems to be the default evaluation method. The bias towards clinical outcome measures as opposed to reported measures could also be due to the greater ease in obtaining the data for such measures. It could also be due to the inaccuracies of reported outcome measures, such as recall bias (34). These may make it unwise to utilise only these reported outcome measures, but reported measures should be used in addition to clinical measures for a more comprehensive evaluation. Multiple outcome measures should be used especially considering that they may not be individually representative of the quality of care. They may each interact differently with various other factors, and associate with quality of care to different extents.

#### 4.1.3 Relevance of process of care measures

Although process measures were reported less frequently, both papers which took a direct approach in addressing how to evaluate the quality of generalist inpatient care exclusively used specific process measures (19, 26). Another paper that aimed to assess how the quality of care is associated with unplanned readmissions also curated a list of process measures (30). Hence, it appears that process measures are more suited as direct measurements of quality of care. The advantages and value of process measures have been clearly stated (19, 33). It is notable that in aiming to select resident-sensitive quality measures for general internal medicine, Kinnear et al. obtained only process measures as the final set of measures after a consensus methodology (19). Possible explanations include bias towards process measures that are more accessible, and that outcome measures result from team efforts and system and patient factors, although there has been some evidence that residents contribute significantly to outcomes (19). Nonetheless, this may imply that process measures are significant in evaluating quality of care that is attributable specifically to individual physicians as opposed to outcome measures that are more reflective of the efforts of the care team (19).

It appears that the process measures were reported less frequently because only a few of the papers wanted to directly measure quality of care. The sets of specific process indicators were only used for the purpose of being able to directly measure quality of care, whether with that as the aim or to ultimately explore the association with certain outcome measures. Evaluating the care service did not appear to be the primary focus for most of these papers. For Kinnear et al. developing the RSQMs was the aim (19). Only one of the studies had it as more of a focus (26). The 3 studies aiming to assess the association of quality of care with outcome measures like readmissions, mortality and functional decline (30, 33, 41), is a reminder of how the relation of quality of care and patient outcomes is not straightforward. Most of the rest of the papers instead focused on just outcome measures to evaluate the care service or provider. Since better quality of care does not necessitate better outcomes, the decision to focus on outcomes rather than quality of care is reasonable and that appears to be the preferred method of evaluating care interventions.

Although many of the process measures were applied to the context of elderly patients, some of the measures from the papers were not specific to geriatric conditions and could potentially be applied to non-geriatric patients. For example, the discharge related process of arranging appropriate medical follow up for patients who require at least 1 review visit (30). Hence, further research could assess the potential of including these measures in assessing care processes for patients in generalist care wards.

The process measures ranged across all the WHO healthcare quality dimensions, except for equity (Table 3). It may be valuable to identify process measures relevant to this dimension especially for contexts where equity may be an issue. It may also be valuable to explore more process measures for effectiveness, efficiency and patient-centredness which had few measures. However, measures across these dimensions are highly related and classification may be arbitrary. Overall, discharge planning and needs assessment were areas commonly addressed by the process measures (Table 3). This is not surprising given that continuity of care and discharge are recognised as common issues in care for elderly patients (34) and studies concerning elderly patients formed the bulk of studies that contributed process measures. Similarly, the elderly often have more complex needs, ranging from physical and mental to social needs (47).

### 4.2 Recommendations for practice

#### 4.2.1 Including clinical and patient-reported outcome measures

The general clinical measures of LOS, 30-day readmission rate and inpatient mortality rate that are commonly used in performance management of specialist conditions should be retained for generalist care as they are still highly relevant as quality indicators (Table 2). However, others like 30-day complication rate, return to operating theatre rate and blood transfusion rate monitored in surgeries such as under Singapore's VDC program [Unpublished source: MOH Value Driven Care (VDC) Program, 2021] are less relevant for generalist care (Table 2). Instead, patient reported measures should be included and Quality of Life (QoL), ADL and patient satisfaction in particular were more frequently used (Table 2). Care should be taken to include these where possible as they tend to be neglected in comparison to clinical measures. This will be in alignment with the recognition of the value of patient reported measures in performance management, seen in plans to include PROMs in Singapore's VDC program for specialist care (Unpublished source: MOH CQPV, 2017).

The patient outcomes can also be measured at different time frames. Longer term measures like 30-day mortality instead of just inpatient mortality could be beneficial to include and this variation was used quite frequently (Supplementary Appendix 6). Together with cost data which is fairly frequently collected (Table 2), these measures will allow for a comprehensive evaluation of the value delivered. The impact on caregivers and staff can also be measured and improved as part of holistic performance management especially as they can ultimately impact patients (22), although such measures not as directly relevant for monitoring patient outcomes. A comprehensive range of patient-related outcomes across various spheres of impact should be used, and the most suitable selected (22).

#### 4.2.2 Relevance of process of care measures

The importance of monitoring both patient outcomes and process of care outcomes in pay-for-performance programs has been recognised (48). The process of care outcomes of the RSQMs developed and the selected ACOVE measures can also be used for performance management of generalist care. Process measures could seem particularly relevant for elderly patients given that most of the papers that mentioned process measures were focused on this group, although it should be noted that 3 of the 6 were by the same author using the same set of measures. Nonetheless, process measures for elderly patients could use further research and validation to strive to include them in performance management, especially given the low-adherence of geriatric-specific measures (33). The RSQMs can be used for general IM patients, while they could both be valuable for assessing the care of vulnerable elders as they do not have much overlap which is likely at least partially due to the fact that RSQMs are resident-sensitive and not elder-specific unlike ACOVE. However, the value and appropriateness of using them together for vulnerable elders should be further assessed. To evaluate quality of care for vulnerable elders in general, the ACOVE measures are likely suitable to be used at minimum. Although the RSQMs specifically measure the quality of care by residents, it is possible that they could contribute to an evaluation of overall quality of care. If used in such a manner for vulnerable elders, it is important that the geriatric-prevalent conditions from ACOVE are included as well. This is important as geriatric condition-specific measures tend to have lower adherence (33).

#### 4.2.3 Next steps: applying methods and validating measures

Focusing on process measures has been suggested as a “starting point” before progressing to identifying and validating outcome measures for care evaluation (19). In general, in order to apply the RSQMs and ACOVE measures for performance management in any contexts beyond the specific settings of the studies, it will be necessary to further research and validate the indicators appropriate for specific local contexts. The studies conducted thus far will be valuable in guiding this process. For example, the work by Kinnear et al. validates the consensus-method process of selecting resident-sensitive quality measures which they suggested that institutions use to develop quality measures for conditions important in their local contexts (19). They emphasised that the RSQMs need to be developed and tested in other clinical environments (19). Similarly for patient-outcome measures, the “best-suited” should be selected (22). More research including RCTs to investigate the relationship between quality of care and outcome measures could also be valuable to guide selection of the measures. However, even if they cannot be determined to be causal, association already provides impetus to improve adherence to those process quality of care measures (41). Association could support the usage of the process measure, and also support the usage of the outcome measure.

The next step would be to operationalise the measures for performance management. For process measures, this could include determining the target process adherence levels and how they can be linked to reimbursement. It has been suggested that compliance targets should be less than 100% to allow for valid cases (19). Specific operational definitions of the process measures will be needed to assess if they can be accurately measured from EHR data (19). Testing the feasibility and validating the operationalisation of the measures would also be important (19). Kinnear et al. noted that once the RSQMs are identified and have operational definitions, extracting and displaying the data can rely on automation, to allow for sustained use of the measures with less resources (19). Given the current lack of research that is directly about performance management of generalist care, research that operationalises quality indicator sets for performance management in the local context will be highly valuable. The validated parameters could be used for performance management across the public healthcare institutions through creation of a dashboard. Deficiencies in adherence to process measures especially can guide areas where more training is required. For instance, the poor adherence to geriatric-specific QIs points to the need for better training in caring for elderly patients (26). Sharing of best-practices can help standardise care processes that could decrease costs and improve patient outcomes (32). In the long term, the performance management of generalist care could promote improvements in care delivery and greater value through such sharing of best-practices.

#### 4.2.4 Tackling issues and other initiatives

To reduce the impact of the heterogeneity on performance management, cross-diagnosis characteristics like acuity level could be used to further group the patients into more homogeneous groups. For example, the sub-groups of patients that are selected for integrated generalist care programs in various hospitals may have similar levels of less critical generalist needs, and the process of care and patient outcome measures could potentially be benchmarked. Examples of these programs would be the Integrated General Hospital at Alexandra Hospital (3) and Integrated General Medicine at Singapore General Hospital in Singapore (7).

Other initiatives for value-based performance management for generalist care should also be implemented, including greater focus on physician level spending. Performance management initiative targeted at physician level spending could more effectively increase the value of care (23). Physician level performance management can use or adapt measures like the RSQMs and their development method. Other initiatives like documentation education and evaluation for care processes and comorbidities are also important for more accurate and effective usage of performance management measures (25, 26).

#### 4.2.5 Definition of value

Value of healthcare has been measured using efficiency which can be calculated from the health outcomes and the cost (49). In papers that evaluated cost of care, various costs were used, often the physician spending for that hospitalisation episode (Supplementary Appendices 4, 6). However, for generalist care of multimorbid patients, it can be complicated to calculate these costs and standardise them for benchmarking across institutions and even countries. In contrast, measures like LOS and readmission rate which have been frequently used for evaluations are more clearly defined and lend themselves to comparisons more easily. While many papers seemed to use LOS and readmission rate as patient outcome measures, there were also others that used LOS especially, as a measure of resource utilisation (Supplementary Appendix 6). Hence, it may be useful to consider an alternative method of evaluating value, where resource utilisation is used as a surrogate for cost, where resource utilisation could be measured using LOS. The outcome measures for evaluating the health outcomes could then consist of the other patient outcomes and process measures. Further research on this could be valuable.

### 4.3 Limitations

A limitation was the lack of a registered protocol. Another limitation was the scope of publications reviewed. Grey literature was excluded and references of the publications were not hand searched. In addition, the initial domains of value-based care and multimorbidity used in the search may have excluded publications which would have been similar in eligibility to the publications that were included in the review. For example, we may have excluded a study set in an internal medicine ward and hence considered as providing generalist care, although like other papers included the review, the element of value-based care may not have been eventually present or it may not have been specifically for patients with multimorbidity. This should be noted if a systematic review is conducted on this area to obtain detailed recommendations for evaluating the quality of generalist care and conducting performance management. In processing the measures from the papers, other ways of categorising and analysing the findings were not explored, such as the level of hospital care (acute or sub-acute). The quality indicators relevant may be highly specific to the setting including level of care. Insights on cost measurement was not a focus of the paper although data was charted and presented in Supplementary Appendices 4–6. The analysis of measures did not take note of the combinations that measures were used by papers, although the data was charted. This could be valuable future work. The data charting did not differentiate between primary and secondary outcomes.

## 5 Conclusion

Research directly concerning how to conduct performance management for value-based generalist care in the inpatient setting is scarce and further research is needed. However, from the research available on specific elements like measuring quality of care and evaluating specific implementations of generalist care, we made recommendations on measures that can be used based on frequency of usage and types of measures. It was found that LOS, readmission rate and mortality are most commonly used. Process measures are also important, such as selected ACOVE measures and RSQMs. We also suggested initiatives to support performance management of such care including documentation training. These guidelines would be valuable for the development of the performance management methods for generalist inpatient care in Singapore, and the next steps would include further contextualization of the measures and initiatives to the local hospital settings, validation of these measures and operationalising them for performance management.

## Data Availability

The original contributions presented in the study are included in the article/**Supplementary Material**, further inquiries can be directed to the corresponding author.
