# Supplementary material for: Performance management of generalist care for hospitalised multimorbid patients—a scoping review for value-based care
Source: Front Health Serv. 2024 Feb 26;3:1147565. doi: 10.3389/frhs.2023.1147565 (PMC10925702; doi:10.3389/frhs.2023.1147565)
Supplement: Supplementary file 1 [file Datasheet1.pdf]

Supplementary Material

Performance Management for Generalist Care of Hospitalised Multimorbid Patients – A Scoping Review of Value-Based Care

Jia En Joy Khoo*, Cher Wee Lim, Yi Feng Lai

*** Correspondence:** Jia En Joy Khoo: joy_khoo@u.nus.edu

Appendix 1 Preferred Reporting Items for Systematic reviews and Meta-Analyses extension for Scoping Reviews (PRISMA-ScR) Checklist

Template obtained from PRISMA. Prisma-statement.org. http://www.prisma-statement.org/Extensions/ScopingReviews (accessed 23 Jan 2022).

SECTION	ITEM	PRISMA-ScR CHECKLIST ITEM	REPORTED ON PAGE #
**TITLE**			
Title	1	Identify the report as a scoping review.	1
**ABSTRACT**			
Structured summary	2	Provide a structured summary that includes (as applicable): background, objectives, eligibility criteria, sources of evidence, charting methods, results, and conclusions that relate to the review questions and objectives.	1-2
**INTRODUCTION**			
Rationale	3	Describe the rationale for the review in the context of what is already known. Explain why the review questions/objectives lend themselves to a scoping review approach.	2-3
Objectives	4	Provide an explicit statement of the questions and objectives being addressed with reference to their key elements (e.g., population or participants, concepts, and context) or other relevant key elements used to conceptualize the review questions and/or objectives.	2-3
**METHODS**			
Protocol and registration	5	Indicate whether a review protocol exists; state if and where it can be accessed (e.g., a Web address); and if available, provide registration information, including the registration number.	3
Eligibility criteria	6	Specify characteristics of the sources of evidence used as eligibility criteria (e.g., years considered, language, and publication status), and provide a rationale.	3
Information sources*	7	Describe all information sources in the search (e.g., databases with dates of coverage and contact with authors to identify additional sources), as well as the date the most recent search was executed.	3
Search	8	Present the full electronic search strategy for at least 1 database, including any limits used, such that it could be repeated.	Supplementary Material Page 4-12
Selection of sources of evidence†	9	State the process for selecting sources of evidence (i.e., screening and eligibility) included in the scoping review.	3
Data charting process‡	10	Describe the methods of charting data from the included sources of evidence (e.g., calibrated forms or forms that have been tested by the team before their use, and whether data charting was done independently or in duplicate) and any processes for obtaining and confirming data from investigators.	3
Data items	11	List and define all variables for which data were sought and any assumptions and simplifications made.	3 and Supplementary Material Page 19-38
Critical appraisal of individual sources of evidence§	12	If done, provide a rationale for conducting a critical appraisal of included sources of evidence; describe the methods used and how this information was used in any data synthesis (if appropriate).	Not completed

SECTION	ITEM	PRISMA-ScR CHECKLIST ITEM	REPORTED ON PAGE #
Synthesis of results	13	Describe the methods of handling and summarizing the data that were charted.	3
**RESULTS**			
Selection of sources of evidence	14	Give numbers of sources of evidence screened, assessed for eligibility, and included in the review, with reasons for exclusions at each stage, ideally using a flow diagram.	4
Characteristics of sources of evidence	15	For each source of evidence, present characteristics for which data were charted and provide the citations.	5-6 (citations in References), Supplementary Material Page 13-18
Critical appraisal within sources of evidence	16	If done, present data on critical appraisal of included sources of evidence (see item 12).	Not completed
Results of individual sources of evidence	17	For each included source of evidence, present the relevant data that were charted that relate to the review questions and objectives.	Supplementary Material Page 19-49
Synthesis of results	18	Summarize and/or present the charting results as they relate to the review questions and objectives.	4-13, Supplementary Material Page 50-62
**DISCUSSION**			
Summary of evidence	19	Summarize the main results (including an overview of concepts, themes, and types of evidence available), link to the review questions and objectives, and consider the relevance to key groups.	13-16
Limitations	20	Discuss the limitations of the scoping review process.	15-16
Conclusions	21	Provide a general interpretation of the results with respect to the review questions and objectives, as well as potential implications and/or next steps.	16
**FUNDING**			
Funding	22	Describe sources of funding for the included sources of evidence, as well as sources of funding for the scoping review. Describe the role of the funders of the scoping review.	16

JBI = Joanna Briggs Institute; PRISMA-ScR = Preferred Reporting Items for Systematic reviews and Meta-Analyses extension for Scoping Reviews.
* Where *sources of evidence* (see second footnote) are compiled from, such as bibliographic databases, social media platforms, and Web sites.
† A more inclusive/heterogeneous term used to account for the different types of evidence or data sources (e.g., quantitative and/or qualitative research, expert opinion, and policy documents) that may be eligible in a scoping review as opposed to only studies. This is not to be confused with *information sources* (see first footnote).
‡ The frameworks by Arksey and O'Malley (6) and Levac and colleagues (7) and the JBI guidance (4, 5) refer to the process of data extraction in a scoping review as data charting.
§ The process of systematically examining research evidence to assess its validity, results, and relevance before using it to inform a decision. This term is used for items 12 and 19 instead of "risk of bias" (which is more applicable to systematic reviews of interventions) to include and acknowledge the various sources of evidence that may be used in a scoping review (e.g., quantitative and/or qualitative research, expert opinion, and policy document).

From: Tricco AC, Lillie E, Zarin W, O'Brien KK, Colquhoun H, Levac D, et al. PRISMA Extension for Scoping Reviews (PRISMAScR): Checklist and Explanation. Ann Intern Med. 2018;169:467–473. doi: 10.7326/M18-0850.

Appendix 2 Search Strategies

MEDLINE Search Strategy

The searches for the four domains were joined by “AND” to produce the final set of search results. Filter: from 2006/1/1 - 3000/12/12

Concept Domain	Search Terms
Multimorbidity	"multimorbidity" [tiab] OR "multi-morbidity" [tiab] OR "multimorbidities" [tiab] OR "multi-morbidities" [tiab] OR "comorbidity" [tiab] OR "co-morbidity" [tiab] OR "comorbidities" [tiab] OR "co-morbidities" [tiab] OR "chronic condition*" [tiab] OR "chronic issue*" [tiab] OR "chronic clinical condition*" [tiab] OR "chronic medical condition*" [tiab] OR "chronic medical issue*" [tiab] OR "chronic health condition*" [tiab] OR "chronic health issue*" [tiab] OR "chronic illness*" [tiab] OR "chronic medical illness*" [tiab] OR "chronic disease*" [tiab] OR "chronic medical disease*" [tiab] OR "chronic clinical disease*" [tiab] OR "multiple condition*" [tiab] OR "multiple issue*" [tiab] OR "multiple clinical condition*" [tiab] OR "multiple clinical issue*" [tiab] OR "multiple medical condition*" [tiab] OR "multiple medical issue*" [tiab] OR "multiple health condition*" [tiab] OR "multiple health issue*" [tiab] OR "multiple illness*" [tiab] OR "multiple disease*" [tiab] OR "multiple medical illness*" [tiab] OR "multiple clinical disease*" [tiab] OR "multiple medical disease*" [tiab] OR "concomitant condition*" [tiab] OR "concomitant issue*" [tiab] OR "concomitant clinical condition*" [tiab] OR "concomitant medical condition*" [tiab] OR "concomitant medical issue*" [tiab] OR "concomitant health condition*" [tiab] OR "concomitant health issue*" [tiab] OR "concomitant illness*" [tiab] OR "concomitant disease*" [tiab] OR "concomitant medical illness*" [tiab] OR "concomitant clinical disease*" [tiab] OR "concomitant medical disease*" [tiab] OR "concurrent condition*" [tiab] OR "concurrent issue*" [tiab] OR "concurrent clinical condition*" [tiab] OR "concurrent medical condition*" [tiab] OR "concurrent medical issue*" [tiab] OR "concurrent health condition*" [tiab] OR "concurrent health issue*" [tiab] OR "concurrent illness*" [tiab] OR "concurrent disease*" [tiab] OR "concurrent medical illness*" [tiab] OR "concurrent medical disease*" [tiab] OR "co-existing condition*" [tiab] OR "coexisting condition*"[tiab] OR "coexisting medical condition*"[tiab] OR "co-existing medical condition*" [tiab] OR "coexisting health condition*"[tiab] OR "co-existing health condition*" [tiab] OR "coexisting illness*"[tiab] OR "co-existing illness*" [tiab] OR "coexisting disease*"[tiab] OR "co-existing disease*" [tiab] OR "coexisting medical illness*"[tiab] OR "co-existing medical illness*" [tiab] OR "multimorbidity"[MeSH Terms] OR "multiple chronic conditions"[MeSH Terms] OR "chronic healthcare condition*"[tiab] OR "coexisting issue*"[tiab]
Generalist-led Care	"Family medicine" [tiab] OR "internal medicine" [tiab] OR "internist*" [tiab] OR "geriatric*" [tiab] OR "gerontology" [tiab] OR "generalist*" [tiab] OR "general medicine" [tiab] OR "general practice" [tiab] OR "family physician*" [tiab] OR "care team*" [tiab] OR "healthcare team*"[tiab] OR "health team*" [tiab] OR "interdisciplinary" [tiab] OR "multidisciplinary" [tiab] OR "general practitioner*" [tiab] OR "general care" [tiab] OR "generalized care"[tiab] OR "integrated care"[tiab] OR "interprofessional"[tiab] OR "team-based"[tiab] OR "physicians, family"[MeSH Terms] OR "family practice"[MeSH Terms] OR "geriatrics"[MeSH Terms] OR "General practice"[MeSH Terms] OR "patient care team"[MeSH Terms] OR "general practitioners"[MeSH Terms] OR "health services for the aged"[MeSH Terms] OR "geriatric assessment"[MeSH Terms]
Performance Management	"perform*" [tiab] OR "outcome*" [tiab] OR "output*" [tiab] OR "out put*"[tiab] OR "management" [tiab] OR "review*" [tiab] OR "evaluation*" [tiab] OR "rating*" [tiab] OR "rank*" [tiab] OR "dialogue*" [tiab] OR "feedback*" [tiab] OR "result*" [tiab] OR "measure*" [tiab] OR "metric*" [tiab] OR "goal*" [tiab] OR "objective*" [tiab] OR "target*" [tiab] OR "benchmark*" [tiab] OR "benchlearn*" [tiab] OR "criteria" [tiab] OR "criterion" [tiab] OR "standard*" [tiab] OR "indicator*" [tiab] OR "score*" [tiab] OR "scoring" [tiab] OR "competence*" [tiab] OR "grading" [tiab] OR "grade*" [tiab] OR "assess*" [tiab] OR "rate*" [tiab] OR "monitor*" [tiab] OR "audit*" [tiab] OR "report*" [tiab] OR "recommend*" [tiab] OR "guide*" [tiab] OR "guiding" [tiab]

	OR "protocol*" [tiab] OR "measurable*" [tiab] OR "quality" [tiab] OR "success*" [tiab] OR "practice*" [tiab] OR "effective*" [tiab] OR "compliance" [tiab] OR "comply*"[tiab] OR "adher*" [tiab] OR "six sigma" [tiab] OR "Donabedian" [tiab] OR "critiqu*" [tiab] OR "appraisal*" [tiab] OR "accountab*"[tiab] OR "quality indicators, health care"[MeSH Terms] OR "standard of care"[MeSH Terms] OR "health care evaluation mechanisms"[MeSH Terms] OR "quality assurance, health care"[MeSH Terms] OR "clinical competence"[MeSH Terms] OR "practice patterns, physicians'"[MeSH Terms] OR "professional practice gaps"[MeSH Terms] OR "health care surveys"[MeSH Terms] OR "comparative effectiveness research"[MeSH Terms] OR "employee performance appraisal"[MeSH Terms] OR "management quality circles"[MeSH Terms] OR "guideline adherence"[MeSH Terms]
Value-Based Care	"health outcome*" [tiab] OR "health care outcome*" [tiab] OR "healthcare outcome*" [tiab] OR "healthcare delivery outcome*" [tiab] OR "care delivery outcome*" [tiab] OR "care outcome*" [tiab] OR "intervention outcome*" [tiab] OR "outcome improvement*" [tiab] OR "better outcome*" [tiab] OR "improved outcome*" [tiab] OR "improving outcome*" [tiab] OR "health improvement*" [tiab] OR "improved health" [tiab] OR "improving health" [tiab] OR "better health" [tiab] OR "value*" [tiab] OR "result focused" [tiab] OR "results focused" [tiab] OR "result driven" [tiab] OR "results driven" [tiab] OR "result based" [tiab] OR "results based" [tiab] OR "merit increase*" [tiab] OR "merit raise*" [tiab] OR "performance based" [tiab] OR "performance centric" [tiab] OR "performance centered" [tiab] OR "performance driven" [tiab] OR "performance focused" [tiab] OR "performance centred" [tiab] OR "outcome based" [tiab] OR "outcome centered" [tiab] OR "outcome centred" [tiab] OR "outcomes centered" [tiab] OR "outcomes based" [tiab] OR "outcome centric" [tiab] OR "outcome driven" [tiab] OR "outcomes driven" [tiab] OR "outcomes focussed" [tiab] OR "outcomes focused" [tiab] OR "outcome focussed" [tiab] OR "outcome focused" [tiab] OR "bundled payment*" [tiab] OR "pay for performance" [tiab] OR "payment for performance" [tiab] OR "pay" [tiab] OR "p4p" [tiab] OR "payment*" [tiab] OR "reward*" [tiab] OR "bonus*" [tiab] OR "incentive*" [tiab] OR "contract" [tiab] OR "contracts" [tiab] OR "non-payment" [tiab] OR "nonpayment" [tiab] OR "financ*" [tiab] OR "monetary" [tiab] OR "paid" [tiab] OR "paying" [tiab] OR "penalt*" [tiab] OR "money" [tiab] OR "cash" [tiab] OR "fund*" [tiab] OR "fee" [tiab] OR "fees" [tiab] OR "econom*" [tiab] OR "disburs*" [tiab] OR "renumerat*" [tiab] OR "reimburse*" [tiab] OR "compensat*" [tiab] OR "incentive*" [tiab] OR "function centric" [tiab] OR "function based" [tiab] OR "function driven" [tiab] OR "function centered" [tiab] OR "function centred" [tiab] OR "quality of care" [tiab] OR "quality of health care" [tiab] OR "quality of healthcare" [tiab] OR "quality based" [tiab] OR "cost effective*" [tiab] OR "costs reduction*" [tiab] OR "cost reduction*" [tiab] OR "reduced cost*" [tiab] OR "lowered cost*" [tiab] OR "low cost*" [tiab] OR "lowest cost*" [tiab] OR "reasonable cost*" [tiab] OR "minimized cost*" [tiab] OR "cost minimization*" [tiab] OR "cost minimisation*"[tiab] OR "costs minimization*" [tiab] OR "cost improvement*" [tiab] OR "improved cost*" [tiab] OR "reimbursement, incentive"[MeSH Terms] OR "physician incentive plans"[MeSH Terms] OR "employee incentive plans"[MeSH Terms] OR "health care reform"[MeSH Terms] OR "public health systems research"[MeSH Terms] OR "value-based health insurance"[MeSH Terms] OR "relative value scales"[MeSH Terms] OR "value-based purchasing"[MeSH Terms] OR "salaries and fringe benefits"[MeSH Terms]

CINAHL Search Strategy

The searches for the four domains were joined by “AND” to produce the final set of search results. Limiter: Published Date 20060101 - 20211231

Concept Domain	Search Terms
Multimorbidity	TI("multimorbidity" OR "multi-morbidity" OR "multimorbidities" OR "multi-morbidities" OR "comorbidity" OR "co-morbidity" OR "comorbidities" OR "co-morbidities" OR "chronic condition*" OR "chronic issue*" OR "chronic clinical condition*" OR "chronic medical condition*" OR "chronic medical issue*" OR "chronic health condition*" OR "chronic health issue*" OR "chronic illness*" OR "chronic medical illness*" OR "chronic disease*" OR "chronic medical disease*" OR "chronic clinical disease*" OR "multiple condition*" OR "multiple issue*" OR "multiple clinical condition*" OR "multiple clinical issue*" OR "multiple medical condition*" OR "multiple medical issue*" OR "multiple health condition*" OR "multiple health issue*" OR "multiple illness*" OR "multiple disease*" OR "multiple medical illness*" OR "multiple clinical disease*" OR "multiple medical disease*" OR "concomitant condition*" OR "concomitant issue*" OR "concomitant clinical condition*" OR "concomitant medical condition*" OR "concomitant medical issue*" OR "concomitant health condition*" OR "concomitant health issue*" OR "concomitant illness*" OR "concomitant disease*" OR "concomitant medical illness*" OR "concomitant clinical disease*" OR "concomitant medical disease*" OR "concurrent condition*" OR "concurrent issue*" OR "concurrent clinical condition*" OR "concurrent medical condition*" OR "concurrent medical issue*" OR "concurrent health condition*" OR "concurrent health issue*" OR "concurrent illness*" OR "concurrent disease*" OR "concurrent medical illness*" OR "concurrent medical disease*" OR "co-existing condition*" OR "coexisting condition*"OR "coexisting medical condition*"OR "co-existing medical condition*" OR "coexisting health condition*"OR "co-existing health condition*" OR "coexisting illness*"OR "co-existing illness*" OR "coexisting disease*"OR "co-existing disease*" OR "coexisting medical illness*"OR "co-existing medical illness*" OR "chronic healthcare condition*" OR “coexisting issue*” OR “chronic clinical issue*” OR “chronic health care condition*” OR “chronic health care issue*” OR “chronic clinical illness*” OR “multiple health care condition*” OR “multiple health care issue*” OR “multiple healthcare condition*” OR “multiple healthcare issue*” OR “multiple clinical illness*” OR “concomitant clinical issue*” OR “concomitant health care condition*” OR “concomitant health care issue*” OR “concomitant healthcare condition*” OR “concomitant healthcare issue*” OR “concomitant clinical illness*” OR “concurrent clinical issue*” OR “concurrent health care condition*” OR “concurrent health care issue*” OR “concurrent healthcare condition*” OR “concurrent healthcare issue*” OR “concurrent clinical illness*” OR “concurrent clinical disease*” OR “co-existing issue*” OR “co-existing clinical condition*” OR “co-existing clinical issue*” OR “co-existing medical issue*” OR “co-existing health issue*” OR “co-existing health care condition*” OR “co-existing health care issue*” OR “co-existing healthcare condition*” OR “co-existing healthcare issue*” OR “co-existing clinical illness*” OR “co-existing clinical disease*” OR “co-existing medical disease*” OR “coexisting issue*” OR “coexisting clinical condition*” OR “coexisting clinical issue*” OR “coexisting medical issue*” OR “coexisting health issue*” OR “coexisting health care condition*” OR “coexisting health care issue*” OR “coexisting healthcare condition*” OR “coexisting healthcare issue*” OR “coexisting clinical illness*” OR “coexisting clinical disease*” OR “coexisting medical disease*”) OR AB("multimorbidity" OR "multi-morbidity" OR "multimorbidities" OR "multi-morbidities" OR "comorbidity" OR "co-morbidity" OR "comorbidities" OR "co-morbidities" OR "chronic condition*" OR "chronic issue*" OR "chronic clinical condition*" OR "chronic medical condition*" OR "chronic medical issue*" OR "chronic health condition*" OR "chronic health issue*" OR "chronic illness*" OR "chronic medical illness*" OR "chronic disease*" OR "chronic medical disease*" OR "chronic clinical disease*" OR "multiple condition*" OR "multiple issue*" OR "multiple clinical condition*" OR "multiple clinical issue*" OR "multiple medical condition*" OR "multiple medical issue*" OR "multiple health condition*" OR "multiple health issue*" OR "multiple illness*" OR

	"multiple disease*" OR "multiple medical illness*" OR "multiple clinical disease*" OR "multiple medical disease*" OR "concomitant condition*" OR "concomitant issue*" OR "concomitant clinical condition*" OR "concomitant medical condition*" OR "concomitant medical issue*" OR "concomitant health condition*" OR "concomitant health issue*" OR "concomitant illness*" OR "concomitant disease*" OR "concomitant medical illness*" OR "concomitant clinical disease*" OR "concomitant medical disease*" OR "concurrent condition*" OR "concurrent issue*" OR "concurrent clinical condition*" OR "concurrent medical condition*" OR "concurrent medical issue*" OR "concurrent health condition*" OR "concurrent health issue*" OR "concurrent illness*" OR "concurrent disease*" OR "concurrent medical illness*" OR "concurrent medical disease*" OR "co-existing condition*" OR "coexisting condition*"OR "coexisting medical condition*"OR "co-existing medical condition*" OR "coexisting health condition*"OR "co-existing health condition*" OR "coexisting illness*"OR "co-existing illness*" OR "coexisting disease*"OR "co-existing disease*" OR "coexisting medical illness*"OR "co-existing medical illness*" OR "chronic healthcare condition*" OR “coexisting issue*” OR “chronic clinical issue*” OR “chronic health care condition*” OR “chronic health care issue*” OR “chronic clinical illness*” OR “multiple health care condition*” OR “multiple health care issue*” OR “multiple healthcare condition*” OR “multiple healthcare issue*” OR “multiple clinical illness*” OR “concomitant clinical issue*” OR “concomitant health care condition*” OR “concomitant health care issue*” OR “concomitant healthcare condition*” OR “concomitant healthcare issue*” OR “concomitant clinical illness*” OR “concurrent clinical issue*” OR “concurrent health care condition*” OR “concurrent health care issue*” OR “concurrent healthcare condition*” OR “concurrent healthcare issue*” OR “concurrent clinical illness*” OR “concurrent clinical disease*” OR “co-existing issue*” OR “co-existing clinical condition*” OR “co-existing clinical issue*” OR “co-existing medical issue*” OR “co-existing health issue*” OR “co-existing health care condition*” OR “co-existing health care issue*” OR “co-existing healthcare condition*” OR “co-existing healthcare issue*” OR “co-existing clinical illness*” OR “co-existing clinical disease*” OR “co-existing medical disease*” OR “coexisting issue*” OR “coexisting clinical condition*” OR “coexisting clinical issue*” OR “coexisting medical issue*” OR “coexisting health issue*” OR “coexisting health care condition*” OR “coexisting health care issue*” OR “coexisting healthcare condition*” OR “coexisting healthcare issue*” OR “coexisting clinical illness*” OR “coexisting clinical disease*” OR “coexisting medical disease*”)
Generalist-led Care	MH (“Physicians, family+” OR “Family practice+” OR “Geriatrics+” OR “Multidisciplinary care team+” OR “Health services for the aged+” OR “Geriatric assessment+”) OR TI("Family medicine" OR "internal medicine" OR "internist*" OR "geriatric*" OR "gerontology" OR "generalist*" OR "general medicine" OR "general practice" OR "family physician*" OR "care team*" OR "healthcare team*"OR "health team*" OR "interdisciplinary" OR "multidisciplinary" OR "general practitioner*" OR "general care" OR "generalized care" OR “generalised care” OR "integrated care" OR "interprofessional" OR "team-based") OR AB("Family medicine" OR "internal medicine" OR "internist*" OR "geriatric*" OR "gerontology" OR "generalist*" OR "general medicine" OR "general practice" OR "family physician*" OR "care team*" OR "healthcare team*"OR "health team*" OR "interdisciplinary" OR "multidisciplinary" OR "general practitioner*" OR "general care" OR "generalized care" OR “generalised care” OR "integrated care" OR "interprofessional" OR "team-based")
Performance Management	MH (“Accountability+” OR “Practice guidelines+” OR “Professional compliance+” OR “Public reporting of healthcare data+” OR “Guideline adherence+” OR “Clinical governance+” OR “Outcomes (health care)+” OR “Clinical competence+” OR “Quality assurance+” OR “Practice patterns+” OR “Goals and objectives+” OR “Clinical indicators+” OR “Personnel management+” OR “Performance measurement systems+” OR “Employee performance appraisal+” OR “Job performance+”) OR TI("perform*" OR "outcome*" OR "output*" OR "out put*"OR "management" OR "review*" OR "evaluation*" OR "rating*" OR "rank*" OR "dialogue*" OR "feedback*" OR "result*" OR "measure*" OR "metric*" OR "goal*" OR "objective*" OR "target*" OR "benchmark*" OR "benchlearn*" OR "criteria" OR "criterion" OR "standard*" OR "indicator*" OR "score*" OR "scoring" OR "competence*" OR

	"grading" OR "grade*" OR "assess*" OR "rate*" OR "monitor*" OR "audit*" OR "report*" OR "recommend*" OR "guide*" OR "guiding" OR "protocol*" OR "measurable*" OR "quality" OR "success*" OR "practice*" OR "effective*" OR "compliance" OR "comply*"OR "adher*" OR "six sigma" OR "Donabedian" OR "critiqu*" OR "appraisal*" OR "accountab*") OR AB("perform*" OR "outcome*" OR "output*" OR "out put*"OR "management" OR "review*" OR "evaluation*" OR "rating*" OR "rank*" OR "dialogue*" OR "feedback*" OR "result*" OR "measure*" OR "metric*" OR "goal*" OR "objective*" OR "target*" OR "benchmark*" OR "benchlearn*" OR "criteria" OR "criterion" OR "standard*" OR "indicator*" OR "score*" OR "scoring" OR "competence*" OR "grading" OR "grade*" OR "assess*" OR "rate*" OR "monitor*" OR "audit*" OR "report*" OR "recommend*" OR "guide*" OR "guiding" OR "protocol*" OR "measurable*" OR "quality" OR "success*" OR "practice*" OR "effective*" OR "compliance" OR "comply*"OR "adher*" OR "six sigma" OR "Donabedian" OR "critiqu*" OR "appraisal*" OR "accountab*")
Value-Based Care	MH (“Motivation+” OR “Reimbursement, incentive+” OR “Employee incentive programs+” OR “Physician incentive plans+” OR “Salaries and fringe benefits+” OR “Health care reform+” OR “Value-based purchasing+” OR “Value-based insurance+” OR “Value-based health care+”) OR TI("health outcome*" OR "health care outcome*" OR "healthcare outcome*" OR "healthcare delivery outcome*" OR "care delivery outcome*" OR "care outcome*" OR "intervention outcome*" OR "outcome improvement*" OR "better outcome*" OR "improved outcome*" OR "improving outcome*" OR "health improvement*" OR "improved health" OR "improving health" OR "better health" OR "value*" OR "result focused" OR "results focused" OR "result driven" OR "results driven" OR "result based" OR "results based" OR "merit increase*" OR "merit raise*" OR "performance based" OR "performance centric" OR "performance centered" OR "performance driven" OR "performance focused" OR "performance centred" OR "outcome based" OR "outcome centered" OR "outcome centred" OR "outcomes centered" OR "outcomes based" OR "outcome centric" OR "outcome driven" OR "outcomes driven" OR "outcomes focussed" OR "outcomes focused" OR "outcome focussed" OR "outcome focused" OR "bundled payment*" OR "pay for performance" OR "payment for performance" OR "pay" OR "p4p" OR "payment*" OR "reward*" OR "bonus*" OR "incentive*" OR "contract" OR "contracts" OR "non-payment" OR "nonpayment" OR "financ*" OR "monetary" OR "paid" OR "paying" OR "penalt*" OR "money" OR "cash" OR "fund*" OR "fee" OR "fees" OR "econom*" OR "disburs*" OR "renumerat*" OR "reimburse*" OR "compensat*" OR "incentive*" OR "function centric" OR "function based" OR "function driven" OR "function centered" OR "function centred" OR "quality of care" OR "quality of health care" OR "quality of healthcare" OR "quality based" OR "cost effective*" OR "costs reduction*" OR "cost reduction*" OR "reduced cost*" OR "lowered cost*" OR "low cost*" OR "lowest cost*" OR "reasonable cost*" OR "minimized cost*" OR "cost minimization*" OR "cost minimisation*"OR "costs minimization*" OR "cost improvement*" OR "improved cost*" OR “result centric” OR “results centric” OR “result focussed” OR “results focussed” OR “result centered” OR “result centred” OR “results centred” OR “results centered” OR “performance focussed” OR “outcomes centred” OR “outcomes centric” OR “minimised cost*” OR “costs minimisation*”) OR AB("health outcome*" OR "health care outcome*" OR "healthcare outcome*" OR "healthcare delivery outcome*" OR "care delivery outcome*" OR "care outcome*" OR "intervention outcome*" OR "outcome improvement*" OR "better outcome*" OR "improved outcome*" OR "improving outcome*" OR "health improvement*" OR "improved health" OR "improving health" OR "better health" OR "value*" OR "result focused" OR "results focused" OR "result driven" OR "results driven" OR "result based" OR "results based" OR "merit increase*" OR "merit raise*" OR "performance based" OR "performance centric" OR "performance centered" OR "performance driven" OR "performance focused" OR "performance centred" OR "outcome based" OR "outcome centered" OR "outcome centred" OR "outcomes centered" OR "outcomes based" OR "outcome centric" OR "outcome driven" OR "outcomes driven" OR "outcomes focussed" OR "outcomes focused" OR "outcome focussed" OR "outcome focused" OR "bundled payment*" OR "pay for performance" OR "payment for performance" OR "pay" OR "p4p" OR

	"payment*" OR "reward*" OR "bonus*" OR "incentive*" OR "contract" OR "contracts" OR "non-payment" OR "nonpayment" OR "financ*" OR "monetary" OR "paid" OR "paying" OR "penalt*" OR "money" OR "cash" OR "fund*" OR "fee" OR "fees" OR "econom*" OR "disburs*" OR "renumerat*" OR "reimburse*" OR "compensat*" OR "incentive*" OR "function centric" OR "function based" OR "function driven" OR "function centered" OR "function centred" OR "quality of care" OR "quality of health care" OR "quality of healthcare" OR "quality based" OR "cost effective*" OR "costs reduction*" OR "cost reduction*" OR "reduced cost*" OR "lowered cost*" OR "low cost*" OR "lowest cost*" OR "reasonable cost*" OR "minimized cost*" OR "cost minimization*" OR "cost minimisation*"OR "costs minimization*" OR "cost improvement*" OR "improved cost*" OR “result centric” OR “results centric” OR “result focussed” OR “results focussed” OR “result centered” OR “result centred” OR “results centred” OR “results centered” OR “performance focussed” OR “outcomes centred” OR “outcomes centric” OR “minimised cost*” OR “costs minimisation*”)

Web of Science Search Strategy

The searches for the four domains were joined by “AND” to produce the final set of search results.

Refined by: Publication Years: 2006 to 2021

Indexes=SCI-EXPANDED, SSCI, A&HCI, CPCI-S, ESCI Timespan=All years

Concept Domain	Search Terms
Multimorbidity	TS=("multimorbidity" OR "multi-morbidity" OR "multimorbidities" OR "multi-morbidities" OR "comorbidity" OR "co-morbidity" OR "comorbidities" OR "co-morbidities" OR "chronic condition*" OR "chronic issue*" OR "chronic clinical condition*" OR "chronic medical condition*" OR "chronic medical issue*" OR "chronic health condition*" OR "chronic health issue*" OR "chronic illness*" OR "chronic medical illness*" OR "chronic disease*" OR "chronic medical disease*" OR "chronic clinical disease*" OR "multiple condition*" OR "multiple issue*" OR "multiple clinical condition*" OR "multiple clinical issue*" OR "multiple medical condition*" OR "multiple medical issue*" OR "multiple health condition*" OR "multiple health issue*" OR "multiple illness*" OR "multiple disease*" OR "multiple medical illness*" OR "multiple clinical disease*" OR "multiple medical disease*" OR "concomitant condition*" OR "concomitant issue*" OR "concomitant clinical condition*" OR "concomitant medical condition*" OR "concomitant medical issue*" OR "concomitant health condition*" OR "concomitant health issue*" OR "concomitant illness*" OR "concomitant disease*" OR "concomitant medical illness*" OR "concomitant clinical disease*" OR "concomitant medical disease*" OR "concurrent condition*" OR "concurrent issue*" OR "concurrent clinical condition*" OR "concurrent medical condition*" OR "concurrent medical issue*" OR "concurrent health condition*" OR "concurrent health issue*" OR "concurrent illness*" OR "concurrent disease*" OR "concurrent medical illness*" OR "concurrent medical disease*" OR "co-existing condition*" OR "coexisting condition*"OR "coexisting medical condition*"OR "co-existing medical condition*" OR "coexisting health condition*"OR "co-existing health condition*" OR "coexisting illness*"OR "co-existing illness*" OR "coexisting disease*"OR "co-existing disease*" OR "coexisting medical illness*"OR "co-existing medical illness*" OR "chronic healthcare condition*" OR “coexisting issue*” OR “chronic clinical issue*” OR “chronic health care condition*” OR “chronic health care issue*” OR “chronic clinical illness*” OR “multiple health care condition*” OR “multiple health care issue*” OR “multiple healthcare condition*” OR “multiple healthcare issue*” OR “multiple clinical illness*” OR “concomitant clinical issue*” OR “concomitant health care condition*” OR “concomitant health care issue*” OR “concomitant healthcare condition*” OR “concomitant healthcare issue*” OR “concomitant clinical illness*” OR “concurrent clinical issue*” OR “concurrent health care condition*” OR “concurrent health care

	issue*” OR “concurrent healthcare condition*” OR “concurrent healthcare issue*” OR “concurrent clinical illness*” OR “concurrent clinical disease*” OR “co-existing issue*” OR “co-existing clinical condition*” OR “co-existing clinical issue*” OR “co-existing medical issue*” OR “co-existing health issue*” OR “co-existing health care condition*” OR “co-existing health care issue*” OR “co-existing healthcare condition*” OR “co-existing healthcare issue*” OR “co-existing clinical illness*” OR “co-existing clinical disease*” OR “co-existing medical disease*” OR “coexisting issue*” OR “coexisting clinical condition*” OR “coexisting clinical issue*” OR “coexisting medical issue*” OR “coexisting health issue*” OR “coexisting health care condition*” OR “coexisting health care issue*” OR “coexisting healthcare condition*” OR “coexisting healthcare issue*” OR “coexisting clinical illness*” OR “coexisting clinical disease*” OR “coexisting medical disease*”)
Generalist-led Care	TS=("Family medicine" OR "internal medicine" OR "internist*" OR "geriatric*" OR "gerontology" OR "generalist*" OR "general medicine" OR "general practice" OR "family physician*" OR "care team*" OR "healthcare team*"OR "health team*" OR "interdisciplinary" OR "multidisciplinary" OR "general practitioner*" OR "general care" OR "generalized care" OR “generalised care” OR "integrated care" OR "interprofessional" OR "team-based")
Performance Management	TS=("perform*" OR "outcome*" OR "output*" OR "out put*"OR "management" OR "review*" OR "evaluation*" OR "rating*" OR "rank*" OR "dialogue*" OR "feedback*" OR "result*" OR "measure*" OR "metric*" OR "goal*" OR "objective*" OR "target*" OR "benchmark*" OR "benchlearn*" OR "criteria" OR "criterion" OR "standard*" OR "indicator*" OR "score*" OR "scoring" OR "competence*" OR "grading" OR "grade*" OR "assess*" OR "rate*" OR "monitor*" OR "audit*" OR "report*" OR "recommend*" OR "guide*" OR "guiding" OR "protocol*" OR "measurable*" OR "quality" OR "success*" OR "practice*" OR "effective*" OR "compliance" OR "comply*"OR "adher*" OR "six sigma" OR "Donabedian" OR "critiqu*" OR "appraisal*" OR "accountab*")
Value-Based Care	TS=("health outcome*" OR "health care outcome*" OR "healthcare outcome*" OR "healthcare delivery outcome*" OR "care delivery outcome*" OR "care outcome*" OR "intervention outcome*" OR "outcome improvement*" OR "better outcome*" OR "improved outcome*" OR "improving outcome*" OR "health improvement*" OR "improved health" OR "improving health" OR "better health" OR "value*" OR "result focused" OR "results focused" OR "result driven" OR "results driven" OR "result based" OR "results based" OR "merit increase*" OR "merit raise*" OR "performance based" OR "performance centric" OR "performance centered" OR "performance driven" OR "performance focused" OR "performance centred" OR "outcome based" OR "outcome centered" OR "outcome centred" OR "outcomes centered" OR "outcomes based" OR "outcome centric" OR "outcome driven" OR "outcomes driven" OR "outcomes focussed" OR "outcomes focused" OR "outcome focussed" OR "outcome focused" OR "bundled payment*" OR "pay for performance" OR "payment for performance" OR "pay" OR "p4p" OR "payment*" OR "reward*" OR "bonus*" OR "incentive*" OR "contract" OR "contracts" OR "non-payment" OR "nonpayment" OR "financ*" OR "monetary" OR "paid" OR "paying" OR "penalt*" OR "money" OR "cash" OR "fund*" OR "fee" OR "fees" OR "econom*" OR "disburs*" OR "renumerat*" OR "reimburse*" OR "compensat*" OR "incentive*" OR "function centric" OR "function based" OR "function driven" OR "function centered" OR "function centred" OR "quality of care" OR "quality of health care" OR "quality of healthcare" OR "quality based" OR "cost effective*" OR "costs reduction*" OR "cost reduction*" OR "reduced cost*" OR "lowered cost*" OR "low cost*" OR "lowest cost*" OR "reasonable cost*" OR "minimized cost*" OR "cost minimization*" OR "cost minimisation*"OR "costs minimization*" OR "cost improvement*" OR "improved cost*" OR “result centric” OR “results centric” OR “result focussed” OR “results focussed” OR “result centered” OR “result centred” OR “results centred” OR “results centered” OR “performance focussed” OR “outcomes centred” OR “outcomes centric” OR “minimised cost*” OR “costs minimisation*”)

Scopus Search Strategy

The searches for the four domains were joined by “AND” to produce the final set of search results.

The publication years were limited from 2006 to 2021. To facilitate exporting, the searches for 2006-2009, 2010-2013, 2014-2017 and 2018-2021 were conducted separately.

Concept Domain	Search Strategy
Multimorbidity	(TITLE-ABS-KEY ("multimorbidity" OR "multi-morbidity" OR "multimorbidities" OR "multi-morbidities" OR "comorbidity" OR "co-morbidity" OR "comorbidities" OR "co-morbidities" OR "chronic condition*" OR "chronic issue*" OR "chronic clinical condition*" OR "chronic medical condition*" OR "chronic medical issue*" OR "chronic health condition*" OR "chronic health issue*" OR "chronic illness*" OR "chronic medical illness*" OR "chronic disease*" OR "chronic medical disease*" OR "chronic clinical disease*" OR "multiple condition*" OR "multiple issue*" OR "multiple clinical condition*" OR "multiple clinical issue*" OR "multiple medical condition*" OR "multiple medical issue*" OR "multiple health condition*" OR "multiple health issue*" OR "multiple illness*" OR "multiple disease*" OR "multiple medical illness*" OR "multiple clinical disease*" OR "multiple medical disease*" OR "concomitant condition*" OR "concomitant issue*" OR "concomitant clinical condition*" OR "concomitant medical condition*" OR "concomitant medical issue*" OR "concomitant health condition*" OR "concomitant health issue*" OR "concomitant illness*" OR "concomitant disease*" OR "concomitant medical illness*" OR "concomitant clinical disease*" OR "concomitant medical disease*" OR "concurrent condition*" OR "concurrent issue*" OR "concurrent clinical condition*" OR "concurrent medical condition*" OR "concurrent medical issue*" OR "concurrent health condition*" OR "concurrent health issue*" OR "concurrent illness*" OR "concurrent disease*" OR "concurrent medical illness*" OR "concurrent medical disease*" OR "co-existing condition*" OR "coexisting condition*" OR "coexisting medical condition*" OR "co-existing medical condition*" OR "coexisting health condition*" OR "co-existing health condition*" OR "coexisting illness*" OR "co-existing illness*" OR "coexisting disease*" OR "co-existing disease*" OR "coexisting medical illness*" OR "co-existing medical illness*" OR "chronic healthcare condition*" OR "coexisting issue*" OR "chronic clinical issue*" OR "chronic health care condition*" OR "chronic health care issue*" OR "chronic clinical illness*" OR "multiple health care condition*" OR "multiple health care issue*" OR "multiple healthcare condition*" OR "multiple healthcare issue*" OR "multiple clinical illness*" OR "concomitant clinical issue*" OR "concomitant health care condition*" OR "concomitant health care issue*" OR "concomitant healthcare condition*" OR "concomitant healthcare issue*" OR "concomitant clinical illness*" OR "concurrent clinical issue*" OR "concurrent health care condition*" OR "concurrent health care issue*" OR "concurrent healthcare condition*" OR "concurrent healthcare issue*" OR "concurrent clinical illness*" OR "concurrent clinical disease*" OR "co-existing issue*" OR "co-existing clinical condition*" OR "co-existing clinical issue*" OR "co-existing medical issue*" OR "co-existing health issue*" OR "co-existing health care condition*" OR "co-existing health care issue*" OR "co-existing healthcare condition*" OR "co-existing healthcare issue*" OR "co-existing clinical illness*" OR "co-existing clinical disease*" OR "co-existing medical disease*" OR "coexisting issue*" OR "coexisting clinical condition*" OR "coexisting clinical issue*" OR "coexisting medical issue*" OR "coexisting health issue*" OR "coexisting health care condition*" OR "coexisting health care issue*" OR "coexisting healthcare condition*" OR "coexisting healthcare issue*" OR "coexisting clinical illness*" OR "coexisting clinical disease*" OR "coexisting medical disease*"))
Generalist-led Care	(TITLE-ABS-KEY ("Family medicine" OR "internal medicine" OR "internist*" OR "geriatric*" OR "gerontology" OR "generalist*" OR "general medicine" OR

	"general practice" OR "family physician*" OR "general practitioner*" OR "general care" OR "generalized care" OR "generalised care"))
Performance Management	(TITLE-ABS-KEY ("perform*" OR "outcome*" OR "output*" OR "out put*" OR "management" OR "review*" OR "evaluation*" OR "rating*" OR "rank*" OR "dialogue*" OR "feedback*" OR "result*" OR "measure*" OR "metric*" OR "goal*" OR "objective*" OR "target*" OR "benchmark*" OR "benchlearn*" OR "criteria" OR "criterion" OR "standard*" OR "indicator*" OR "score*" OR "scoring" OR "competence*" OR "grading" OR "grade*" OR "assess*" OR "rate*" OR "monitor*" OR "audit*" OR "report*" OR "recommend*" OR "guide*" OR "guiding" OR "protocol*" OR "measurable*" OR "quality" OR "success*" OR "practice*" OR "effective*" OR "compliance" OR "comply*" OR "adher*" OR "six sigma" OR "Donabedian" OR "critiqu*" OR "appraisal*" OR "accountab*"))
Value-Based Care	(TITLE-ABS-KEY ("health outcome*" OR "health care outcome*" OR "healthcare outcome*" OR "healthcare delivery outcome*" OR "care delivery outcome*" OR "care outcome*" OR "intervention outcome*" OR "outcome improvement*" OR "better outcome*" OR "improved outcome*" OR "improving outcome*" OR "value*" OR "result focused" OR "results focused" OR "result driven" OR "results driven" OR "result based" OR "results based" OR "merit increase*" OR "merit raise*" OR "performance based" OR "performance centric" OR "performance centered" OR "performance driven" OR "performance focused" OR "performance centred" OR "outcome based" OR "outcome centered" OR "outcome centred" OR "outcomes centered" OR "outcomes based" OR "outcome centric" OR "outcome driven" OR "outcomes driven" OR "outcomes focussed" OR "outcomes focused" OR "outcome focussed" OR "outcome focused" OR "bundled payment*" OR "pay for performance" OR "payment for performance" OR "p4p" OR "quality of care" OR "quality of health care" OR "quality of healthcare" OR "quality based" OR "cost effective*" OR "costs reduction*" OR "cost reduction*" OR "reduced cost*" OR "lowered cost*" OR "low cost*" OR "lowest cost*" OR "reasonable cost*" OR "minimized cost*" OR "cost minimization*" OR "cost minimisation*" OR "costs minimization*" OR "cost improvement*" OR "improved cost*" OR "result centric" OR "results centric" OR "result focussed" OR "results focussed" OR "result centered" OR "result centred" OR "results centred" OR "results centered" OR "performance focussed" OR "outcomes centred" OR "outcomes centric" OR "minimised cost*" OR "costs minimisation*"))

Appendix 3 Characteristics of Sources of Evidence (Data Charted)

Index	Title	Purpose	Study population
1	Association Between Treatment by Locum Tenens Internal Medicine Physicians and 30-Day Mortality Among Hospitalized Medicare Beneficiaries [17]	Evaluate quality and costs of care among hospitalized Medicare beneficiaries treated by locum tenens versus non–locum tenens physicians	Acute care hospitalizations identified in a random sample of Medicare fee-for-service beneficiaries 65 years and older, where the attending physician was a general internal medicine physician
2	Challenges and opportunities in pragmatic implementation of a holistic hospital care model in Singapore: A mixed-method case study [3]	Quantitatively summarise the clinical outcomes of a specialist-led General Medicine model implemented at an acute hospital in Singapore and qualitatively describe the challenges and learnings	The team of specialist physicians practising General Medicine consisted of physicians trained in family medicine, internal medicine, geriatric medicine, rehabilitation medicine and other specialties.
3	Comparison of Resident, Advanced Practice Clinician, and Hospitalist Teams in an Academic Medical Centre: Association with Clinical Outcomes and Resource Utilization [18]	Directly compare outcomes among resident, advanced practice clinician (APC), and solo hospitalist inpatient general medicine teams	The inpatient teams had the following structures: A "resident team" was composed of a senior resident and one to two medical students or one senior resident, two interns, and one to two medical students supervised by a hospitalist physician; An "APC team" was composed of one to two APCs supervised by a hospitalist physician; and a "hospitalist team" was composed of one attending hospitalist independently managing all patients. The patients were from the inpatient general internal medicine service.
4	Development of Resident-Sensitive Quality Measures for Inpatient General Internal Medicine [19]	Develop Resident-Sensitive Quality Measures for a general internal medicine inpatient residency rotation using previously established consensus methods.	Internal Medicine and Medicine-Paediatrics residents and faculty members were involved in the consensus methods used to obtain the measures. The measures were developed to apply to the general internal medicine inpatient ward rotation at a single institution
5	Investigating the effectiveness of care delivery at an acute geriatric community hospital for older adults in the Netherlands: a protocol for a prospective controlled observational study [20]	Compare care for older patients in the geriatrician-led acute geriatric community hospital versus in a hospital setting. This includes evaluating patient outcomes and cost effectiveness and identifying facilitators and barriers to the implementation.	Admitted Acute Geriatric Community Care Hospital (AGCH) patients have a combination of an acute medical problem requiring hospitalisation and a geriatric condition. They should not need care or tests that can only be provided in hospital or require transfer to a skilled nursing facility. The expected stay is a maximum of 14 days. For the cohort studies used as controls, the patients were admitted to internal medicine, cardiology, pulmonology and geriatrics departments. They should have diagnoses that are ambulatory care sensitive conditions. The control studies had participants hospitalised for at least 48 hours. The first had patients 65 years and older and the second had patients 70 years and older.
6	A novel organizational model to face the challenge of multimorbid elderly patients in an internal medicine setting: a case study from Parma Hospital, Italy [21]	Present an organizational model at the Internal Medicine and Critical Subacute Care Unit of Parma University Hospital which is a 106-bed internal medicine area organized by intensity of care and dedicated to multimorbid elderly patients	Internal Medicine and Critical Subacute Care Unit is an internal medicine area with hospitalist and resident physicians. It is split into High-intensity very-fast-turnover internal medicine ward ("come'n'go ward,"), High-intensity fast-turnover internal medicine ward (IM), Intermediate-intensity critical subacute care ward (CSC) and Integrated nurse-managed subacute care ward (INMSC). The IM ward and "come'n'go ward" admits from the Emergency Department (ED) patients with medical problems who are 65 or older, with multimorbidity, polypharmacy and disability status, frequent hospital admissions, have medical devices or seek medical care for complex social reasons. CSC ward patients are over 65 years old, with multimorbidity, polypharmacy, poor functional status, frailty, who have already spent a period of stay in acute-care units of the hospital. Patients in the INMSC ward do not need high medical intensity of care, only a skilled and intense nursing care for therapies and rehabilitation programmes.

**7**	Selecting best-suited "patient-related outcomes" in older people admitted to an acute geriatric or emergency frailty unit and applying quality improvement research to improve patient care [22]	A review to explore multimorbidity, frailty, the service model, and a range of “direct” and “indirect” patient-related outcomes of “at risk” older people admitted to an acute geriatric care unit. Data measurements and discussion of a quality improvement methodology to improve patient care based on patient-related outcome data	NIL (Review article)
**8**	Variation in physician spending and association with patient outcomes [23]	Investigate variation in spending across physicians and the association with patient outcomes	Medicare fee-for-service beneficiaries 65 years and older hospitalized with a nonelective medical condition and treated by a general internist or hospitalist at acute care hospitals
**9**	Census of Ligurian Internal Medicine Wards of non-teaching hospitals [24]	Survey the activities of hospital internal medicine to: i) define and compare the activities of each ward; ii) assess the impact of these activities on business management; iii) define the role of departments of internal medicine in the hospital organization; iv) formalize the role of emergency medicine and internal medicine v) provide tools of clinical governance in the definition of standards. Data was collected about medical staffing, equipment, skills, competencies and pro-ductivity	15 out of 18 Chiefs of 18 Ligurian non-university Internal Medicine wards
**10**	Pocket Change: A Simple Educational Intervention Increases Hospitalist Documentation of Comorbidities and Improves Hospital Quality Performance Measures [25]	We designed a pocket card reminder and educational intervention for hospitalists to improve documentation of 6 common comorbidities present on admission. We hypothesized that having this intervention in addition to just review by documentation specialists would be superior in capturing comorbidities present on admission and improving expected length of stay (LOS) and expected mortality.	10 internal medicine hospitalists at the University of Washington Medical Centre served as the attending physicians for 1 inpatient internal medicine house staff team and 1 hospitalist-only team. The control group was nonhospitalist attending physicians who led 2 inpatient internal medicine house staff teams. The patient subjects were admitted to these teams.
**11**	Using assessing care of vulnerable elders quality indicators to measure quality of hospital care for vulnerable elders [26]	Assess quality of care for hospitalized vulnerable elders using measures based on Assessing Care of Vulnerable Elders (ACOVE) quality indicators.	Subjects aged 65 and older hospitalized on the general medicine inpatient service who were defined as vulnerable using the Vulnerable Elder Survey-13 (VES-13)

**12**	Two European Examples of Acute Geriatric Units Located Outside of a General Hospital for Older Adults With Exacerbated Chronic Conditions [27]	This study aims to compare patients' diagnoses, characteristics, and outcomes of 2 European sites of acute geriatric units in intermediate care outside a general hospital, where acute medical care and early rehabilitation are provided to selected older patients.	Acute geriatric units in intermediate care provide acute medical care and early rehabilitation for patients with exacerbations of chronic conditions or “minor” acute events who may have complex social or functional problems. The Subacute Care Unit in Spain requires presence of geriatric conditions. Care is delivered at a unit within an intermediate care facility and led by a geriatrician. It provides acute and subacute care to older adults. In the Acute Geriatric Community Hospital in Netherlands care may also be led by an internist. It is a skilled nursing facility and has a geriatric rehabilitation unit and acute geriatric care unit.
**13**	Short-term Resource Utilization and Cost-Effectiveness of Comprehensive Geriatric Assessment in Acute Hospital Care for Severely Frail Elderly Patients [28]	Estimate the 3-month within-trial cost-effectiveness of comprehensive geriatric assessment (CGA) in acute medical care for frail elderly patients compared to usual care, by estimating health-related quality of life and costs from a societal perspective.	Patients aged 75 years and older in need of acute in-hospital treatment, frail according to validated FRail Elderly Support researcH group (FRESH) screening instrument and do not meet exclusion criteria (e.g., clearly suited for conventional acute care due to the medical condition.) Patients were allocated to the CGA acute elderly care unit for the intervention group, and conventional acute medical care unit for the control group.
**14**	RECALMIN: The association between management of Spanish National Health Service Internal Medical Units and health outcomes [29]	Investigate the association between management of Internal Medical Units (IMUs) with outcomes (mortality and length of stay)	IMUs in acute general hospitals are headed by an internal medicine specialist. IMUs in hospitals with <500 beds generally include other specialties while in acute general hospitals with >500 beds medical specialties have their own medical department
**15**	Quality of care factors associated with unplanned readmissions of older medical patients: a case-control study [30]	Assess the type and prevalence of quality of care factors associated with potentially preventable readmissions at a tertiary hospital general medicine service. Identify patient characteristics associated with quality factors.	Patients 65 years or older admitted acutely for more than 24 hours to the general medicine service of a tertiary hospital. Readmitted cases were patients discharged from and readmitted to the general medicine service under study within 30 days of discharge from index admission. They were selected on the basis of the most prevalent primary discharge diagnoses assigned to index admissions to the service during the study period. Index admissions were randomly selected with a frequency distribution similar to that of primary discharge diagnoses, and all corresponding 30-day readmissions were identified.
**16**	Quality of care delivered by general internists in US hospitals who graduated from foreign versus US medical schools: observational study [31]	Determine if patient outcomes differ between general internists who graduated from a medical school in the United States and outside the United States	Medicare fee-for-service beneficiaries aged 65 years or older admitted to hospital with a medical condition and treated by international or US medical graduates (from allopathic medical schools) who were general internists (including both hospitalists and non-hospitalists)
**17**	Evaluating Outcomes from an Integrated Health Service for Older Patients [32]	Compare the demographics and outcomes of patients from the Older Person Evaluation Review and Assessment (OPERA) service to those from the General Medicine (GM) service to contribute to a baseline for future analysis and to quantify the benefits of a dedicated CGA model for the management of older patients with acute illness. The OPERA service ran parallel to the general medicine service, offering alternative treatment with more comprehensive care led by a geriatrician. It focused on integrating the multidisciplinary needs of older patients requiring acute	Patients >75 years of age who had an acute admission to the OPERA or GM service were included. Data were collected for the entire admission period including any subacute care. Patients who had received acute care from another dedicated care pathway for >48 hours were excluded. Patients in the OPERA service should have a medical condition not requiring another dedicated care pathway, complex care needs and cognitive decline or functional decline. The OPERA service had a dedicated multidisciplinary team and a geriatrician.

		hospitalisation and providing support and intervention after discharge.	
**18**	Relationship Between Quality of Care and Functional Decline in Hospitalized Vulnerable Elders [33]	To assess the relationship between process of care based quality indicators for hospitalized elders and functional decline	General medicine inpatients at the University of Chicago Medical Centre who are over age 65 with a Vulnerable Elders Survey VES-13 score of 3 or above. Patients with a less than one day length of stay, patients who died in the hospital, were discharged to hospice, or were initially admitted to an intensive care unit (ICU) were also excluded.
**19**	Patient experiences and predictors in an acute geriatric ward: A cross-sectional study [34]	To investigate the elderly's experiences with acute hospital treatment and care, and the influence of socio-demographic variables, length of stay, comorbidity and self-rated health on these.	Patients in an acute geriatric, internal medicine ward, admitted due to symptoms from the respiratory and circulatory system, as well as infections and malnutrition/dehydration, who had stayed at the ward for a minimum 24 hours, without cognitive impairment, and were discharged alive.
**20**	Patient characteristics, resource use and outcomes associated with general internal medicine hospital care: the General Medicine Inpatient Initiative (GEMINI) retrospective cohort study [35]	Describe the demographic characteristics, medical conditions, health outcomes and resource use of patients admitted to general internal medicine at 7 hospital sites	The GEMINI cohort included patients who were admitted to or discharged from the general internal medicine service which includes hospitalist services and clinical teaching units which involve undergraduate and postgraduate medical trainees. The attending physicians in general internal medicine included mainly general internists or internal medicine subspecialists and a minority that were family physicians.
**21**	Implementation of a Physician Assistant/Hospitalist Service in an Academic Medical Centre: Impact on Efficiency and Patient Outcomes [36]	Evaluate and compare the quality and efficiency of patient care on a physician assistant/hospitalist service with traditional house staff services.	General medicine patients admitted to the Physician Assistant/Clinician Educator (PACE) service. It is staffed with physician assistants with supervision by hospitalists who were recent graduates of the study hospitals internal medicine residency with no prior clinical experience beyond residency. It is medically staffed by 1 hospitalist and 2 physician assistants from 7:00 AM to 7:00 PM on weekdays and by 1 hospitalist, 1 physician assistant, and 1 moonlighter from 7:00 AM to 7:00 PM on weekends. A moonlighter is usually a senior resident or medical subspecialty fellow that admitted patients and covered nights from 7:00 PM to 7:00 AM 7 days a week. The comparison group consisted of general medicine patients admitted to the 8 house staff general medicine teams; patients transferred from an intensive care unit (ICU) or another facility were excluded.
**22**	Impact of hospitalists on care outcomes in a large integrated health system in British Columbia [37]	Study care outcomes associated with a network of hospitalist services compared to "traditional" providers (community-based Family Physicians and Internal Medicine	Other than hospitalists and Family Physicians, there are a number of general internists and other subspecialists who accept Most Responsible Provider roles for general medicine patients who may present with higher-acuity conditions. The study included patients requiring hospitalisation in acute care hospitals due to nonsurgical or noncritical care conditions who are cared for by physicians belonging to the hospitalist, family

		specialists) in a large integrated health care system.	physician or internal medicine physician provider groups, where patients were admitted to and discharged by the same service. Facilities without hospitalist services were excluded.
**23**	Similar Outcomes Among General Medicine Patients Discharged on Weekends [38]	Determine whether post discharge outcomes differed for patients discharged on weekends where there are reduced staffing levels and services versus weekdays.	Unplanned adult admissions that consisted of a single, acute episode-of-care within the one hospital, where the discharging clinical unit was a general medical unit, and the patient was discharged home or to an aged-care facility.
**24**	Family Medicine Patients Have Shorter Length of Stay When Cared for on a Family Medicine Inpatient Service [39]	Test the hypothesis that local family medicine patients admitted to a family medicine inpatient service have shorter length of stay than those admitted to general hospitalist services which also care for tertiary patients at an academic medical centre.	The Family Medicine Inpatient (FMI) service admits any Department of Family Medicine (DFM) primary care patient who requires general inpatient medical care. The FMI service is a teaching service at an academic medical centre, staffed by a family medicine board–certified attending physician, a senior family medicine resident, a junior family medicine resident and 1 to 2 family medicine interns. Patients outside the DFM are cared for by Hospital Internal Medicine (HIM) when they require general inpatient medical care, although occasionally patients under a DFM care provider are erroneously admitted to a HIM service. Four HIM services are resident teaching services staffed by a hospitalist or general internist and residents, 1 is a fellowship service staffed by hospital medicine fellows, 2 are medical services for patients with active hematologic or solid organ malignancies, and the remainder are traditional hospitalist services staffed by a hospitalist and a nurse practitioner or physician assistant.
**25**	Does Doctor–Patient Communication Affect Patient Satisfaction with Hospital Care? Results of an Analysis with a Novel Instrumental Variable [40]	Determine the relationship between physicians' communication behaviours and patients' overall satisfaction with hospital care using a novel instrumental variable to address possible confounding of this association by patient attributes	Patients admitted to the University of Chicago Hospital's General Internal Medicine service. Physicians were faculty of the Department of Medicine of the University of Chicago.
**26**	Relationship Between Quality of Care for Hospitalized Vulnerable Elders and Post-Discharge Mortality [41]	Assess the relationship between quality of hospital care, as measured by ACOVE quality indicators, and post-discharge mortality for hospitalised elderly patients	Patients admitted to the general medicine inpatient service at the University of Chicago Medical Centre. Patients included in the study were age 65 and older and have a score on the Vulnerable Elders Survey (VES-13) of 3 or higher, identifying them as vulnerable. Such patients who survived to discharge were included in the study. All patients who were discharged to hospice or comfort care, or were hospitalised for one day or less, or were transferred from ICU were excluded.
**27**	Improving Patient Care Chronic Complex: Sub-Acute Care Unit [42]	Description of the general characteristics of the sub-acute care of Vallparadís Healthcare and their assistance activity	The unit of sub-acute care comprehensive medical facility of intermediate hospitalization
**28**	Analysis of Casuistry and Results of the Implementation of a Care Programme in Ten Sub-Acute Care Units in Catalonia [43]	Appraise the improvement in care for people with complex chronic illnesses, through the implementation of sub-acute care units for intermediate care. There was comprehensive and integrated on-going care and resources were adapted to patient needs. The care process, outcomes and their adjustment to the standards established were analysed.	Patients in sub-acute care units in intermediate care hospitals which aim to provide a geriatric rehabilitation approach from the outset to achieve a care continuum for multi-pathological elderly patients with decompensation.

**29**	Patient Outcomes in Teaching Versus Nonteaching General Internal Medicine Services: A Systematic Review and Meta- Analysis [44]	Conduct a systematic review and meta-analysis to examine if outcomes differ for general internal medicine (GIM) patients admitted to teaching versus nonteaching services.	Patients 18 years old or older admitted to a GIM teaching service and those admitted to a GIM nonteaching service in the same institution. A teaching service team has an attending physician (academic/university-based or community-based physician) and postgraduate and/or undergraduate trainees. A nonteaching service team has a treating physician (academic/university-based or community-based physician) working with or without physician assistants or nurse practitioners but without postgraduate or undergraduate trainees.
**30**	Effects of an Acute Care for Elders Unit on Costs and 30-Day Readmissions [45]	To examine variable direct costs from an interdisciplinary Acute Care for Elders (ACE) Unit compared with a multidisciplinary usual care (UC) unit which are both hospitalist medical units.	Patients requiring admission for acute medical illness that were assigned to the hospitalist service and admitted to the ACE (intervention) or UC unit (control), which depends on bed availability, which are 70 years or older, met inpatient admission criteria and spent the entirety of their hospitalisation as a hospitalists' patient in the ACE or UC unit. The ACE unit involved geriatric assessments, geriatrician-led interdisciplinary team rounds, early discharge planning and communication of team recommendations to appropriate formal/informal care provider.

Appendix 4 Results of Sources of Evidence: Measures (Data Charted)

Index	Title	Process Measures	Clinical Outcome Measures	Reported Outcome Measures	Cost Outcomes	Other Outcome Measures	Ward/Hospital level non-outcome management indicators	Adjustments for Outcome Measures
1	Association Between Treatment by Locum Tenens Internal Medicine Physicians and 30-Day Mortality Among Hospitalized Medicare Beneficiaries		30-day mortality, 30-day readmission rates, and mean length of stay.		Inpatient costs of care, defined as total Medicare Part B spending per hospitalization			Patient age, sex, race/ethnicity, Medicaid eligibility status, patient-level Elixhauser comorbidity indicators for 31 conditions, admitting Major Diagnostic Category, day of week of admission, month of year of admission, hospital fixed effects
2	Challenges and opportunities in pragmatic implementation of a holistic hospital care model in Singapore: A mixed-method case study		Average length of stay, 30-day readmission rate, inpatient mortality		Total hospitalisation cost			
3	Comparison of Resident, Advanced Practice Clinician, and Hospitalist Teams in an Academic Medical Centre: Association with Clinical Outcomes and Resource Utilization		Length of stay in days, 30-day unplanned readmissions, inpatient mortality		Total direct cost	Resource utilisation (measure provider related differences in efficiency): Discharge order time (hours after midnight), discharge time (hours after midnight), consults per admission		Age, gender, Charlson comorbidity index, transfer from outside hospital and nighttime admission

4	Development of Resident-Sensitive Quality Measures for Inpatient General Internal Medicine	Not condition specific inpatient general internal medicine measures<br>1. Complete admission medication reconciliation<br>2. Complete discharge medication reconciliation<br>3. Document code status on admission<br>4. Document discharge weight in discharge summary in patients with congestive heart failure<br>5. Document goals of care discussions that result in code status changes<br>6. Document reason for not ordering deep venous thrombosis prophylaxis on admission<br>7. Document source of positive blood culture specimens<br>8. Medications listed in the discharge instructions match the discharge medication reconciliation<br>9. Medications listed in the discharge instructions match the discharge summary<br>10. Medications listed in the discharge summary match the discharge medication reconciliation<br>11. Order alcohol withdrawal order set for acute alcohol withdrawal<br>12. Order code status on admission<br>13. Order daily weights in patients with congestive heart failure exacerbation<br>14. Order deep venous thrombosis prophylaxis on admission<br>15. Order home noninvasive positive pressure ventilation on admission<br>16. Order intravenous proton						

		pump inhibitor for acute gastrointestinal bleed<br>17. Order repeat haemoglobin/haematocrit in patients with active gastrointestinal bleeding<br>18. Order seizure precautions in patients with seizure history or condition placing them at risk of seizure<br>19. Order suicide precautions for patients with self-harm or intentional ingestion<br>20. Order surgical team consult for acute abdomen<br>21. Update sign out document daily						
5	Investigating the effectiveness of care delivery at an acute geriatric community hospital for older adults in the Netherlands: a protocol for a prospective controlled observational study		All-cause mortality (assume 1, 3, 6 months post discharge) (Table also indicated at discharge but subsequent elaboration stated it will be assessed during follow-up) occurrence (presence and duration) of delirium using the Confusion Assessment Method (CAM) four-item short version (table indicated at discharge (and admission) but subsequently elaborated that it is measured Day 1 to 3 and only continued if there are signs of	Health-related quality of life (HRQoL) using EuroQoL-5D (EQ-5D) (1,3,6 months after discharge), patient satisfaction (8 questions on a 5-point Likert scale) (at discharge and 1 month post discharge in case missed out previously), Activities of Daily	Healthcare perspective: Direct medical costs funded through the Dutch Healthcare System in the 6 months after the admission to the AGCH.<br>Societal perspective: Estimation of the costs of informal care.	Social network and informal care measured by presence and frequency of informal care (presence, frequency per week, type of care (housekeeping and/or personal care) and from which persons (partners, children, other family member or neighbour/volunteers) (1,3,6 months post discharge)<br>('Social network and informal care was not listed in primary/secondary Outcomes list only under Data Collection)<br><br>Healthcare Utilisation measures:		

			delirium until symptoms subside), Delirium Observation Screening scale (table indicated at discharge (and admission) but subsequently elaborated that it is measured Day 1 to 3 and only continued when there is clinical suspicion of delirium), risk of developing delirium measured using Dutch Safety Management Program VMS patient screening (at discharge), occurrence of falls in the AGCH and the consequences of falls (indication for prolonged stay, diagnostics or injury) (assume at discharge. Table indicated at discharge, 1, 3, 6 months post discharge, but subsequent elaboration only mentioned at discharge), fall history of the number of falls in the past 6 months	Living (ADL)-functionin g, as defined by 15 item modified Katz-ADL scale, including ADLs and Instrument al Activities of Daily Living (IADLs). (Assume 1,3,6 months post discharge. Table only indicated measureme nt at admission but subsequent elaboration stated it is measured during follow-up). Numerical Rating Scale (NRS) (0 to 10) on fear of falling (assume at discharge, 1, 3, 6 months.)		3-month, 1 month and 6-month readmissions to AGCH or hospital (number of readmissions, total days of readmission, reasons for readmission and whether the readmission was planned or unplanned) 3-month unplanned readmission rate to AGCH or hospital was the primary outcome. (assume readmission rates measured at 1, 3, and 6 months only. Table also indicated at discharge but subsequent elaboration stated hospital readmissions outcome will be assessed during follow-up), Permanent Institutionalisation (long-term admission to a skilled nursing facility) (Assumed 1,3,6 months post discharge. Permanent institutionalisation was not indicated in table. Included in subsequent elaboration but time not stated), Temporary admission to a nursing home (days of temporary		

			(at discharge, 1, 3, 6 months post discharge)<br><br>(*At discharge is defined as within 48 hours before discharge)	NRS (0 to 10) on pain (at discharge, 1, 3, 6 months post discharge) NRS (0 to 10) on fatigue (at discharge, 1, 3, 6 months post discharge) Apathy measured using 3 items of the Geriatric Depression Scale (GDS) (1) 'Do you prefer to stay at home, rather than going out and doing new things', (2) 'Have you dropped many of your activities and interests?' and (3) 'Do you feel full of energy'.		admission to a skilled nursing or rehabilitation facility)(1,3,6 months post discharge), Home Care (frequency of home care, including housekeeping, personal and nursing care and hours of informal care)(1,3,6 months post discharge), Number of consultations by physiotherapists and dieticians (Assumed 1,3,6 months post discharge. Not listed in table, unless considered under "medical specialist care", but in subsequent elaboration), Number of consultations by general practitioners (1,3,6 months post discharge), Number of outpatient hospital visits(1,3,6 months post discharge), Number of ED visits(Assumed 1,3,6 months post discharge. Not indicated in table, subsequently stated as assessed during follow-up).<br>Medical care during admission and process of discharge: The diagnostics performed in the AGCH, revisits to the		

				(at discharge, 1, 3, 6 months post discharge) (NRS for pain and fatigue, apathy were not listed in primary/secondary Outcomes list only under Data Collection)<br><br>(*At discharge is defined as within 48 hours before discharge)		hospital, admissions to the hospital, discharge destination and time needed to send medical handovers to the general practitioner, length of stay in AGCH (all these measured at discharge)<br><br>Informal Caregiver satisfaction (8 questions on a 5 point Likert scale) (at discharge and 1 month post discharge in case missed out previously)		
6	A novel organizational model to face the challenge of multimorbid elderly patients in an internal medicine setting: a case study from Parma Hospital, Italy		Inhospital mortality, 30-day unplanned readmission rate, average length of stay (Acute and total hospital stay including CSC and INMSC)				Performance, Efficiency and Effectiveness Indicators: Hospital Discharge Records (HDR) Average Weight: ratio between DRG points produced in a specific discipline of the considered hospital and the number of discharged patients, Case	

							Mix Index: Clinical complexity, calculated as the ratio between the weight of admissions for each category of patients and the mean weight of admissions for the same category by all regional providers during the same year, Number of admissions, Comparative Performance Index (CPI): Operational efficiency of hospital units obtained from average LOS standardized for case-mix and weighed for the efficiency of other hospitals in the same healthcare system, Charlson-Deyo Index	

7	Selecting best-suited "patient-related outcomes" in older people admitted to an acute geriatric or emergency frailty unit and applying quality improvement research to improve patient care	Safe discharge planning and follow-up before discharge from an acute geriatric unit (AGU) with adequate involvement of the patient and the carer, factors leading to a delayed transfer of care to the community, Screening of delirium, appropriate treatment, and communication of the diagnosis to community physicians, an appropriate dementia care plan and staff training. Access to social workers and intermediate care services for an AGU, strategies and plans for carer assessment and engagement<br>(Author classified these as organisational-related outcomes)	LOS, 30-day emergency readmission rate (ERR) (and reason for admission), number of days spent at usual place of residence in the 90 days after discharge, inpatient mortality, 30-day mortality, 90-day mortality, or 1-year mortality, discharge to own home, usual place of residence, or new care home, or institutionalization. Need for a type of care package, increased dependency, or mobility at the time of discharge using a validated scale. Organisational level: Adverse impacts of hospitalisation (hospital acquired infections, pressure sores, inpatient falls (standardized incidence of the inpatient falls rate should be measured as the number of inpatient	Hospital Anxiety and Depression Scale (HADS) and the Geriatric Depression Scale (GDS), short-form GDS with 15 questions. Generic PROMS: EuroQol EQ-5D and the 36-Item Short-Form Health Survey (SF-36), Short Form 6-D (SF-6D). Disease-specific PROMs		In various combinations: staffing ratios, nurse burnout levels, nurse job dissatisfaction or satisfaction level of the staff, work-related stress, needle-stick injuries, happiness level, nurses' skill mix, sickness rate, recruitment and retention of staff, or gaps in the nursing rota. Regular staff surveys about work experience to compare parameters over time: a yearly appraisal, satisfaction with quality of work, discrimination, harassment, or abuse at work, the level of pay, flexible working, and equal work opportunities. The Practice Environment Scale of the Nursing Work Index (PES-NWI) can be used to measure the nursing practice environment and its impact on the patient outcome. Measuring caregiver burden: 29-item self-reported Zarit Caregiver Burden Interview (ZBI) scale, 22-item, 12-item and 4-item versions, 24-item Caregiver Burden		

			falls/1000 occupied bed days), impact of inpatient falls including inpatient hip fracture, discharge to a new care home, and mortality)			Inventory (CBI) or 10-item Burden Scale for Family Caregivers. Complaints and litigation costs (organisational level). Rate of unplanned general practice (GP) consultations (organisational measure)		
8	Variation in physician spending and association with patient outcomes		30-day readmission rate 30-day mortality rate		Total adjusted part B spending per hospitalisation			Patient characteristics (demographics-race, age in 5 year increments, sex, 27 existing comorbid conditions, primary diagnosis using Major Diagnostic Categories MS-DRG), median household income estimated from residential zip codes, indicators for Medicaid coverage, indicator for year, hospital fixed effects, physician characteristics (age in 5 year increments/sex, type of medical training-allopathic vs osteopathic, medical school graduated from)
9	Census of Ligurian Internal Medicine Wards of non-teaching hospitals				total costs of the ward	Length of stay (Productivity measure)	mean DRG weight, beds turnover rate (productivity measures)	

10	Pocket Change: A Simple Educational Intervention Increases Hospitalist Documentation of Comorbidities and Improves Hospital Quality Performance Measures							
11	Using assessing care of vulnerable elders quality indicators to measure quality of hospital care for vulnerable elders	General hospital care<br>Discharge planning: If a vulnerable elder is admitted to the hospital, then the discharge planning should begin within 48 hours.<br>Deep vein thrombosis (DVT) prevention: If a vulnerable elder is at very high risk for venous thrombosis, then the patient should have venous thromboembolism prophylaxis.<br>Nutritional status: If a vulnerable elder is hospitalized, then his or her nutritional status should be documented during the hospitalization by evaluation of oral intake or serum biochemical testing.<br>Pain management: Screening for pain: All vulnerable elders should be screened for chronic pain during the initial evaluation period.<br>Geriatric-prevalent conditions<br>Delirium and dementia<br>Admission evaluation of cognition: If a vulnerable elder is admitted to the hospital for any acute or chronic illness or any						

		surgical procedure, then the evaluation should include, within 24 hours, cognitive status.<br>Delirium evaluation and treatment: If a hospitalized elder has a definite or suspected diagnosis of delirium, then an evaluation for potentially precipitating factors must be undertaken and identified causes treated.<br>Screening for depression: If a vulnerable elder has dementia, then he or she should be screened for depression during the initial evaluation.<br>Restraints: If a vulnerable elder with dementia is to be physically restrained in the hospital, then the target behavioural disturbance or safety issue justifying use of the restraints must be identified to the consenting person and documented in the chart.<br>Restraints: If a vulnerable elder is physically restrained and the target behavioural disturbance requiring restraint is identified, then the healthcare team should include methods other than physical restraints in the care plan.<br>Physical function<br>Functional screening: If a vulnerable elder is admitted to a hospital or is new to a physician practice, then assessment of functional status should be documented.<br>Exercise prescription for gait problems and weakness: If a vulnerable elder is found to have problems with gait, strength, or endurance, then an exercise program should be offered.						

		Pressure ulcers<br>Risk assessment: If a vulnerable elder is admitted to an intensive care unit or a medical or surgical unit of a hospital and cannot reposition himself or herself or has limited ability to do so, then risk assessment for pressure ulcers should be done on admission.<br>Evaluation: If a vulnerable elder presents with a pressure ulcer, then the pressure ulcer should be assessed for location, depth and stage, size, and the presence of necrotic tissue.<br>Debridement: If a vulnerable elder presents with a full-thickness sacral or trochanteric pressure ulcer covered with necrotic debris or eschar, then debridement using sharp, mechanical, enzymatic, or autolytic procedures should be done within 3 days of diagnosis.<br>Cleansing: If a vulnerable elder has a Stage 2 or greater pressure ulcer, then a topical antiseptic should not be used on the wound.<br>Topical dressings: If a vulnerable elder presents with a clean full-thickness or partial-thickness pressure ulcer, then a moist wound-healing environment should be provided with topical dressings.						
12	Two European Examples of Acute Geriatric Units Located Outside of a General Hospital for Older Adults With		Mean length of stay, rates of discharge to original living situation and rates of discharge to other intermediate care units, death					

	Exacerbated Chronic Conditions		during admission, admission to acute hospital during admission					
13	Short-term Resource Utilization and Cost-Effectiveness of Comprehensive Geriatric Assessment in Acute Hospital Care for Severely Frail Elderly Patients		Mortality (3 month follow up) (Data collected in a previous publication)	HRQoL (at index care episode and 3 month follow up using interviewer administered version of the Health Utilities Index HUI-3 instrument) Quality adjusted life years obtained from HRQoL (with patient's survival time at 3 months)	Costs at 3 month follow up Primary care: doctors' visits, doctors other contacts (phone, letters, indirect), nurse visits, nurse other contacts (phone, letters, indirect), other caregivers visits, other caregivers other contacts (phone, letters, indirect) Hospital care: index care episode, inpatient care, outpatient care Municipal care: home-help services, home health care, days in special housing	Resource utilization at 3 month follow up Primary care: doctors' visits, Doctors other contacts (phone, letters, indirect), nurse visits, nurses other contacts (phone, letters, indirect), other caregivers visits, other caregivers other contacts (phone, letters, indirect) Hospital care: index care episode (inpatient days), inpatient care (days from discharge to follow up), inpatient care (index +3 month), outpatient care visits (open care events) Municipal care: hours of home-help services, home health care hours, days in special housing.		Age, sex and Charlson comorbidity index
14	RECALMIN: The association between management of Spanish National Health Service Internal Medical Units		In-hospital mortality rate, average length of stay in the IMU				Management indicators of the IMUs: Proxies for complexity were the following - number of beds, number of inhabitants in the influence	Adjust length of stay by DRG weight (complexity), mortality rate adjusted by Charlson Comorbidity Index (CCI) (LOS also taken into account)

	and health outcomes						area, mean Charlson index, mean weight of IMU Diagnostic-Related Groups (DRG) at the IMU level. Others - Patient safety committee at the IMU or hospital level, multidisciplinary ward rounds at IMU level	
15	Quality of care factors associated with unplanned readmissions of older medical patients: a case-control study	Diagnostic errors or avoidable failures to diagnose either primary presenting condition or associated abnormalities<br><br>Failure to manage adequately active comorbidities that resulted in readmission<br><br>Failure to manage adequately the primary clinical problem during admission<br><br>Failure to assess adequately needs and limitations<br><br>Failure to educate adequately patient and carers about the clinical management of disease<br><br>Failure to communicate adequately details of care to community-based or nursing home care providers<br><br>Failure to formulate or activate advance care plan in eligible patients<br><br>Failure to formulate or activate	30-day unplanned readmissions (discharged from and readmitted to the general medicine service under study)<br><br>Potentially preventable complication of procedure or therapy undertaken/initiated during index admission, prompting readmission					

		palliative care plan for patients with clearly evident terminal illness<br><br>Failure to arrange follow up for patients who would reasonably require at least one review visit<br><br>Failure to refer eligible patients to chronic disease management service<br><br>Failure to refer eligible patients for rehabilitation (excluding geriatric rehabilitation)<br><br>Failure to arrange home assistance and community support to patients requiring assistance or deemed eligible on assessment by allied health professionals.<br><br>(For full definitions including examples, please refer to source)						
16	Quality of care delivered by general internists in US hospitals who graduated from foreign versus US medical schools: observational study		30-day mortality, 30-day readmission rates		cost of care (total part B spending for each hospital admission)			Patient characteristics (age in five year increments, sex, race or ethnic group, primary diagnosis defined by Medicare severity-diagnosis related group, Elixhauser comorbidity index with 27 coexisting conditions, median household income estimated from residential zip codes, and indicator for dual Medicare-Medicaid coverage, year indicators, day of the week), physician characteristics (age in five year increments, sex, patient volume), and hospital fixed effects (i.e., hospital specific indicator variables).

17	Evaluating Outcomes from an Integrated Health Service for Older Patients		acute length of stay, overall length of stay (including sub-acute), 30-day readmissions, mortality					
18	Relationship Between Quality of Care and Functional Decline in Hospitalized Vulnerable Elders	16 ACOVE Process-of-care quality indicators as previously reported by the same author in the publication at Index 11		ADL at discharge and 30 days after				Average VES-13 Score, Charlson Score, interaction between quality score and VES-13 score, number of baseline ADL limitations, Do-not-resuscitate/Do-not-intubate (DNR/DNI) status, log length of stay, number of Quality Indicators triggered, demographic characteristics (age, race, gender), and clustered for attending subject
19	Patient experiences and predictors in an acute geriatric ward: A cross-sectional study		(Note: while length of stay was measured, it was as a potential predictor of patient experience rather than an outcome measure)	Picker Patient Experience Questionnaire (PPE-15) (Note: while QoL was measured, it was as a potential predictor of patient experience rather than an outcome measure)				
20	Patient characteristics, resource use and outcomes associated with general internal medicine hospital care:		use of ICU, in-hospital death, 30-day readmission to general internal medicine service at one of the hospitals involved		Estimated average amount of hospital resources used for each hospital stay, including costs related to	Resource use: acute inpatient length of stay (total hospital length of stay - days spent at an alternate level of care), designated alternate level of care days, designated alternate	Annual number of hospital admissions, proportion of total hospital admissions that were general internal	

	the General Medicine Inpatient Initiative (GEMINI) retrospective cohort study				administration, staff, supplies, technology and equipment (excluding fee-for-service physician billing costs) using Canadian Institute for Health Information (CIHI) Resource Intensity Weight for each admission x annual cost per weighted case for acute inpatient cases for each hospital	level of care, number of bed days for the type of condition, use of advanced imaging (at least 1 of the various imaging tests/scans)	medicine admissions, proportion of ED admissions and proportion of total bed days that were general internal medicine. (To determine the proportion of hospital resources used by general IM patients)	
21	Implementation of a Physician Assistant/Hospitalist Service in an Academic Medical Centre: Impact on Efficiency and Patient Outcomes		in-hospital mortality, transfers to ICU, readmissions to the study hospital (within 72 hours, 14 days, and 30 days) (quality measures)	patient satisfaction as measured by responses to the Press-Ganey survey	total costs (efficiency measure)	length of stay (nearest hour) (efficiency measure)<br><br>effect on self-reported resident work hours		All adjusted models adjusted for clustering by attending physician. Adjusted for age, race, Charlson score, time of admission, insurer, and Case Mix Index (CMI).<br>Inpatient mortality: sex, race, Charlson score, CMI, and log of median income by zip code.<br>ICU transfers: age, sex, race, Charlson score, CMI, time of admission, and discharge to home or skilled nursing facility.

22	Impact of hospitalists on care outcomes in a large integrated health system in British Columbia		in-hospital mortality rate, 30-day readmission rate, average total length of stay, hospital standardised mortality ratio (actual over expected mortality)					age, gender and other factors
23	Similar Outcomes Among General Medicine Patients Discharged on Weekends		Adverse outcomes: subsequent unplanned readmission (to any public hospital, any reason) and/or mortality (within 2 days, 7 days and 30 days after discharge for processing)					Age, sex, CCI
24	Family Medicine Patients Have Shorter Length of Stay When Cared for on a Family Medicine Inpatient Service		total LOS at the study institution, 30-day readmission					age, gender, marital status, disposition location (home, skilled nursing facility, other), different admitting service, admission service (admitted by a different service), CCI, number of prior ED visits (previous 6 months), date of hospitalisation, dismissal diagnosis, number of prior hospitalisations (previous 12 months)
25	Does Doctor–Patient Communication Affect Patient Satisfaction with Hospital Care? Results of an Analysis with a Novel			A question from the Picker inpatient questionnaire during the 1-month follow-up survey to				Confidence in nurses, pain while in hospital, physical component subscale score of the 12-item Short Form Survey (SF-12), Charlson comorbidity score, demographic data (age, race, education level, marital status, insurance), intern who admitted the patient, month of admission

	Instrumental Variable			measure patients' ratings of their hospital care: "Overall, how would you rate the care you received at the hospital?" on a 5-point scale: Excellent, Very Good, Good, Fair, Poor.				
26	Relationship Between Quality of Care for Hospitalized Vulnerable Elders and Post-Discharge Mortality	16 ACOVE Process-of-care quality indicators as previously reported by the same author in the publication at Index 11	time to death after discharge (within 30 days, 60 days, 90 days and 1 year after discharge)					Markers of patient illness: age, VES-13 score, number of ADL limitations at baseline, Charlson comorbidity score, DNR/DNI status, length of hospital stay, number of quality indicators that the patient was eligible for which is the proxy for the number of geriatric-specific conditions<br>demographic factors: race, gender, marital status, age
27	Improving Patient Care Chronic Complex: Sub-Acute Care Unit		average length of stay, 30-day readmission rate, mortality rate, discharge to home, transfer to acute hospital, sent to socio-sanitari services	user satisfaction survey that assesses a global item				

28	Analysis of Casuistry and Results of the Implementation of a Care Programme in Ten Sub-Acute Care Units in Catalonia		average length of stay, readmissions after a month, mortality rate upon discharge, 6-month mortality rate, level of dependence (Barthel) upon release (and admission)					
29	Patient Outcomes in Teaching Versus Nonteaching General Internal Medicine Services: A Systematic Review and Meta- Analysis		inpatient mortality, 30-day readmission rate, length of stay		Inpatient/hospital costs (not a focus of the review)			age, Charlson comorbidity index, number of co-morbidities (some factors that some of the papers reviewed adjusted for. Heterogeneity in the analyses was reduced in the studies that made these adjustments, suggesting that these were major drivers of LOS and inpatient mortality in those studies)
30	Effects of an Acute Care for Elders Unit on Costs and 30-Day Readmissions		30-day readmissions to the same hospital, in-hospital mortality, discharge destination (to home, skilled nursing facility, other facility or inpatient rehabilitation/intermediate care)		Total variable direct costs, daily variable direct costs (total variable direct cost/LOS), total cost, daily total cost (total costs are sum of fixed direct costs, variable direct costs and indirect costs)	Total LOS (resource utilisation)		For variable direct cost: Age, sex, comorbidity score and case mix index

Appendix 5 Results of Sources of Evidence: Insights (Data Charted)

Index	Title	Insights
1	Association Between Treatment by Locum Tenens Internal Medicine Physicians and 30-Day Mortality Among Hospitalized Medicare Beneficiaries	Others: No significant differences in 30-day mortality rates among patients treated by locum tenens vs non–locum tenens physicians. Patients treated by locum tenens physicians had slightly higher Medicare Part B charges and lengths of stay, and lower 30-day readmission rates.
2	Challenges and opportunities in pragmatic implementation of a holistic hospital care model in Singapore: A mixed-method case study	Performance Management: Intra-hospital communication between physicians and nurses in making care plans is lacking. One reason for this is the lack of performance measurements for "proactive patient sharing".<br><br>Others: Compared to a specialist-led care model in another local hospital, the average length of stay was shorter, readmission rates were elevated, inflation-adjusted hospitalisation bill sizes were similar and inpatient mortality was lower. It was concluded that the generalist model may be able to produce comparable results, and can apply across a range of acuity levels
3	Comparison of Resident, Advanced Practice Clinician, and Hospitalist Teams in an Academic Medical Centre: Association with Clinical Outcomes and Resource Utilization	Others: Hospitalist teams discharged patients earlier and utilised fewer consultants but there were no significant differences in cost (total direct costs) or other clinical outcomes (LOS, 30-day readmission, inpatient mortality). The findings suggest that clinical outcomes are not significantly affected by inpatient team structure. Hence, general medicine inpatient APC or hospitalist teams are safe and efficient alternatives to traditional resident teams in an academic medical centre.
4	Development of Resident-Sensitive Quality Measures for Inpatient General Internal Medicine	Process measures:<br>Graduate Medical Education (GME) programs measuring clinical performance measures (CPMs) attributable to resident care would be valuable in various ways in determining readiness for graduation or predict performance after training. These include enhancing feedback to learners, public accountability, program evaluation, providing insight on whether the educational outcome connect with quality care. However many barriers to this have been identified, including lack of defined CPMs, difficulty in attributing care to individuals, variable case-mix indices, small sample sizes and data confidentiality issues.<br>RSQMs are defined as measures that are meaningful in patient care and mostly attributable to resident care. Once RSQMs are identified and given specific operational definitions, automation can be used for extracting and displaying the data. This would reduce resources needed to for sustained use of RSQMs. Compliance targets should be set below 100% to account for cases where resident fail to fulfill the measures for valid reasons such as unique patient situations or clinical context.<br>Respondents represented different years in residency training, different faculty clinical roles and faculty experience. They identified clinical conditions that are commonly managed on an inpatient GIM rotation for which RSQMs could be developed. 10-15 RSQMs for each condition was considered a manageable number that would allow resident performance profiles to be developed. Previous curricular reform work (faculty content experts selected skills common and observable on their rotation) guided the selection of conditions. Workplace-based assessment data was reviewed and six commonly assessed conditions were chosen. The "Inpatient GIM" category was used to capture measures not specific to the conditions or measures replicated across conditions indicating they were important across GIM inpatient care. Process and outcome measures had similar average group scores for criterion 1 of importance to clinical care, but the scores varied significantly for criterion 2 on representing a resident's care.<br>All final RSQMs were categorised as process measures, defined as "evidence-based elements of patient care that do not directly assess the patient's clinical condition". They consisted of half documentation tasks and half orders. 58% were most likely relating to a primary admitting diagnosis while 42% were potentially related to a primary admitting diagnosis or comorbidity distinct from the diagnosis but requiring management during hospitalisation. There are various reasons for why outcome measures produced in the nominal group technique (NGT) were not prioritized in the Delphi. Delphi groups were instructed to disregard

		feasibility when prioritizing the potential measures, but they may have known that final RSQMs would be likely extracted from EHR. This could have biased them towards process measures that are more easily retrieved like documentation and orders. Delphi members may also have thought residents to be the most responsible for process measures, while outcome measures are the collective result of multiple team members, system factors and patient factors. This hypothesis is supported by the post hoc analysis. However, there has also been some evidence that residents significantly contribute to outcomes.<br>It is unclear what the consequences are of using only process measures in resident assessment. It has been suggested that higher performance on process measures may translate to better outcomes. (e.g., improved process measure of use of a standard chronic obstructive pulmonary disease care bundle has been shown to improve outcome measures of length of stay and readmission rates). However, the association of process and outcomes is not always well-defined, depends on clinical context, evidence supporting the process measure, data sources, and other factors which are hard to measure. Despite that, process measures are valuable due to certain advantages. Operationalising process measures may be more straightforward than doing so for outcome measures. Process measures may also be more able to be attributed to individual physicians since there is a complex system that contributes to patient outcomes. Process measures are also important for formative organisational and individual efforts. RSQM data may promote personal and system-wide improvement. Process measures can be a starting point, leading to outcome measures as measurement and attribution techniques improve.<br>Performing well on these measures may not necessarily translate to benefits in patient outcomes. However, they are likely valuable for learner assessment, program evaluation and formative feedback. The authors also noted that they could be an initial step towards using outcome measures. Further steps suggested include formulating specific operational definitions to determine which RSQMs can be accurately measured with EHR data, testing the feasibility of operationalising the measures, gaining evidence of their validity to be used for resident assessment and to identify and validate outcome measures for these purposes. Further studies on whether the primacy of diagnosis affects resident contribution to outcomes will also be needed.<br>RSQMs reduce reliance on raters, which reduces the issue of rater bias, and supplements direct observation in assessing resident performance. Workplace-based assessment approaches usually have unwanted rater effects (e.g., variable frames of reference, high levels of inference, different methods for synthesizing judgments into numerical ratings, construct-irrelevant variance). The discriminatory potential of RSQMs across learners and patient encounters suggests promise for an assessment method that does not rely on raters. However, RSQMs need to be developed and empirically tested in other clinical care environments. The RSQMs may not generalise to other institutions. Other programs may also choose to create measures for other conditions common to GIM. Nonetheless, institutions can use these methods can be used to develop RSQMs that fit the local systems to measure resident performance for locally significant conditions.<br>Although the RSQMs developed were intended to be highly attributable to a single resident, there could be team effects affecting performance. When measuring individual performances in a dynamic team-based environment, such a “attribution/contribution challenge” will be present. However, being able to measure individual performance is important given that certification occurs on such a basis.<br>The RSQMs may not be equally valid for assessing residents regardless of whether they relate to the primary admission diagnosis or a chronic comorbidity. Resident contribution to care may be different in two situations. It is possible that for GIM inpatient teams, attendings focus more on primary diagnoses while residents manage chronic conditions so resident contribution may be larger for RSQMs relating to such conditions. Development of RSQMs in other specialties and settings will provide insights on what works for different providers and settings.<br>RSQMs that are discrete tasks rather than broad composite measures is also advantageous. Data on discrete tasks can provide specific formative feedback compared to composite measures that may lack specificity. Measures based on specific tasks also allows measures to be combined into various composite measures. This allows learners to be assessed through various lenses. (e.g., condition-specific composite measures can provide information on performance for the specific condition, or combined to form composite measures for processes like admission, discharge and medication ordering across conditions)
5	Investigating the effectiveness of care delivery at an acute geriatric community hospital for older adults in the Netherlands: a protocol for a prospective controlled observational study	-

6	A novel organizational model to face the challenge of multimorbid elderly patients in an internal medicine setting: a case study from Parma Hospital, Italy	Outcome measures: Data on inhospital adverse events and negative consequences of hospitalization including decline in physical performance, cognitive impairment, delirium, malnutrition and polypharmacy are not usually collected in administrative reports and hence were lacking for the analysis. The accuracy of data from regional administrative databases depends on the Hospital Discharge Record compilation by hospital physicians. Although improvement in administrative performance indexes does not necessitate direct positive patient outcomes, there are theoretical advantages like how shorter length of stay can provide psychosocial and clinical benefits. Short-term hospital readmissions are strongly related to baseline conditions and clinical course and independent of LOS and organization of care. Interventions to reduce LOS in geriatric settings are not associated with higher readmission rates.
7	Selecting best-suited "patient-related outcomes" in older people admitted to an acute geriatric or emergency frailty unit and applying quality improvement research to improve patient care	Outcome measures: Patient-related outcomes should determine wider impact and explore an issue from the service user and stakeholder perspectives but keep the focus on research and QI. Outcomes should be measured at different levels: patient (direct clinical outcomes); staff; family and carers; and community and organization (indirect clinical outcomes). Clinical outcome data can be used to benchmark any acute geriatric service and compare against national standards. Clinical outcomes must be interpreted with appropriate patient characteristics. Common demographic markers are routinely available. The patients can also be described based on Charlson's comorbidity index, history of cognitive impairment or dementia, polypharmacy, frailty using Fried's phenotype and Rockwood deficit model, Clinical frailty scale, Frailty Index, activities of daily living using validated scales like the Barthel scale, number of hospital admissions in last 12 months, range of formal or informal carer support, occasions of getting out of the house with or without assistance, acute illness severity at admission (Modified Early Warning Score), reason for admission, single-organ or multisystem failure, functional decline from baseline or 2 weeks prior to admission, nutritional status, skin breakdown, immobility or falls, incontinence, presence of delirium and dementia. This is important for comparing outcome data across AGUs. Staff-related outcomes should be measured for comprehensive evaluation of the clinical outcomes due to the associations between staffing and patient-related outcome. Caregiver outcomes are a type of patient-related outcome and should be included as part of an evaluation of an AGU. Caregiver burden affects the caregiver's health and is associated with adverse clinical outcomes for the elderly being cared for. PREMs are increasingly used to obtain data on patients' perceptions of their health and experiences while receiving care, and could be helpful to improve quality of care. PROMs are largely used in research but not yet part of routine clinical practice to improve quality of care although patients' views should be taken into account in this. Routine use of PROMs improves decision-making between doctors and patients and improves patient care. Patients' views on satisfaction with their care are important for improving the care experience. Quality of patient care is defined in terms of effectiveness of the care, patient safety, and patient experience. Patient-reported outcomes are directly reported by patients based on their experience, without interpretation. Patient related outcomes (PROs) define the patient's QoL and the impact on their health and functional status following care. Patient-reported outcome measures are validated tools to measure PROs and provide qualitative or quantitative data. They include tools to define functional status or health-related QoL, symptoms, or disease-specific burden. They help in the evaluation of service models or care providers. Patient-related outcomes have associations with the success of an organization and affect the organization's performance, strategic model, and vision. For a complete evaluation of an acute geriatric service model, the impact on organizational-level outcomes should be measured. The best-suited measures from a large range of possible direct and indirect patient-related clinical outcomes should be selected for service delivery. Clinical outcome data should be regularly evaluated to benchmark services for acute elderly patients. Standardized and validated tools should be used to measure indirect clinical outcomes like caregiver burden, and organisational-related outcomes.<br><br>Performance Management: The paper included a "brief guide to medical statistics". "The aim should be to have benchmark baseline data and compare against those benchmark standards. Data should be presented in such a way that they do not lose their evidence and content. The best approach is to present data in terms of timeline series so that data do not mislead the user". "A study sample for a service over a predefined time period should be used to describe population and benchmark service outcomes. Therefore, ensure that the study design includes a good subset of the population which is a close representation of the population sample." The paper also provided insights on a quality improvement methodology, which is different from performance management, although related.
8	Variation in physician spending and association with patient outcomes	Outcome measures: Focused on Medicare Part B spending because it encompasses services at physicians' discretion, is conditional upon admission, conditional on admission and is also a proxy for the intensity of resource use of physicians which may be incorporated in Part A spending. Inpatient medications, laboratory tests and imaging are in fixed Part A payment, so the study could not measure how use of these varies across physicians. However, the large differences in Part B spending between physicians in the same hospital likely translate to resource use in the Part A payment. 30-day mortality and readmission rates are "widely accepted standard measures of quality of hospital care". However, this study did not measure other aspects of quality like patient experience. To conduct the analysis, they first quantified the proportion of variation in Part B spending attributable to hospitals, physicians and patients then analysed the association between physician spending and outcomes. To ensure patient illness severity did not directly affect physician spending estimates, they calculated physicians' spending from different years than those the outcomes are from.

		Performance Management: Under the Medicare Access and Children's Health Insurance Program (CHIP) Reauthorization Act (MACRA), most physicians will be measured and compensated on the basis of performance, which encompasses cost and quality of care. Data on the relation between physician spending and quality of care or patient outcomes is limited. However, it was found that within the same hospital, physician spending varies significantly and higher physician spending is not associated with better outcomes (30-day mortality, readmissions) of hospitalized patients. Health care spending across individual physicians varies more than across hospitals. Higher spending physicians were slightly older and more likely female. Individual physicians are gaining recognition as key contributors to observed variation in healthcare services. Both physicians and hospitals, which are both "units of observation where key decisions are made"), are important for the efficiency of health care and policy efforts should focus on these. However, policies to improve the quality and value of inpatient care (e.g., hospital value-based purchasing, penalties for 30-day readmissions) so far have focused on hospitals. It is assumed that hospitals can affect behaviour of individual physicians. Targeting both individual physicians' practice patterns and hospitals may be more effective in reducing excessive spending than focusing solely on hospitals. Higher-spending physicians may be able to reduce resource use without compromising patient outcomes. Policies targeting physicians within hospitals (e.g., physician-level pay-for-performance programs and reporting comparisons of resource use among physicians in the same hospital) should be developed and evaluated. As the analysis was restricted to the hospitalised Medicare population with medical conditions, findings may not apply to non-Medicare populations.
9	Census of Ligurian Internal Medicine Wards of non-teaching hospitals	Performance Management: The study did not include Internal Medicine Wards of university hospital as most are sub-specialised, their admissions are often programmed and not from the Emergency Room, hence benchmarking would be inconsistent. The authors expect internists to be included in working groups about quality and expenditure, technologies, reorganization and interplay between hospital and community settings, with resource optimisation and constant evaluation of the cost/benefit ratio. The mean DRG-weight did not really reflect the resource intensity of the wards. The 2 wards with a separate costs centre for high dependence areas, they reached a higher mean DRG weight. This could be due to suboptimal codifying of diagnoses and internist education on this would be important.
10	Pocket Change: A Simple Educational Intervention Increases Hospitalist Documentation of Comorbidities and Improves Hospital Quality Performance Measures	Performance Management: Hospitalists are directly affected by payment reform, which is highly dependent on documentation. Accurate and complete documentation of patient comorbidities is important for clinical care, quality performance measures and hospital reimbursement especially given developments in that aspect. After the replacement of diagnosis-related groups (DRGs) with Medicare Severity DRGs (MS-DRGs), higher reimbursement is given based on presence of documented complications or comorbidities. The Hospital Value-Based Purchasing program ties hospital reimbursement to improvement in many quality measures which depend on complete medical documentation. The Physician Value-Based Payment Modifier examines quality at the physician level and will affect Medicare Part B physician payments. Reported outcome measures, such as expected mortality, are determined using coded diagnoses and MS-DRGs hence the medical record is critical in determining hospital quality rankings and influencing perceived quality of care. Clinical documentation improvement (CDI) programs are increasingly important to maximize hospital reimbursement. However, they require hospital resources and are likely less efficient at capturing relevant comorbidities. The pocket card reminder optimises physician documentation at point of creation and increases efficiency. It can also improve documentation for all inpatients unlike CDI programs which usually focus on selected groups. Regular feedback provided to the hospitalists was important for this intervention to succeed. The intervention appears complementary to CDI programmes. However, the results may not be generalisable to other academic medical centres or internal medicine trainees, although other evidence supports that pocket card interventions for IM residents can improve physician documentation as seen in the case-mix index.<br>Hospitalists may not recognise their central role in hospital performance and reimbursement. It may be useful to emphasise that complete documentation of patient comorbidities demonstrates true acuity of their patients, and affects the mortality scores, severity of illness scores, and quality rankings that are often reported at the physician level and will be tied to reimbursement in the future. Ongoing physician education and re-evaluation of "high yield" comorbidities will be necessary to maintain the documentation quality.<br>A simple and inexpensive educational intervention and pocket card reminder for hospitalists in an academic medical centre was associated with improved documentation of patient comorbidities present on admission, above that of a CDI programme. The hospitalists felt they were making a difference in the hospital's quality measures. These improvements in documentation were reflected in higher expected LOS compared to baseline. It could be useful for documenting "high yield" comorbidities present on admission in a broad range of patients. It does not greatly interrupt physician workflow. Out of the top 10 comorbidities identified that have significant effects on expected mortality, expected LOS, and expected direct cost among the highest volume discharges by MS-DRG for internal medicine inpatients at the University of Washington Medical Centre (UWMC), 6 "high yield" comorbidities that appeared to have low baseline rates of documentation in the medical record by attending physicians were chosen for the intervention.

11	Using assessing care of vulnerable elders quality indicators to measure quality of hospital care for vulnerable elders	Process measures:<br>There was significant variation in the quality-of-care processes across several domains of care for hospitalized vulnerable elders on a general medical service setting. Quality assessment of conditions prevalent in vulnerable elders is significantly worse than for general hospital care. QIs for general medical care had a much higher adherence rate than pressure ulcer care, delirium and dementia care. According to standard nursing assessment forms, nurses were responsible for high rates of adherence to some screening indicators, although in patients with functional limitations, nurse admission assessments of functional limitations often disagreed with what was reported by patients on admission. Although medical professionals consistently perform some basic geriatric evaluations in the hospital setting (e.g., screening for functional impairment, pressure ulcer risk), higher-order skills involving evaluation and treatment of conditions prevalent in vulnerable elders is incomplete. Screening for cognitive function using formal instruments is low. Findings suggest that although patients are screened for certain conditions, they do not receive the care indicated by the screenings.<br>Adherence to geriatric-specific QIs was found to be lower than adherence to general hospital care QIs. Nurses often screen for geriatric conditions using standard nursing assessments. Hospital care QIs that focus on screening may overestimate performance by detecting standard nursing or protocol-driven care. Further work should focus on education of hospital staff to perform accurate assessments and follow-through with diagnosis, treatment, and documentation of geriatric-prevalent conditions.<br>The results suggest that it is important to include geriatric-prevalent conditions in measures of quality for older hospitalized patients in addition to the traditionally studied general medical conditions (e.g., heart failure and diabetes mellitus). There is also a need for more in-depth training for medical professionals in the hospital care of older patients, with continued education to develop and maintain skills<br>The results have important implications for the use of ACOVE QIs and chart abstraction in hospital-level performance management. Firstly, measures that rely on chart documentation of screening but do not measure accuracy or follow-up of these screenings may overestimate performance. Secondly, "because high adherence rates for screenings do not correspond to high rates of diagnosis and treatment and their documentation, more focus should be placed on evaluating diagnosis and treatment in quality-of-care measurement systems". Thirdly, although quality measures traditionally focus on physician performance, the results demonstrate the importance of nurses in providing quality care for patients, especially in the hospital. "Their primary role in screenings and assessment makes nurses frontline agents of care processes". Nursing care should be included in performance measurement and improvement programs that strive to improve quality of care for geriatric patients.<br>Data used relied solely on information in the written medical record, which is known to underestimate the quality of care in outpatient settings. Lack of documentation of performance does not necessarily mean care was not performed, although poor documentation and poor process of care has been correlate as documentation has an important role in communication within the care team.
12	Two European Examples of Acute Geriatric Units Located Outside of a General Hospital for Older Adults With Exacerbated Chronic Conditions	-
13	Short-term Resource Utilization and Cost-Effectiveness of Comprehensive Geriatric Assessment in Acute Hospital Care for Severely Frail Elderly Patients	Performance Management: The hospital care cost contributed the majority of the total cost per patient, while primary care constituted a minor part of the health care utilisation and costs. This may resemble the distribution of costs of care for frail elderly patients with a heavy morbidity burden. This may reflect vulnerability and high probability of recurrent acute disease.<br><br>Others: The cost-effectiveness analysis only used quality-adjusted life years (QALY) and cost, not mortality or HRQoL directly. Mortality was taken into account for QALY calculation. For cost effectiveness analysis deaths were included
14	RECALMIN: The association between management of Spanish National Health Service Internal Medical Units and health outcomes	Performance Management and Outcome measures:<br>The study analyses clinical outcomes, taking into account patient-level and hospital level factors. Ambulatory healthcare may also play a role.<br>Defining the performance of a medical unit can involve multiple disciplines and approaches, with variables beyond those of this study.<br>In IMUs of the Spanish National Health Service (SNHS), no association was found between complexity and intra-hospital risk-adjusted mortality, except for acute myocardial infarction (AMI). Nurse workload, presence of a safety committee and multidisciplinary ward rounds is weakly but significantly associated with

		mortality and length of stay. Better disease specific outcomes adjustments and a larger IMU sample could reveal more insights on the association between management of the IMUs and healthcare outcomes.<br>The "homogeneous quality of care of UMIs among different complexity of hospitals" could be partially due to the long residence program to specialize in IM in Spain and a lower dependence of complex technology of IMUs than other medical specialties. In a separate review, there were no outcome differences found between IMUs in teaching and non-teaching hospitals. However, better predictive risk-adjustments may uncover significant differences.<br>The presence of these management practices are loosely associated with shorter lengths of stays and lower mortality rates. However, the study could not find an independent effect of management variables on health outcomes when introduced as risk factors in regression models. Management in the IMUs is intertwined with other variables (structural, management and complexity variables) in the hospitals. Additionally, the sample had a limited number of IMUs and the predictive value of the risk adjustments for the global case-mix was relatively low. These reasons may explain the weaker evidence of the association between management and outcomes. Better disease-specific adjustments and a larger sample of IMUs could provide additional insight regarding the association.<br>There were no significant discrepancies found between the data provided by the IMUs and the objective data from the Minimum Basic Data Set (MBDS). Outcome indicators are based on administrative records. Comparisons to medical record data has helped to validate the use of administrative records to estimate outcomes of health services. Research on health service outcomes has also used administrative records to estimate these outcomes. MBDS data is subject to auditing and provides valid information. "The reliability of studies of this type enables the public comparison of hospitals in terms of their outcomes".
15	Quality of care factors associated with unplanned readmissions of older medical patients: a case-control study	Outcome measures, process measures and performance management: There were preventable quality of care factors involving 44% of readmitted patients compared to 12% of matched control. Deficiencies in advance care planning, management of presenting clinical condition, assessment of functional limitations and initial diagnostic evaluation were the most common problems among readmitted patients. Among controls, lack of discharge communication was the most frequent, which may be due to medical staff perceiving them to have a lower need for early post-discharge care from community providers due to fewer comorbidities and past hospitalisations, hence less requirement for discharge documentation. Absence of advance care plans was the most frequent quality of care factor, in 16% of readmitted cases. Unplanned readmissions of older medical patients appear associated with more quality of care problems during index admission compared to a similar cohort of non-readmitted patients. Rectifying these issues may reduce readmission risk, although the extent is unclear. The propensity for readmission of older, frail patients with complex needs and advanced chronic disease will remain high despite optimal care. Excessive, penalty-driven efforts to reduce readmissions in this highly vulnerable group may have unintended adverse outcomes. However, a third of the readmissions in this cohort which occurred within a week post-discharge, are more likely to reflect deficiencies in peridischarge care. More distant readmissions likely reflect underlying disease severity instead. Preventing at least a third of the potentially avoidable admissions would lower the overall readmission rate by 15%, which is a basis to consider strategies to improve quality of peridischarge care. This study identified quality of care factors associated with readmissions, but a causal relationship that implies that these readmissions were probably avoidable cannot be inferred. Representative sampling of readmissions and controls based on prevalence of discharge diagnoses and close matching of both groups minimized potential bias in overestimating quality factors in readmitted cases due to older age, greater comorbidity or disease severity. A case-control design allowed readmitted and non-readmitted patients to be differentiated in the types and frequency of quality factors. The low prevalence of quality factors (apart from discharge communication) among controls compared to readmitted cases may reflect those biases specific to assessment of readmitted cases. The usage of the "eyeball" test of quality may have caused under-calling of more subtle quality factors among controls, although regularly monitored quality indicators of the study service suggest a high performance level.
16	Quality of care delivered by general internists in US hospitals who graduated from foreign versus US medical schools: observational study	-
17	Evaluating Outcomes from an Integrated Health Service for Older Patients	Outcomes and Performance Management: Patients in both groups had similar health outcomes despite the OPERA patients having a poorer health status. This could be due to the difference in allied health visits, general physician skill and experience, or the overall approach associated with CGAs. Maintenance of health outcomes although OPERA patients were more ill shows the need for such CGA models.<br>The CGA approach is still evolving, and does not have widespread standardisation. Collaborating with other unit on best practices may lead to more standardised methods of care that could potentially decrease costs, improve functional status and improve patient outcomes.

18	Relationship Between Quality of Care and Functional Decline in Hospitalized Vulnerable Elders	Process measures, Outcome measures and performance management:<br>The ACOVE quality indicators were developed to provide an objective standard of measuring indicated processes of care, in order to assess quality of care for older patients. They were developed using the modified Delphi method and are a series of IF/THEN statements focusing on clinical care processes that should occur for eligible patients. Previous research has found that higher quality care (measured by composite score of adherence to ACOVE QIs) is associated with improved three-year survival in community dwelling vulnerable elderly.<br>Hospitalised older patients are at increased risk of functional decline post-discharge. The etiology of functional decline of hospitalised patients is multi-factorial, but several factors involved (e.g., immobility, poor nutrition, inadequate pain control and delirium) are care processes addressed by ACOVE QIs. It was found that increased quality of care (measured by the composite score of adherence to ACOVE process of care measures) is not significantly associated with reduced risk of functional decline in hospitalised vulnerable elderly patients. Notably, patients with a documented effort to improve mobility were more likely to experience functional decline post-discharge. Patients who received an assessment of nutritional status were less likely to experience functional decline. Quality was measured using a small subset of QIs. Adherence to certain quality indicators may also be associated with positive outcomes other than functional decline but this was not measured. (e.g., cognitive screening could be associated with reduction in delirium). The study looked at the contribution of early discharge planning and initial of physical therapy in hospital, however, immediate post hospitalisation and rehabilitation settings may be a better predictor of functional decline for hospitalised seniors. However, these were not examined. Chart documentation has also been shown to underestimate the care given to patients. Hence, patients may have received higher quality care than documented in their charts. The definition of functional decline that was used (the emergence of a deficit in a single ADL) could be subject to natural fluctuation. However, measures of catastrophic functional decline were included to account for this. This study highlights the difficulty of assessing process-outcome causal relationships in observational study designs. An instrumental variable approach was considered, but the researchers were failed to identify a potential variable that would relate to whether patients would receive certain quality indicators. Future research should have more robust measurement and controlling of covariates, with a focus on possible selection effects. To rigorously evaluate the effect of quality of care, randomised controlled trials that compare usual and “high quality care” are likely necessary. Recently, there has been significant research to identify relevant valid measures of quality of healthcare. It is important to develop valid measures of quality given the increasing focus on measuring and rewarding adherence to quality measures through the increasing use of pay-for-performance and public reporting programs. Most such programs use quality measures that focus on processes of care that can be precisely specified. Importantly, process of care measures do not require the necessary adjusting for disease severity that patient outcome measures (e.g. mortality) do. Other than measuring individual care processes, there has been a recent focus on the need for composite measures of quality that take into account the multiple care processes indicated in medically complex patients. Given the cost of measuring and improving quality of care, adherence to such measures would ideally improve patient outcomes.
19	Patient experiences and predictors in an acute geriatric ward: A cross-sectional study	Performance Management: Older people tend to face issues in their healthcare particularly related to continuity and discharge. This highlights the need for protected time for healthcare personnel in the discharge process to ensure patients and/or their relatives receive the information needed before discharge. The findings of this study may be used in systematic quality improvement work for these patients. Targeting patients with comorbidities and thematic areas of patient-healthcare professional communication and discharge processes may help improve the experiences of older patients.<br>Outcome measures:<br>Comorbidity had a weak, but statistically significant, influence on PPE-15 problems. Comorbidity is one of the factors commonly used to indicate vulnerability. The findings support that vulnerable patients have reported significantly less positive care experiences than non-vulnerable patients.<br>Patients self-reporting their experiences at home after discharge may have increased the risk of recall bias.
20	Patient characteristics, resource use and outcomes associated with general internal medicine hospital care: the General Medicine Inpatient Initiative (GEMINI) retrospective cohort study	Performance Management:<br>Patients admitted to general internal medicine are often characterized as older (median age of 73 in this study) and highly multimorbid (median of 6 coexisting conditions in this study)<br>General internal medicine patients are highly variable in their individual characteristics, the conditions leading to hospital admission and resource use and outcomes within each condition. Reasons for such variability should be a key research focus for this patient group, which is poorly represented by averages or other measures of central tendency.<br>There was a large, growing population of patients admitted to general internal medicine services which consumed significant hospital resources. They are characterized by marked heterogeneity. There are substantial opportunities to develop measures of quality of care for general internal medicine patients, to study variations in care and outcomes, and improve quality of care. Electronic data collection and linkage can support multicentre research to study this complex population. A better understanding of general internal medicine patients is crucial for a quality, sustainable health care system.

21	Implementation of a Physician Assistant/Hospitalist Service in an Academic Medical Centre: Impact on Efficiency and Patient Outcomes	Others: A service staffed by hospitalists and physician assistants is a safe alternative with comparable efficiency to house staff services, for general medicine inpatients admitted to an academic medical centre. Self-reported resident duty hours, patient satisfaction, rates of hospital readmissions, transfer to ICU or inpatient mortality was similar. LOS and total costs of care were only marginally different.
22	Impact of hospitalists on care outcomes in a large integrated health system in British Columbia	Outcome measures: Hospitalist care is associated with lower mortality and readmission rates, although LOS was similar to Family Physicians (FPs). It is also associated with lower mortality than IM providers. However, these differences were not present consistently when patients with specific diagnoses were compared. This could be in part due to the relationship between caseload and outcomes. Other factors could also be important. For example, patients admitted by IM providers spend much more time in specialized units. They largely present with cardiac conditions and hence may have higher acuity levels, requiring more invasive interventions. When patients with specific clinical diagnoses were studied, higher readmission odds only continued to be found for those hospitalised for pneumonias. Hence, hospital readmission rate is a complex measure that could be influenced by various hospital and community factors. It may also vary for patients who present with different clinical diagnoses. Further research is needed for a clearer understanding of the relationship between provider type and experience with hospital readmission for patients with various clinical presentations.<br>Performance Management: The IM provider group is heterogenous with various subspecialties (cardiologists, gastroenterologists, general internists) grouped under the IM category in the database. Hence, comparisons between the IM provider group and the other provider groups which are more homogenous must be interpreted carefully.
23	Similar Outcomes Among General Medicine Patients Discharged on Weekends	Performance Management: It was found that the proportion of patients discharged on weekends is lower than the proportion admitted on weekends. It was also found that lower risk patients seem to be preferentially discharged on weekends. Consequently, post-discharge outcomes are similar between weekend and weekday discharges despite weekend discharges having a shorter LOS and less availability of outpatient resources.<br>Outcome measures: It is noted that death or readmission soon after discharge does not necessarily indicate suboptimal quality of care during the preceding hospitalisation or that the deaths or readmissions were potentially preventable. Post-discharge mortality or readmissions are affected by various factors other than quality of inpatient care. Further study should be conducted on the reasons that more complicated patients are not discharged on weekends, as safely increasing weekend discharge rates could improve safety and efficiency. (by reducing unnecessary exposure to in-hospital adverse events such as falls, unnecessary urinary catheterizations, and healthcare-acquired infections). The study did not cover physician decision making however the authors suggest that based on their results, physicians incorporate discharge day into their discharge decision making and may be selecting younger patients with less comorbidities for weekend discharges or delaying the discharges of older patients with more comorbidities to weekdays. The authors also noted that although post-discharge follow up may be associated with better outcomes as suggested by previous literature, they could not adjust for patterns of outpatient follow-up in the study.<br>There may be concerns that LOS could be a "mediator in the causal pathway between discharge decision and post-discharge events, and that adjusting for LOS in analyses could then spuriously obscure a true association". However, the authors highlighted that the "2 sensitivity analyses to explore this (the 1 in which we excluded LOS from the analyses and the 1 in which we included expected LOS rather than the actual LOS) revealed nearly identical point estimates and 95% CI as our main analysis."
24	Family Medicine Patients Have Shorter Length of Stay When Cared for on a Family Medicine Inpatient Service	Outcome measures: Continuity of care, more intimate knowledge of outpatient resources available to assist with transitions of care, and potential additional unadjusted complexity of patients on Hospital Internal Medicine (HIM) services likely contribute to shorter LOS for Family medicine inpatient service patients compared to general medicine inpatient services.

25	Does Doctor–Patient Communication Affect Patient Satisfaction with Hospital Care? Results of an Analysis with a Novel Instrumental Variable	Performance management/outcome measures:<br>There was considerable variation in patients' perceptions of physicians' communication skills in the hospital. In ordinary least squares regression, overall satisfaction was highly correlated with overall ratings of attendings' communication behaviours, with an increase in overall satisfaction of 0.58 points on a 5-point scale for each 1-point increase in overall attendings' communication behaviours. The coefficient size, 0.4-0.68 points on a 5-point scale in meaningful in quality of care ratings. Small differences (e.g., 0.1) in patients' quality of care and satisfaction ratings have been associated with changes in patient behaviour and health outcomes in other literature.<br>The relationship may be confounded by patient-level factors, but after controlling for those, there is still evidence of a statistically significant relationship. An example of how the association can be confounded would be that an association between ratings of communication behaviours and overall satisfaction could reflect reverse causation, where patients who are more satisfied are also more likely to rate their physicians' communication behaviours highly. Patients who receive good news or good health outcomes may also rate the communication behaviours highly and report greater satisfaction. However, the association would not be due to any effect of communication on overall satisfaction. Similarly, patients who are harder to please may give low ratings to both measures. Hence, ratings of communication that are independent of individual patient factors that may also affect overall satisfaction are necessary. Instrumental variable analysis can be used to address these confounder issues. The authors designed a novel measure of the communication behaviours of the physician that is independent of patient-specific factors that may affect overall satisfaction such that it could be an instrumental variable in studying the effects of communication on overall satisfaction. This variable was created using the average ratings of physicians' communication behaviours provided by other patients cared for by that physician. While patient-specific factors (like patient outcomes) affecting attending communication ratings could bias the assessments of the attending's communication style, other patients' ratings of that attending would not be confounded in that manner. The approach of using IVs constructed using the assessment of other patients "may be useful for addressing other potential associations of physician characteristics with outcomes in situations in which analyses using ratings from a single patient could produce spurious associations due to confounding by patient-level factors"<br><br>Outcome measures:<br>Patient satisfaction is important as it captures the patients' experience of healthcare beyond direct effects on health and recognises their role as a partner in healthcare, reflecting the patient centredness of care. It also provides insights into patients' perceptions of interpersonal relations and amenities. Considerable resources have been channelled towards the goal of patient satisfaction. There has been evidence that the quality of aspects of communication with physicians is important to inpatients. In the outpatient setting, physicians' communication behaviours are important in contributing to patient satisfaction.<br>Nursing care, report of pain while in hospital, level of health, race, age, gender and socioeconomic level are known to affect patient's ratings of hospital care. These variables were controlled for.<br><br>However, the study could not determine if other physician characteristics like technical behaviour may have affected patient ratings of physician's communication behaviours and ratings of care received. Additionally "patient satisfaction surveys that request ratings are inherently confounded by expectations, and use of "experience" or "experience-like" questions, such as "would you recommend this hospital to a friend or relative" might minimise this confounding."<br>There was a bias in the sample in that patients not followed up in the one-month survey were more likely to have public assistance for medical coverage and to be male and African American which may artifactually increase the estimation of patient's ratings of the communication behaviours as the last 2 characteristics have been associated with lower such ratings.<br>The response rate of 75% of eligible discharged patients was similar to typical response rates of hospital satisfaction surveys.<br>The results may be relevant only among subjects who respond to patient satisfaction surveys. Nonetheless, it is "an interesting group to understand to the extent that the type of bias our analysis can adjust for (e.g., bad outcomes affect ratings) is one that we would want to control for in analyses of answers from these respondents."<br>Generalisability of the findings may be limited as the samples were from one service at one hospital. It focused on an urban inner-city population which has not been well represented in previous studies of patient-physician communication and quality of care<br><br>Performance Management:<br>The association of physicians' communication behaviours and overall satisfaction ratings suggests that simple changes physicians can implement when communicating with patients can have a substantial impact on patients' quality of care ratings. While most physicians were found to be doing a "very good" job, there is significant room for improvement and this could be an important area for hospitals to focus on in improving quality of care. Further data collection efforts

		on patient ratings of physician communication behaviours and satisfaction will be available from the Hospital Consumer Assessment of Healthcare Providers and Systems and allow important further study. Practice environments need to be shaped to allow the development of good patient-physician relationships.
26	Relationship Between Quality of Care for Hospitalized Vulnerable Elders and Post-Discharge Mortality	Process measures:<br>ACOVE quality indicators were previously developed as standardised geriatric-specific quality measures to assess quality of care for older patients. Previous research with ACOVE measures showed that geriatric specific quality measures have a much lower adherence rate than general medicine quality indicators. A composite quality score was used to determine adherence to ACOVE QIs. This was derived for each patient from the percentage of QIs triggered that were met, using only QIs for which the patient was eligible.<br>Previous research showed that chart abstraction underestimates true quality of care. Hence the quality scores calculated may not accurately represent the quality of care received by the patient.<br><br>Outcome measures:<br>Most of the demographic covariates showed no significant relationships with mortality, except a significant relationship that females were 49% more likely to die within 1 year than males. Sicker patients and patients with DNR/DNI orders were also more likely to die within 1 year<br>This study evaluates mortality as a patient outcome, but it may not be the most relevant outcome for older patients, who often value quality of life over length.<br><br>Relation between process and outcome measures:<br>Patient illness and physical function can significantly affect patient outcomes regardless of quality of care. Hence, markers of patient illness were used to adjust for this.<br>Patients whose LOS was one day or less were excluded, among other exclusion criteria, as quality of care would have less impact on outcomes in such a short period. Patients who were transferred from ICU to GM inpatient service were also excluded due to their severity of disease and potential for adverse outcomes unrelated to quality of care in the GM service.<br>After controlling for sociodemographic and disease severity variables, higher quality of care for hospitalised vulnerable elders, as measured by ACOVE measures, appeared to be associated with a lower risk of death at one year post-discharge. Every 10% increase in quality score corresponded to patients being 7% less likely to die. Other research has found that the most significant relationship between quality of care and mortality occurred at a delayed (>500 days) rather than immediate time frame. Patients that received a nutritional status assessment were less likely to die one year after discharge. Out of the six universally triggered specific QIs, only nutritional status assessment had a significant relationship with post-discharge mortality, being associated with a 39% decrease in patient mortality a year after discharge.<br>The results support the initiation of future work testing interventions to improve adherence to these quality indicators.<br>A possible reason for the association is that better quality of care directly improves patient survival. This would be a strong reason to improve care processes for hospitalised seniors that are emphasised in ACOVE QIs. However, it is also possible that hospitalised older patients who have longer life expectancies receive higher quality care. This could occur if physicians preferentially provide certain care processes (e.g. nutritional status assessment, mobility plans) to those who are expected to live longer. However, there has been evidence to suggest that physicians do not accurately predict patient prognosis during hospitalisation and provide aggressive care to all except those with a priori preferences for less aggressive care, and that sicker patients tend to receive more care. Similarly, this study showed that patients who receive higher than median quality scores were those who triggered more QIs, hence had more geriatric conditions, and had longer LOS.<br>Another explanation could be that the receipt of certain care processes is associated with some variable related to improved survival. For instance, adherence to nutritional status was highly correlated with the overall quality score. Adherence to cognitive screening, functional screening and early discharge planning were associated with nutritional status assessment, demonstrating that patients who received nutritional status assessment were more likely to receive higher quality of

		care. Nutritional status documentation is also likely correlated with receiving nutritional supplementation, which could lead to improved outcomes, although it is unclear if it would improve survival.<br>Other unmeasured factors could also be causing the impact on mortality. Causality cannot be assumed but further research on the mechanisms of this relationship and to establish that causal relationship is needed, given the potential impact on mortality.<br>Since the study was done at one institution with a largely African American female patient population, the results may not be generalisable. There was also little inclusion of patients with cognitive impairment and their proxies. Nonresponders patients may also differ from the study group.
27	Improving Patient Care Chronic Complex: Sub-Acute Care Unit	Outcome measures and performance management: A satisfaction survey was used to assess patient satisfaction. This was found to be very high, which the authors linked to indicating the quality of care. Management parameters of the unit showed correct internal functioning and good coordination with the chronic functional unit of primary care and hospital emergency services. (The specific indicators measured were not elaborated on.)
28	Analysis of Casuistry and Results of the Implementation of a Care Programme in Ten Sub-Acute Care Units in Catalonia	-
29	Patient Outcomes in Teaching Versus Nonteaching General Internal Medicine Services: A Systematic Review and Meta- Analysis	Performance Management: The study did not find evidence for substantive differences in outcomes for GIM patients based on admission to a teaching or non-teaching service. They suggest that penetration of health information technologies, performance measurement and incentives or penalties, patient volumes and case-mix, and unit staffing ratios are more important to the delivery of high-quality care and good outcomes than the presence of faculty physicians and medical trainees. Since teaching hospitals are important in training future providers and hospitals involved in research deliver better quality care and outcomes, these results should lessen concerns teaching hospital administrators may have about maintaining or introducing GIM teaching units in light of initiatives to financially penalise hospitals with higher-than-predicted LOS or 30-day readmission rates.<br>Others: Most studies used administrative data, which precludes fully adjusting for severity of disease or functional status
30	Effects of an Acute Care for Elders Unit on Costs and 30-Day Readmissions	Outcome measures:<br>The hospital administrative database was used to obtain patients' financial information. The data included payer source, hospital charge, cost, allocated bad debt, and net revenue for each patient. "The [hospital administrative database, Transition Systems Incorporated (TSI)] system generates each patient's total costs from the sum of fixed direct costs, variable direct costs, and indirect costs. Fixed direct costs are those associated with patients that cannot be linked to a specific patient (e.g., the salary of the unit nurse manager). Variable direct costs are attributed to direct patient care (e.g., laboratory tests, medications)." "Indirect costs cannot be attributed to a specific patient or department (e.g., utilities, salaries of hospital accountants)."<br>The primary outcome measure was total variable direct costs which was used because it represents "the cost stemming directly from daily patient care and would therefore provide the best measurement for the effect of the ACE model on costs". Also, fixed and variable staffing was identical across the ACE and Usual Care unit.<br>Geriatric syndrome information in the hospital database/medical record was lacking. It did not record place of residence before hospitalisation (where they were admitted from) and did not distinguish between long-term care and sub-acute rehabilitation for their discharge destinations, which limits the analysis. They also could not determine the number of 30-day readmissions to other hospitals.<br>The article focused on cost and 30-day readmissions. The authors stated that "measurement of specific quality outcomes are beyond the scope of this study, but the UAB ACE unit implements the same care processes that have been shown to improve outcomes for other ACE units."<br>Reducing 30-day readmissions helps to lower hospital risk for withheld reimbursement.<br>Hospitalisation often leads to adverse outcomes (such as functional decline, delirium, undernutrition, polypharmacy, other iatrogenic events) that lead to higher costs.<br>Improving outcomes while reducing cost is a key objective for the Medicare program and the US as a whole, and the ACE unit was concluded to achieve this. (earlier research had shown that ACE improves processes of care and outcomes)<br>There were significant cost savings for patients with low or moderate CMI. Patients with high CMI had cost-neutral care. "The CMI is defined as the sum of the relative weights of the DRGs for a patient population divided by the number of patients in that population, and it reflects patient complexity and expected resource consumption"

Appendix 6 Synthesis of Outcome Measures

Broad Measure	Measure	Time Frame	Scale/Definition	Category	Indexes
Mortality	(All cause) mortality	Inpatient/at discharge		Clinical	**2,3,6,7,12, 14, 20, 21, 22, 28, 29, 30**
Mortality	(All cause) mortality	30 days post discharge		Clinical	**1,5,7,8, 16, 23**
Mortality	Mortality	2 days post discharge		Clinical	**23**
Mortality	Mortality	7 days post discharge		Clinical	**23**
Mortality	(All cause) mortality	3 months post discharge		Clinical	**5,7, 13**
Mortality	(All cause) mortality	6 months post discharge		Clinical	**5, 28**
Mortality	Mortality	1 year post discharge		Clinical	**7**
Mortality	Mortality			Clinical	**17, 27**
Mortality	Hospital standardised mortality ratio		Actual/Expected Mortality	Clinical	**22**
					**20 Unique Publications**
Time to death	Time to death after discharge	within 30 days, 60 days, 90 days and 1 year after discharge		Clinical	**26**
					**1 Unique Publication**
Readmission	Unplanned Readmission	2 days post discharge	to any public hospital, any reason	Clinical	**23**
Readmission	Unplanned Readmission	7 days post discharge	to any public hospital, any reason	Clinical	**23**
Readmission	Readmission	90 days post discharge	Number, total days, reason, planned/unplanned. To the AGCH (study institution) or hospital	Healthcare Utilisation	**5**
Readmission	Readmission	180 days post discharge	Number, total days, reason, planned/unplanned. To the AGCH (study institution) or hospital	Healthcare Utilisation	**5**
Readmission	Unplanned Readmission	30 days post discharge	7: Emergency Readmission Rate and also noted reason 15: Discharged from and readmitted to the general medicine service under study 24: to any public hospital, any reason	Clinical	**3,6,7, 15, 23**
Readmission	Unplanned Readmission	30 days post discharge	5: Number, total days, reason, planned/unplanned. To the AGCH (study institution) or hospital	Healthcare Utilisation	**5**
Readmission	Readmission	30 days post discharge	21: To one of the general internal medicine services at one of the hospitals involved, 31 and 22: To the same hospital	Clinical	**1,2,8, 16, 17, 22, 24, 27, 28, 29,20, 30, 21**
Readmission	Readmission	72 hours post discharge	To the study hospital	Clinical	**21**

Readmission	Readmission	14 days post discharge	To the study hospital	Clinical	**21**
					**19 Unique Publications**
Length of Stay	Length of Stay	5: at discharge	5: At the AGCH	Clinical	**1**,**2**,**3**,**5**,**7**,**12**, **27**, **28**, **29**
Length of Stay	Acute Length of Stay		14: in the Internal Medical Unit (IMU)	Clinical	**6**, **17**,**14**
Length of Stay	Total length of stay		Including sub-acute (6: including CSC and INMSC)	Clinical	**6, 17**
Length of Stay	Acute Length of Stay	inpatient	total hospital length of stay excluding days spent at an alternate level of care, which refers to patients who no longer need acute care who are awaiting transfer to a different care level (e.g. rehabilitation facility)	Resource Utilisation	**20**
Length of Stay	Length of Stay			Ward Productivity	**9**
Length of Stay	Total length of stay			Clinical	**22**
Length of Stay	Total length of stay		At study institution (an academic medical centre)	Clinical	**24**
Length of Stay	Total length of stay			Resource Utilisation	**30**
Length of Stay	Inpatient days of index care episode			Resource utilisation	**13**
Length of Stay	Length of stay		Nearest hour	Efficiency measure	**21**
					**18 Unique Publications**
Fall related	Consequences of falls	at discharge/inpatient	5: indication for prolonged stay, diagnostics or injury, 7: including inpatient hip fracture, discharge to a new care home, mortality	Clinical	**5,7**
Fall related	Fall history of past 6 months	at discharge		Clinical	**5**
Fall related	Fall history of past 6 months	1 month post discharge		Clinical	**5**
Fall related	Fall history of past 6 months	3 months post discharge		Clinical	**5**
Fall related	Fall history of past 6 months	6 months post discharge		Clinical	**5**
Fall related	Occurrence of falls	during stay/inpatient	5: In the AGCH	Clinical	**5,7**
Fall related	Fear of Falling	At discharge	NRS from 0 to 10	Patient reported	**5**
Fall related	Fear of Falling	1 month post discharge	NRS from 0 to 10	Patient reported	**5**
Fall related	Fear of Falling	3 months post discharge	NRS from 0 to 10	Patient reported	**5**

Fall related	Fear of falling	6 months post discharge	NRS from 0 to 10	Patient reported	**5**
					**2 Unique Publications**
Other adverse effects	Hospital acquired infections			Clinical	**7**
Other adverse effects	Occurrence (presence and duration) of delirium	at discharge (further elaborated that it was assessed at Day 1 to 3 and continued if there were signs of delirium, until symptoms subsided	Confusion assessment method (CAM) four-item short version	Clinical	**5**
Other adverse effects	Occurrence (presence and duration) of delirium	At discharge (further elaborated that it was assessed at Day 1 to 3 and continued if delirium was suspected)	Delirium Observation Screening Scale	Clinical	**5**
Other adverse effects	Risk of developing delirium	At discharge	Dutch Safety Management Program VMS patient screening	Clinical	**5**
Other adverse effects	Pressure sores			Clinical	**7**
Other adverse effects	Potentially preventable complication prompting readmission		Potentially preventable complication of procedure or therapy undertaken/initiated during index admission, prompting readmission	Clinical	**15**
					**3 Unique Publications**
Discharge Destination	Discharge Destination	at discharge		Healthcare Utilisation	**5**
Discharge Destination	Discharge Destination		discharge to home, skilled nursing facility, other facility or inpatient rehabilitation/intermediate care	Clinical	**30**
Discharge Destination	Discharge Destination		discharge to home, transfer to acute hospital, sent to socio-sanitari services	Clinical	**27**
Discharge Destination	Discharge Destination		discharge to own home, usual place of residence, new care home, or institutionalisation	Clinical	**7**
Discharge Destination	Discharge from AGU to other intermediate care units		discharge from AGU to other intermediate care units	Clinical	**12**
Discharge Destination	Discharge to original living situation		discharge to original living situation	Clinical	**12**

					**5 Unique Publications**
Days at residence post discharge	Number of days spent at usual place of residence	90 days post discharge		Clinical	**7**
					**1 Unique Publication**
ADL	ADL	at discharge		Patient reported	**18**
ADL	ADL	30 days post discharge		Patient reported	**18**
ADL	ADL functioning	1 month post discharge	15 item modified Katz ADL scale	Patient reported	**5**
ADL	ADL functioning	3 months post discharge	15 item modified Katz ADL scale	Patient reported	**5**
ADL	ADL functioning	6 months post discharge	15 item modified Katz ADL scale	Patient reported	**5**
ADL	level of dependence	at discharge	Barthel	Patient reported	**28**
					**3 Unique Publications**
Dependency	Need for a type of care package, increased dependency or mobility	at discharge	Validated scale	Clinical	**7**
					**1 Unique Publication**
Pain	Pain	At discharge	NRS from 0 to 10	Patient reported	**5**
Pain	Pain	1 month post discharge	NRS from 0 to 10	Patient reported	**5**
Pain	Pain	3 months post discharge	NRS from 0 to 10	Patient reported	**5**
Pain	Pain	6 months post discharge	NRS from 0 to 10	Patient reported	**5**
					**1 Unique Publication**
Fatigue	Fatigue	At discharge	NRS from 0 to 10	Patient reported	**5**
Fatigue	Fatigue	1 month post discharge	NRS from 0 to 10	Patient reported	**5**
Fatigue	Fatigue	3 months post discharge	NRS from 0 to 10	Patient reported	**5**
Fatigue	Fatigue	6 months post discharge	NRS from 0 to 10	Patient reported	**5**
					**1 Unique Publication**
QoL	HRQoL	At index care episode	HUI-3	Patient reported	**13**

QoL	HRQoL	1 month post discharge	EQ-5D	Patient reported	**5**
QoL	HRQoL	3 months post discharge	EQ-5D	Patient reported	**5**
QoL	HRQoL	6 months post discharge	EQ-5D	Patient reported	**5**
QoL	HRQoL	3 months post discharge	HUI-3	Patient reported	**13**
QoL	QoL		EQ-5D	Patient reported	**7**
QoL	QoL		36 item short-form health survey (SF-36)	Patient reported	**7**
QoL	QoL		Short form 6-D (SF-6D)	Patient reported	**7**
					**3 Unique Publications**
QALY	Quality Adjusted Life Years	3 months post discharge	using HRQoL, with patients' survival time at 3 months	Clinical/Patient reported	**13**
					**1 Unique Publication**
Patient Satisfaction	Patient Satisfaction	at discharge (and 1 month post discharge in case missed out at discharge)	8 questions on a five-point Likert scale	Patient reported	**5**
Patient Satisfaction	Patient Satisfaction		Press-Ganey survey	Patient reported	**21**
Patient Satisfaction	Patient Satisfaction		A user satisfaction survey that assesses a global item	Patient reported	**27**
					**3 Unique Publications**
Patient Experience	Patient Experience	about inpatient stay, taken (26: 1 month, 20: within 2 weeks) after discharge	26: A question from the Picker Patient Experience Questionnaire (PPE-15) – "Overall, how would you rate the care you received at the hospital?" on a 5-point scale<br>20: Picker Patient Experience Questionnaire (PPE-15)	Patient reported	**25,19**
					**2 Unique Publications**
Emotional distress/ psychological burden	Anxiety and Depression		Hospital Anxiety and Depression scale (HADS)	Patient reported	**7**
Emotional distress/ psychological burden	Geriatric Depression		Geriatric depression scale (GDS)	Patient reported	**7**
Emotional distress/ psychological burden	Geriatric Depression		Short form GDS (15 questions)	Patient reported	**7**
Emotional distress/ psychological burden	Apathy	At discharge	3 items of the GDS ((1) 'Do you prefer to stay at home, rather than going out and doing new things', (2) 'Have you dropped	Patient reported	**5**

			many of your activities and interests?' and (3) 'Do you feel full of energy'.)		
Emotional distress/ psychological burden	Apathy	1 month post discharge	3 items of the GDS ((1) 'Do you prefer to stay at home, rather than going out and doing new things', (2) 'Have you dropped many of your activities and interests?' and (3) 'Do you feel full of energy'.)	Patient reported	**5**
Emotional distress/ psychological burden	Apathy	3 months post discharge	3 items of the GDS ((1) 'Do you prefer to stay at home, rather than going out and doing new things', (2) 'Have you dropped many of your activities and interests?' and (3) 'Do you feel full of energy'.)	Patient reported	**5**
Emotional distress/ psychological burden	Apathy	6 months post discharge	3 items of the GDS ((1) 'Do you prefer to stay at home, rather than going out and doing new things', (2) 'Have you dropped many of your activities and interests?' and (3) 'Do you feel full of energy'.)	Patient reported	**5**
					**2 Unique Publications**
Caregiver burden	Caregiver burden		22/12/4/29-item self-reported Zarit Caregiver Burden Interview (ZBI) scale	Caregiver reported	**7**
Caregiver burden	Caregiver burden		24-item Caregiver Burden Inventory (CBI)	Caregiver reported	**7**
Caregiver burden	Caregiver burden		10-item Burden Scale for Family Caregivers	Caregiver reported	**7**
					**1 Unique Publication**
Caregiver satisfaction	Informal caregiver satisfaction	at discharge (and 1 month post discharge in case missed out previously)	8 questions on a five-point Likert scale	Caregiver reported	**5**
					**1 Unique Publication**
Community care costs	Primary care costs	3 months post discharge	doctors' visits, doctors' other contracts, nurse visits, nurse other contacts, other caregivers visits, other caregivers other contacts (other contacts: phone letters, indirect)	Costs	**13**
Community care costs	Municipal care cost	3 months post discharge	home-help services, home health care, days in special housing	Costs	**13**
Community care costs	Costs of informal care		estimated	Costs	**5**
Hospital/Community care costs	Direct medical costs	6 months after admission to the AGCH		Costs	**5**
Hospital costs	Inpatient costs		Total Medicare Part B spending per hospitalisation	Costs	**1, 16, 8**
Hospital costs	Total costs	inpatient (patients were in the ACE/Usual Care unit the entire admission)	total costs are sum of fixed direct costs, variable direct costs and indirect costs	Costs	**30**
Hospital costs	Inpatient costs/Total			Costs	**29, 2**

	Hospitalisation cost				
Hospital costs	Total direct cost			Costs	**3**
Hospital costs	Daily total cost	inpatient (patients were in the ACE/Usual Care unit the entire admission)	total costs are sum of fixed direct costs, variable direct costs and indirect costs	Costs	**30**
Hospital costs	Total variable direct costs	inpatient (patients were in the ACE/Usual Care unit the entire admission)		Costs	**30**
Hospital costs	Daily variable direct costs	inpatient (patients were in the ACE/Usual Care unit the entire admission)	total variable direct cost/LOS	Costs	**30**
Hospital costs	Hospital care cost	3 months after discharge	index care episode, inpatient care, outpatient care	Costs	**13**
Hospital costs	Total costs of the ward			Costs	**9**
Hospital costs	Total costs			Costs (efficiency measure)	**21**
Hospital costs	Amount of hospital resources used for each hospital stay		estimated average; including costs related to administration, staff, supplies, technology and equipment (excluding fee-for-service physician billing costs) using CIHI Resource Intensity Weight for each admission x annual cost per weighted case for acute inpatient cases for each hospital	Costs	**20**
					**12 Unique Publications**
Complaints and litigation costs	Complaints and litigation costs			Others	**7**
					**1 Unique Publication**
Index care episode resource utilisation (other than Length of Stay and transfer to higher acuity care)	Number of bed-days across the patients in the study cohort		For the types of conditions	Resource utilisation	**20**
Index care episode resource utilisation (other than Length of Stay and transfer to higher acuity care)	Designated alternate level of care			Resource utilisation	**20**

Index care episode resource utilisation (other than Length of Stay, and transfer to higher acuity care)	Designated alternate level of care days			Resource utilisation	**20**
Index care episode resource utilisation (other than Length of Stay and transfer to higher acuity care)	diagnostics performed in the AGCH	at discharge		Resource utilisation	**5**
Index care episode resource utilisation (other than Length of Stay and transfer to higher acuity care)	Consults per admission			Resource Utilisation	**3**
Index care episode resource utilisation (other than Length of Stay and transfer to higher acuity care)	Use of advanced imaging		at least 1 of the various imaging tests/scans	Resource Utilisation	**20**
Index care episode resource utilisation (other than Length of Stay and transfer to higher acuity care)	Discharge time (hours after midnight)			Resource utilisation	**3**
Index care episode resource utilisation (other than Length of Stay and transfer to higher acuity care)	Discharge order time (hours after midnight)			Resource utilisation	**3**
Index care episode resource utilisation (other than Length of Stay and transfer to higher acuity care)	Time needed to send medical handovers to the GP at discharge	at discharge		Healthcare Utilisation	**5**
					**3 Unique Publications**

Transfer to higher acuity care	Use of ICU/Transfer to ICU			Clinical	**20**,**21**
Transfer to higher acuity care	Admissions to acute hospital from AGU	during admission		Clinical	**12**
Transfer to higher acuity care	Admissions from AGCH to hospital	at discharge		Healthcare Utilisation	**5**
Transfer to higher acuity care	Revisits from AGCH to hospital	at discharge		Healthcare Utilisation	**5**
					**4 Unique Publications**
ED visits	Number of ED visits	1 month post discharge		Healthcare Utilisation	**5**
ED visits	Number of ED visits	3 months post discharge		Healthcare Utilisation	**5**
ED visits	Number of ED visits	6 months post discharge		Healthcare Utilisation	**5**
					**1 Unique Publication**
Outpatient visits	Number of outpatient hospital visits	1 month post discharge		Healthcare Utilisation	**5**
Outpatient visits	Number of outpatient hospital visits	3 months post discharge	13: outpatient care visits (open care events)	Healthcare/Resource Utilisation	**5**, **13**
Outpatient visits	Number of outpatient hospital visits	6 months post discharge		Healthcare Utilisation	**5**
					**2 Unique Publications**
Inpatient care post-discharge	Inpatient care	3 months post discharge	Index and 3 months post discharge	Resource utilisation	**13**
Inpatient care post-discharge	Inpatient care	3 months post discharge	Days from discharge to follow-up	Resource utilisation	**13**
					**1 Unique Publication**
Home care	frequency of home care	1 month post discharge	Including housekeeping, personal and nursing care and hours of informal care	Healthcare Utilisation	**5**
Home care	Presence and frequency of informal care	1 month post discharge	Presence, frequency per week, type of care (housekeeping and/or personal care) and from which persons (partners, children, other family member or neighbour/volunteers)	Other (Social network and informal care)	**5**
Home care	frequency of home care	3 months post discharge	5: including housekeeping, personal and nursing care and hours of informal care<br>13: Hours of home-help services, home health care hours	Healthcare Utilisation/Resource Utilisation	**5**, **13**

Home care	Presence and frequency of informal care	3 months post discharge	Presence, frequency per week, type of care (housekeeping and/or personal care) and from which persons (partners, children, other family member or neighbour/volunteers)	Other (Social network and informal care)	**5**
Home care	frequency of home care	6 months post discharge	Including housekeeping, personal and nursing care and hours of informal care	Healthcare Utilisation	**5**
Home care	Presence and frequency of informal care	6 months post discharge	Presence, frequency per week, type of care (housekeeping and/or personal care) and from which persons (partners, children, other family member or neighbour/volunteers)	Other (Social network and informal care)	**5**
Home care	Caregiver care	3 months post discharge	other caregivers visits, other caregivers other contacts (other contacts: phone, letters, indirect)	Resource utilisation	**13**
					**2 Unique Publications**
Nursing care post-discharge	Nursing care	3 months post discharge	nurse visits, nurse other contacts (other contacts: phone, letters, indirect)	Resource utilisation	**13**
					**1 Unique Publication**
Primary Care	Number of consultations by GPs	1 month post discharge		Healthcare Utilisation	**5**
Primary Care	Number of consultations by GPs	3 months post discharge	13: includes not just primary care doctors' visits but also doctors other contacts (phone, letters, indirect)	Healthcare Utilisation/Resource Utilisation	**5, 13**
Primary Care	Number of consultations by GPs	6 months post discharge		Healthcare Utilisation	**5**
Primary Care	Unplanned GP consultations			Healthcare Utilisation	**7**
					**3 Unique Publications**
Allied healthcare post-discharge	number of consultations by physiotherapists and dieticians	1 month post discharge		Healthcare Utilisation	**5**
Allied healthcare post-discharge	number of consultations by physiotherapists and dieticians	3 months post discharge		Healthcare Utilisation	**5**
Allied healthcare post-discharge	number of consultations by physiotherapists and dieticians	6 months post discharge		Healthcare Utilisation	**5**
					**1 Unique Publication**
Institutionalisation	permanent institutionalisation	1 month post admission	Long-term admission to a skilled nursing facility	Healthcare Utilisation	**5**

Institutionalisation	permanent institutionalisation	3 months post admission	Long-term admission to a skilled nursing facility	Healthcare Utilisation	**5**
Institutionalisation	permanent institutionalisation	6 months post admission	Long-term admission to a skilled nursing facility	Healthcare Utilisation	**5**
Institutionalisation	temporary admission to a nursing home	1 month post admission	Days of temporary admission to a skilled nursing or rehabilitation facility	Healthcare Utilisation	**5**
Institutionalisation	temporary admission to a nursing home	3 months post admission	Days of temporary admission to a skilled nursing or rehabilitation facility	Healthcare Utilisation	**5**
Institutionalisation	temporary admission to a nursing home	6 months post admission	Days of temporary admission to a skilled nursing or rehabilitation facility	Healthcare Utilisation	**5**
Institutionalisation	Days in special housing	3 months after discharge		Resource Utilisation	**13**
					**2 Unique Publication**
Non-outcome indicators	Number of hospital admissions	21: Annual		Hospital non-outcome indicators (Performance, Efficiency and Effectiveness Indicators)	**20**, **6**
Non-outcome indicators	Beds turnover rate			Hospital non-outcome indicators (Ward productivity)	**9**
Non-outcome indicators	Number of beds			Hospital non-outcome indicators (management indicators for the ward- proxies for complexity)	**14**
Non-outcome indicators	Case Mix Index		clinical complexity calculated as the ratio between the weight of admissions for each category of patients and the mean weight of admissions for the same category by all regional providers during the same year	Hospital non-outcome indicators (Performance, Efficiency and Effectiveness Indicators)	**6**
Non-outcome indicators	Charlson-Deyo Index/Mean Charlson Index			Hospital non-outcome indicators (Performance, Efficiency and Effectiveness Indicators/Management indicators for the ward- proxies for complexity)	**6**, **14**
Non-outcome indicators	Comparative Performance Index (CPI)		operational efficiency of hospital units obtained from average LOS standardised for case-mix and weighted for the efficiency of the other hospitals in the same healthcare system	Hospital non-outcome indicators (Performance, Efficiency and Effectiveness Indicators)	**6**

Non-outcome indicators	HDR Average Weight		ratio between DRG points produced in a specific discipline of the considered hospital and the number of discharged patients	Hospital non-outcome indicators (Performance, Efficiency and Effectiveness Indicators)	**6**
Non-outcome indicators	Mean DRG Weight		14: of IMU DRG at IMU level	Hospital non-outcome indicators (Ward Productivity/Management indicators for the ward-proxies for complexity)	**9, 14**
Non-outcome indicators	Multidisciplinary ward rounds		at IMU level	Hospital non-outcome indicators (management indicators for the ward-proxies for complexity)	**14**
Non-outcome indicators	Patient Safety Committee		at IMU or hospital level	Hospital non-outcome indicators (management indicators for the ward-proxies for complexity)	**14**
Non-outcome indicators (for the proportion of hospital resources used by general IM patients)	proportion of ED admissions that were general internal medicine.			Hospital non-outcome indicators	**20**
Non-outcome indicators (for the proportion of hospital resources used by general IM patients)	proportion of total bed days that were general internal medicine.			Hospital non-outcome indicators	**20**
Non-outcome indicators (for the proportion of hospital resources used by general IM patients)	proportion of total hospital admissions that were general internal medicine admissions			Hospital non-outcome indicators	**20**
Non-outcome indicators	number of inhabitants in the influence area			Hospital non-outcome indicators (management indicators for the ward-proxies for complexity)	**14**
					**4 Unique Publications**
Staff experience	self-reported resident work hours			Staff reported	**21**
Staff experience	Regular staff surveys		About work experience: a yearly appraisal, satisfaction with quality of work, discrimination, harassment, or abuse at work,	Staff reported	**7**

			the level of pay, flexible working, and equal work opportunities.		
Staff experience	nurse burnout levels, nurse job dissatisfaction or satisfaction level of the staff, work-related stress, needle-stick injuries, happiness level		Various combinations of these and the indicators in the following row	Staff reported	**7**
Staff experience	Nursing Practice environment		Practice Environment Scale of the Nursing Work Index (PES-NWI)	Staff reported	**7**
					**2 Unique Publications**
Staff management indicators	Staffing ratios, sickness rate, recruitment and retention of staff, gaps in the nursing rota, nurses’ skill mix		Various combinations of these and the staff experience indicators in the previous row	Staff management indicators	**7**
					**1 Unique Publication**
